# Upcycling aromatic polymers through C–H fluoroalkylation†
†Electronic supplementary information (ESI) available: Materials and detailed synthesis, characterization, optimization studies, and supporting figures. See DOI: 10.1039/c9sc01425j


**DOI:** 10.1039/c9sc01425j

**Published:** 2019-05-29

**Authors:** Sally E. Lewis, Bradley E. Wilhelmy, Frank A. Leibfarth

**Affiliations:** a Department of Chemistry , University of North Carolina at Chapel Hill , 125 South Rd , Chapel Hill , NC 27599 , USA . Email: Frankl@unc.edu

## Abstract

The unique properties imparted by planar, rigid aromatic rings in synthetic polymers make these macromolecules useful in a range of applications, including disposable packaging, aerospace materials, flexible electronics, separation membranes, and engineering thermoplastics. The thermal and chemical stability of aromatic polymers, however, makes it difficult to alter their bulk and/or surface properties and results in challenges during recycling. In response, we report a platform approach for the C–H functionalization of aromatic polymers by taking advantage of their innate reactivity with electrophilic radical intermediates. The method uses mild reaction conditions to photocatalytically generate electrophilic fluoroalkyl radicals for the functionalization of an array of commercially relevant polyaromatic substrates, including post-industrial and post-consumer plastic waste, without altering their otherwise attractive thermomechanical properties. The density of fluorination, and thus the material properties, is tuned by either increasing the reagent concentration or incorporating longer perfluoroalkyl species. Additionally, the installation of versatile chemical functionality to aromatic polymers is demonstrated through the addition of a bromodifluoromethyl group, which acts as an initiator for atom transfer radical polymerization (ATRP) grafting of vinyl polymers. The method described herein imparts new and versatile chemical functionality to aromatic polymers, enabling an efficient approach to diversify the properties of these otherwise recalcitrant commodity plastics and demonstrating a viable pathway to upcycle post-consumer plastic waste.

## Abstract 2

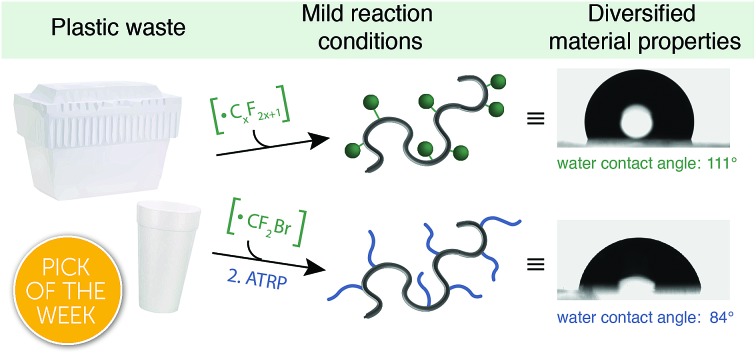
This work provides a platform C–H functionalization method that introduces fluoroalkyl groups onto commercial aromatic polymers and post-consumer plastic waste that improve their material properties.

## Introduction

Synthetic polymers that contain aromatic rings, including polystyrene, poly(ethylene terephthalate), polycarbonate, and high performance polymers, are omnipresent in modern materials.^1^–^6^ The range of applications for these plastics is a testament to the unique properties imparted by planar and rigid aromatic moieties. A common approach to tune the bulk and/or surface properties of these materials is copolymerization, where a comonomer that contains the desired functionality is included in the polymerization.^6^ Copolymerization of high-volume polymers, however, requires re-optimization of polymerization processes and can necessitate supplanting synthetic infrastructure on-scale, which is both cost- and labor-intensive.^7^ Furthermore, copolymerization strategies often suffer from disparate monomer reactivity, resulting in anisotropic material properties.

Post-polymerization modification (PPM) of high-volume aromatic polymers is an appealing approach to modify material properties.^8^ A general PPM strategy could diversify the properties of a wide-variety of commercial polymers while leveraging existing industrial infrastructure. Additionally, the development of PPM methods that increase the value of post-industrial and/or post-consumer aromatic polymers would provide a platform strategy for the upcycling of plastic waste. This upcycling approach is especially relevant for aromatic materials that are traditionally difficult and cost-prohibitive to mechanically recycle, such as expanded polystyrene (EPS) foam and high-performance polymers.^9^–^12^

Exploiting the innate reactivity of aromatic polymers has the potential to introduce a wide array of chemical functionality while retaining the beneficial properties of the parent polymer. Electrophilic radicals represent reactive intermediates that are well-known to undergo formal C–H functionalization with electron-rich or electron-neutral aromatic rings.^13^ Despite a renewed interest in these species for the functionalization of small molecules,^14^–^16^ general methods that enable functionalization of aromatic polymers using electrophilic radicals remains underexplored.

Electrophilic radical-mediated functionalization of aromatic polymers has historically focused on polymer halogenation. Radical bromination of polystyrene (PS) was first reported by Staudinger and subsequently studied by a number of groups and developed into a commercial process.^17^–^21^ Direct fluorination of aromatic polymers using fluorine gas was later reported by Lagow and coworkers to yield partially or perfluorinated materials.^22^ These and other halogen-centered radicals engage in a number of reactions with aromatic polymers, including hydrogen atom abstraction and aromatic substitution. The poor chemoselectivity of these approaches result in significant polymer chain-cleavage and chain-coupling upon halogenation, thus compromising their otherwise attractive thermomechanical properties.

Functionalization of aromatic polymers by electrophilic perfluoroalkyl radicals, however, has the potential to be a mild and chemoselective route for PPM.^23^ The pyramidal ground state of the trifluoromethyl radical and its low-lying singly-occupied molecular orbital results in rapid addition to electron-rich alkenes or aromatic systems as opposed to participating in deleterious hydrogen-atom abstraction or radical recombination reactions.^13^ In seminal work, Shuyama reported the fluoroalkylation of poly(α-methylstyrene) using (perfluoroethyl)phenyliodonium triflate to achieve 24 perfluoroethyl groups per 100 repeat units, or 24 mol% functionalization.^24^ Subsequently, Sawada and coworkers pioneered the use of perfluoroacyl peroxides (PFAPs) as fluoroalkyl radical precursors for the functionalization of a variety of aromatic substrates.^25^ Notably, PFAP-mediated trifluoromethylation of polystyrene resulted in 80 mol% functionalization. This approach, however, was accompanied by a coincident increase in the molecular weight distribution (MWD) of the polymer due to polymer chain coupling, with the dispersity (*Đ*) of polystyrene increasing from 1.06 to 2.41 at 80 mol% functionalization.^26^ Furthermore, PFAPs must be formed and used immediately due to their rapid and sometimes violent decomposition at temperatures above 0 °C.^27^ A modular, tunable, and user-friendly method for the fluoroalkylation of aromatic polymers that maintains the beneficial properties of the parent material remains underdeveloped.^28^

**Fig. 1:**
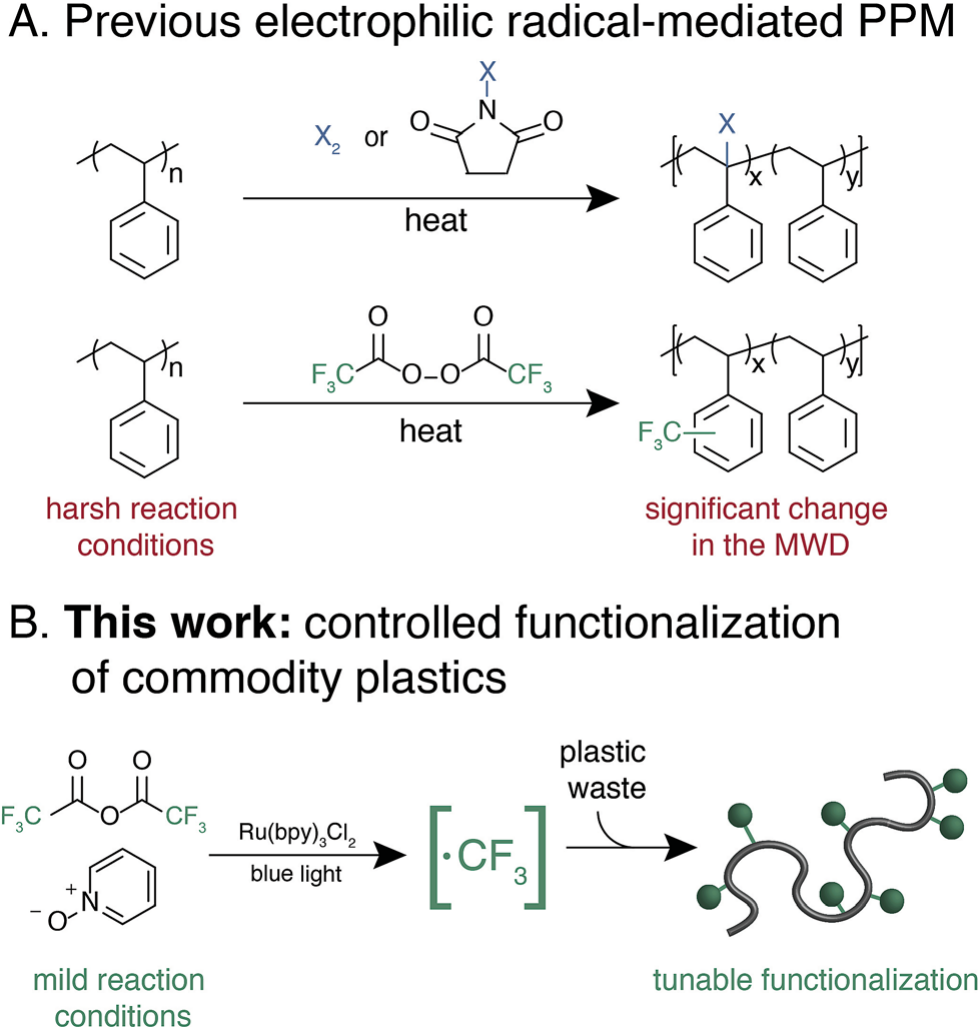
Approaches for the functionalization of aromatic polymers with electrophilic radicals. (A) PPM *via* radical halogenation and trifluoromethylation using perfluoroacyl peroxides as a precursor to trifluoromethyl radicals. (B) Our method accesses electrophilic radicals to functionalize a variety of commodityaromatic polymers.

We envisioned an approach that utilizes electrophilic radicals not only to fluorinate materials, but also to impart additional functionality onto commodity aromatic polymers to diversify their physical properties. The benefits of fluoroalkyl groups in medicinal chemistry have spurred the development of a variety of approaches for the generation of electrophilic fluoroalkyl radical reactive intermediates.^13^,^29^–^37^ We identified a photocatalytic method developed by Stephenson and coworkers as an attractive system for polymer substrates.^38^–^40^ This approach demonstrated mild reaction conditions, tolerance to a wide variety of functional groups, operationally simple reaction set-up, and low-cost reagents. Driven by the need for a chemoselective method to diversify the properties of commodity aromatic polymers, we sought to expand this photocatalytic strategy for fluoroalkylation of macromolecules (Fig. 1).

We report a platform approach for the C–H functionalization of aromatic polymers that enhances their physical properties through the use of electrophilic radical reactive intermediates. Photocatalytic generation of electrophilic fluoroalkyl radicals under mild reaction conditions enables chemoselective polymer functionalization with no evidence of deleterious chain coupling or chain scission side reactions. The approach takes advantage of the innate reactivity of aromatic polymers, providing a platform for the functionalization of a variety of commercially valuable commodity polymers as well as post-industrial and post-consumer plastic waste. Electrophilic radicals generated from both short- and long-chain perfluoroalkyl acid anhydrides enable a tunable density of fluorine to be installed under operationally simple reaction conditions. Furthermore, installation of a bromodifluoromethyl group imparts functionality that serves as an initiator for atom transfer radical polymerization (ATRP), providing a facile approach for the chemical diversification of commodity polymers.^41^ The versatile strategy described herein imparts valuable functionality onto otherwise recalcitrant commercial and waste polymers through C–H functionalization, providing opportunities to add value to these pervasive materials and upcycle post-consumer plastic waste.

## Results & discussion

The photocatalytic method reported by Stephenson for the perfluoroalkylation of small molecule substrates provided a conceptual framework to access fluoroalkyl radicals. Translating the method to a successful polymer functionalization strategy, however, faced considerable hurdles. The modest yield for the trifluoromethylation of non-functional substrates such as benzene and mesitylene indicates that not all fluoroalkyl radicals are participating in productive reactivity. Additionally, the steric environment of a random coil polymer in solution could limit the extent of functionalization. To probe structure–reactivity relationships for the fluoroalkylation of aromatic polymers, we chose trifluoroacetic anhydride (TFAA) due to its simple ^19^F NMR signature and polystyrene as a model substrate due to its high-volume production and challenges in recycling streams.

PS substrate **1**, designed and synthesized *via* ATRP to contain a trifluoromethyl group at the chain end, was used for optimization studies. The chain end served as a polymer-bound internal standard for quantification of trifluoromethylation by ^19^F NMR. The narrow dispersity of the model polymer (*Đ* = 1.04) enabled the determination of subtle changes in the number-average molar mass (*M*_n_) and *Đ* under the reaction conditions.

**Fig. 2:**
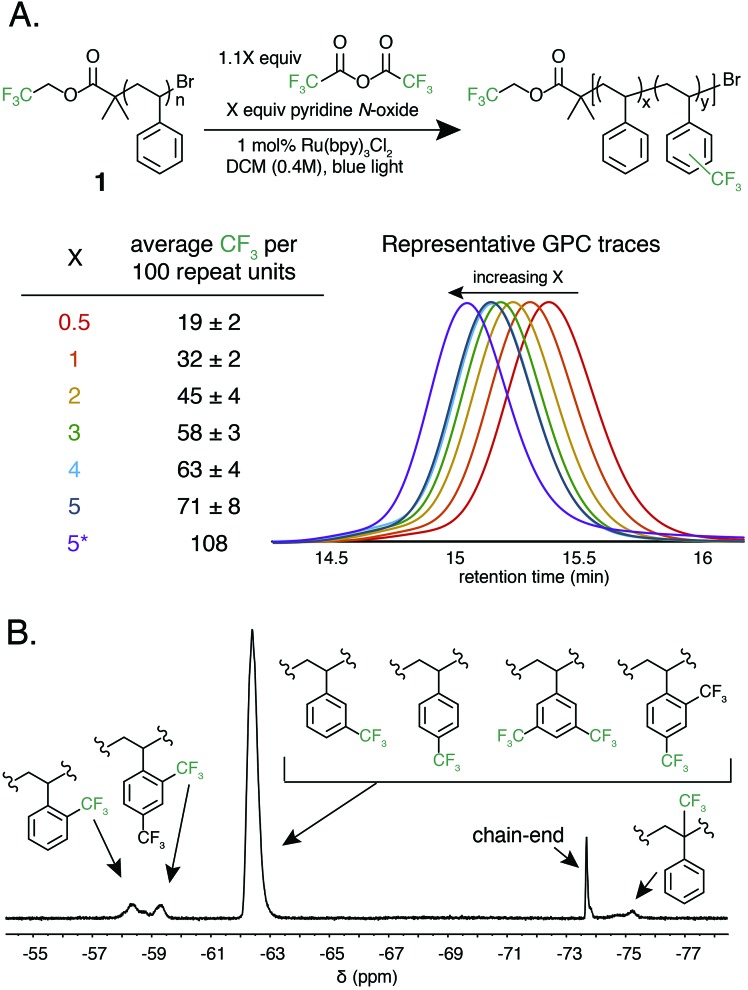
Reaction conditions for the trifluoromethylation of **1**. (A) Results of polystyrene trifluoromethylation at various amounts of pyridine N-oxide and TFAA equivalents compared to repeat unit. Each set of conditions was performed in triplicate to give a standard deviation. Inset demonstrates example GPC chromatograms at each condition. (B) A representative ^19^F NMR spectra of the regiorandom trifluoromethylation of **1**. Proposed peakassignments were determined by small molecule and copolymerization studies (Fig. S1†). *The reaction was isolated from the experiment where *X* = 5 and resubjected to the reaction conditions.

Trifluoromethylation of **1** was achieved by combining 1.0 equivalent pyridine N-oxide and 1.1 equivalents of TFAA relative to repeat unit, and 1.0 mol% Ru(bpy)_3_Cl_2_ relative to pyridine N-oxide in dichloromethane (DCM) at room temperature under 420 nm blue light irradiation. The reaction resulted in 32 ± 2 mol% trifluoromethylation (Fig. 2A). An analogous reaction performed in the dark resulted in no trifluoromethylation of **1**, and performing the reaction under irradiation without the Ru(bpy)_3_Cl_2_ photocatalyst resulted in only 7 mol% trifluoromethylation. The reaction performed equally well in the presence of oxygen or with rigorous exclusion of oxygen, which simplified reaction setup. A catalyst loading of 1.0 mol% proved to be optimal, as both decreasing and increasing the relative amount of Ru(bpy)_3_Cl_2_ resulted in decreased trifluoromethylation. Alternative solvents and lower or higher concentrations resulted in inferior reaction performance. A complete reaction optimization table can be found in the ESI (Table S1†).

Varying the stoichiometry of TFAA and pyridine N-oxide compared to repeat unit provided a tunable level of polymer C–H trifluoromethylation. The addition of 0.5 equivalents of pyridine N-oxide and 0.55 equivalents of TFAA compared to repeat unit of **1** resulted in an average of 19 ± 2 mol% functionalization, while the addition of 5.0 and 5.5 equivalents, respectively, resulted in 71 ± 8 mol% functionalization (Fig. 2A). Increasing reagent equivalents above 5.0 resulted in decreased mol% functionalization, likely due to separation of a TFAA phase from the rest of the reaction components. Resubmitting material with 71 mol% functionalization to the reaction conditions further increased trifluoromethylation to an average of 108 CF_3_ units per 100 styrene repeat units, which we attribute to the addition of two trifluoromethyl groups to a single styrene repeat unit. This high density of functionalization is rarely observed in C–H functionalization reactions on commodity polymers^8^ and speaks to the efficiency of the method.

Following trifluoromethylation, gel permeation chromatography (GPC) demonstrated a shift to shorter retention times, indicating an increase in polymer molecular weight associated with a change in the hydrodynamic radius of the polymer upon functionalization (Fig. 2A). The *Đ* of the polymer changes very little even after exposure to large excesses of the reagent, highlighting the chemoselective reaction conditions. This trifluoromethylation approach is in contrast to previous work using PFAPs, which uniformly reported a significant increase in *Đ* that scaled with the relative concentration of the acyl peroxide fluorinating agent employed in the reaction.

^19^F NMR provided quantitative evidence of polystyrene trifluoromethylation. New peaks in the ^19^F NMR spectrum after the reaction demonstrated regiorandom polystyrene trifluoromethylation (Fig. 2B). The synthesis of a number of well-defined copolymers and small molecules facilitated peak assignments (Fig. S1†). Previous work has demonstrated that alkyl groups on an aromatic ring direct radical trifluoromethylation *ortho*/*para* due to inductive effects.^37^,^38^,^42^ We hypothesize that the broad singlet at –62 ppm encompasses both *para* and *meta* functionalization of a styrene repeat unit. The two broad peaks between –57 and –60 ppm represent *ortho* trifluoromethylation, with the upfield-most peak being assigned to the *ortho* trifluoromethyl group in a repeat unit that contains both *ortho* and *para* bistrifluoromethylation. This hypothesis was confirmed through the synthesis and characterization of an electronically similar small-molecule analogue, 2′,4′-bis(trifluoromethyl)cumene. As functionalization increases, the number of rings that include two trifluoromethyl groups also increases (Fig. S2†). Lastly, benzylic functionalization is also observed and corresponds to less than 4 mol% of the functionalized repeat units even at high reagent loadings (5.0 equivalents of pyridine N-oxide and 5.5 equivalents of TFAA) (Table S2†).

Thermal properties of trifluoromethylated polymers were investigated. Polystyrene **1** had a decomposition temperature (*T*_D_), measured by thermal gravimetric analysis (TGA) where the polymer lost 10% of its initial mass, of 329 °C. Trifluoromethylation did not significantly alter the *T*_D_ of the materials, confirming the thermal stability of the functionalized polymers. The influence of polystyrene trifluoromethylation on the glass transition temperature (*T*_g_) was analysed by differential scanning calorimetry (DSC), with data taken from the second heating cycle at a ramp rate of 10 °C per minute. The *T*_g_ of **1** lowered slightly from 84 °C to 75 °C at 45 mol% functionalization. Increasing the density of trifluoromethylation to 63 mol% did not significantly influence thermal behaviour (*T*_g_ = 80 °C). The application of Gordon–Taylor theory to previously measured values for the *T*_g_s of trifluoromethylstyrene homopolymers fits qualitatively with the observed results (Table S3†).

**Fig. 3:**
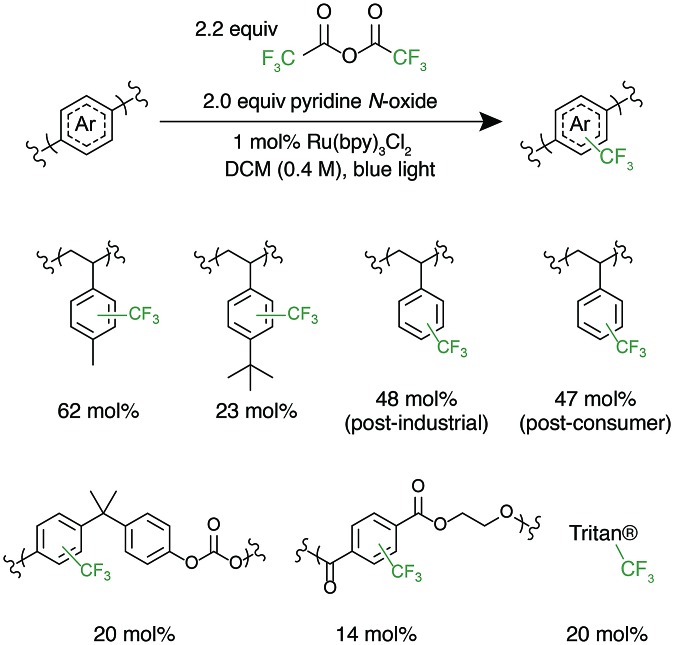
Mol% trifluoromethylation of the repeat unit of commercial aromatic polymers. For poly(bisphenol A carbonate) 20 mol% of repeat unit is functionalized indicating that 10 mol% of the aryl rings are functionalized.

After successful polystyrene trifluoromethylation, this methodology was expanded to other commercially valuable aromatic polymer substrates (Fig. 3). These reactions were run at 2.0 equivalents of pyridine N-oxide and 2.2 equivalents of TFAA compared to polymer repeat unit. For reference, the reaction achieved 45 mol% trifluoromethylation of **1** under the same conditions. Functionalization reactions of poly(4-methylstyrene) and poly(4-*tert*-butylstyrene) were successful, achieving 63 and 23 mol% trifluoromethylation, respectively. Poly(4-*tert*-butylstyrene) demonstrated a lower mol% functionalization than PS under analogous conditions, presumably due to steric hindrance. Poly(4-methylstyrene), however, achieved a considerably higher degree of functionalization of 62 mol%, presumably due to the additional electron density of the aromatic ring.

The introduction of electronegative functional groups to polymers made by step-growth polymerization is valuable due to the difficulty of incorporating electronically dissimilar monomers into a copolymerization. Polyesters and polycarbonates are the highest-volume polymers produced by step-growth polymerization; thus, representative substrates from these polymer classes were prioritized for trifluoromethylation.^43^,^44^ These reactions were run at 2.0 equivalents of pyridine N-oxide and 2.2 equivalents of TFAA compared to repeat unit. Exposure of bisphenol A-derived polycarbonate to the reaction conditions resulted in 10 mol% functionalization of the repeat units. Further, trifluoromethylation of commercial polyesters including poly(ethylene terephthalate) (PET) and Eastman's Tritan® copolyester resulted in 14 and 20 mol% functionalization, respectively. For both polycarbonates and polyesters, we hypothesize that the lower mol% trifluoromethylation compared to polystyrene is a result of the electron poor nature of the aromatic ring.

Following the successful C–H fluoroalkylation of structurally diverse polymer substrates, the functionalization of post-industrial and post-consumer plastic waste was tested. Polystyrene was selected as a substrate for these studies due to the difficulties in recycling EPS foam.^10^–^12^ Post-industrial EPS foam waste with a *M*_n_ of 71 kg mol^–1^ and *Đ* of 2.92 generated as a by-product during production of foam packaging was procured. Post-consumer polystyrene with a *M*_n_ of 75 kg mol^–1^ and *Đ* of 2.85 was secured from an EPS foam cooler that would otherwise have been disposed. Both post-industrial and post-consumer polystyrene were exposed to the reaction conditions at 2.0 equivalents of pyridine N-oxide and 2.2 equivalents of TFAA. These materials demonstrated analogous reactivity and mol% functionalization to a sample of pristine polystyrene and had no significant change in MWD after functionalization (Fig. S6 and S7†). These experiments prove that the formulation, processing, and use of post-industrial and post-consumer EPS foam does not have a substantial influence on polymer C–H fluoroalkylation. These results further display the chemoselectivity and efficiency of functionalization by electrophilic radicals and indicate the potential for C–H fluoroalkylation to upcycle post-consumer plastic waste.

**Fig. 4:**
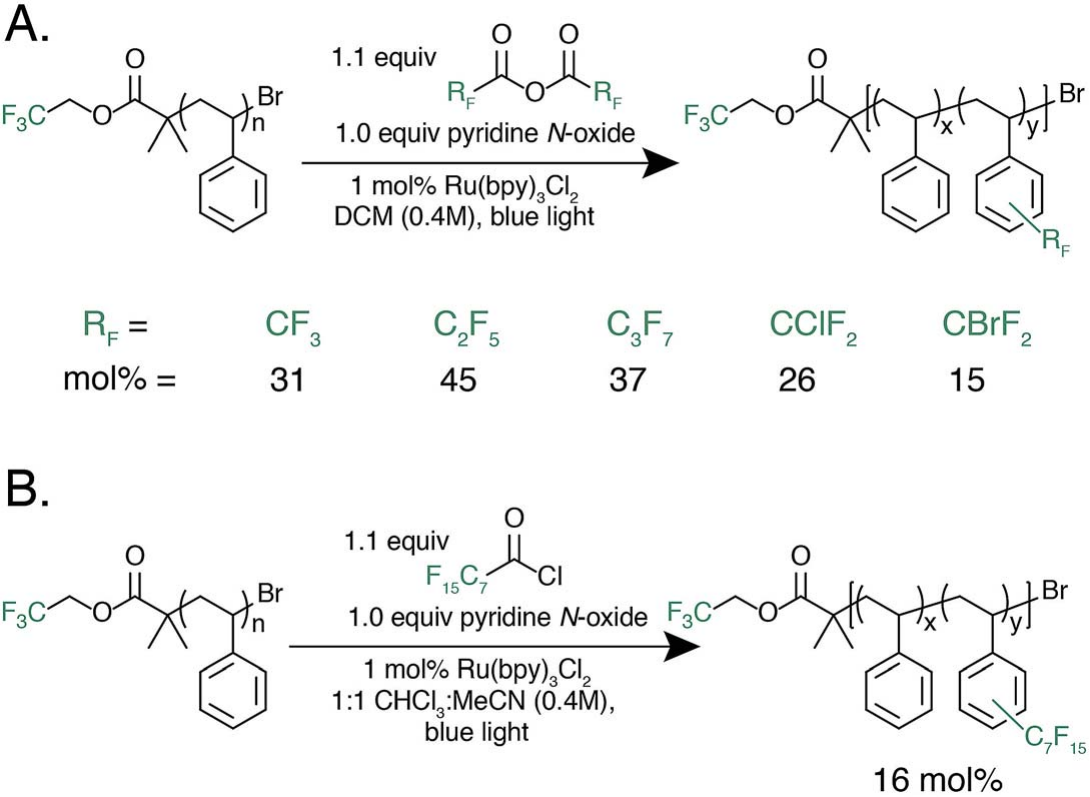
Alternative perfluoroalkyl sources for polymer functionalization. (A) Fluoroalkylation with alternative, commercially available fluoroalkyl anhydrides, including perfluoroethyl-, perfluoropropyl, chlorodifluoromethyl-, and bromodifluoromethylated was successfully performed. (B) Fluoroalkyl acyl chlorides are also able to act as fluorinating reagents, demonstrated by the use of pentadecafluorooctanoyl chloride.

With photocatalytic C–H trifluoromethylation established on a variety of polymer substrates, the generality of the method to incorporate longer perfluoroalkyl groups was investigated. Commercially available pentafluoropropionic anhydride and heptafluorobutyric anhydride served as fluoroalkylating agents in place of TFAA (Fig. 4A). Functionalization of **1** using 1.0 equivalent of pyridine N-oxide and 1.1 equivalent of the short-chain perfluoro anhydride proceeded to produce polymers achieving 45 mol% perfluoroethylation and 37 mol% perfluoropropylation. To covalently attach perfluorochains of longer length, commercially available perfluorooctanoyl chloride was employed (Fig. 4B). Use of this reagent successfully added perfluoroheptyl groups to polystyrene, albeit at lower efficiency than for shorter-chain perfluorocarbons, achieving 16 mol% at 1.0 equivalent of pyridine N-oxide and 1.1 equivalents of the acyl chloride.

The conceptual approach of C–H functionalization using electrophilic radicals also allows the incorporation of functional groups capable of further synthetic manipulations. Specifically, we targeted the incorporation of chlorodifluoromethyl and bromodifluoromethyl groups onto aromatic polymers due to the versatile chemistry of the halomethyl groups (Fig. 4A).^40^ The commercially available acetic anhydrides were used for the functionalization of **1**, resulting in group transfer of the corresponding halodifluorocarbon onto polystyrene. Using 1.1 equivalent of the anhydride and 1.0 equivalent of pyridine N-oxide to repeat unit, chlorodifluoromethylation (26 mol%) proceeded with similar efficiency to trifluoromethylation (32 mol%), however the bromodifluoromethylation was less efficient (15 mol%) due to the reduced electrophilicity of the bromodifluoromethyl radical.

To demonstrate the potential of halodifluoromethyl groups as a functional handle, the bromodifluoromethyl-functionalized polystyrene was explored for further chemical diversification. We hypothesized that the lower bond dissociation free energy (BDFE) of the carbon–bromine bond would enable selective functionalization through radical chemistry. The difluoromethyl group is of emerging interest in medicinal chemistry due to its ability to act as a hydrophobic hydrogen-bond donor,^45^–^48^ but its potential has rarely been studied in polymer science.^49^–^52^ Bromine-atom abstraction initiated by azobisisobutyronitrile (AIBN) in the presence of tributyltin hydride as a hydrogen atom transfer reagent led to quantitative conversion of the bromodifluoromethyl group to a difluoromethyl group. Characterization by ^19^F NMR demonstrated an upfield shift of the peak representing the difluoromethyl group after reaction, indicating 98% conversion to the desired functionality (Fig. S30†).

**Fig. 5:**
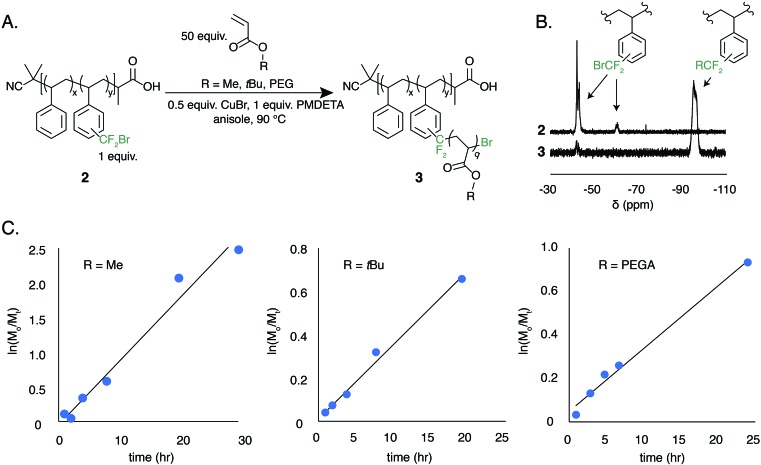
ATRP-mediated graft copolymerization of acrylate monomers initiated by a polymer-bound bromodifluoromethyl functional group (**2**). (A) General reaction scheme of grafting copolymerizations. (B) A representative ^19^F NMR taken at *t* = 8 h of the poly(styrene-*graft*-methyl acrylate) copolymer synthesis demonstrating the initiation efficiency of the bromodifluoromethyl group. (C) Kinetic profiles for the growth of poly(styrene-*graft*-MA), poly(styrene-*graft-t*BuA), and poly(styrene-*graft*-PEGA).

The low BDFE of the carbon–bromine bond and the previous use of bromotrifluoromethane as an initiator for free radical polymerization led us to hypothesize that the bromodifluoromethyl group could serve as a polymer-bound ATRP initiator.^53^–^55^ To test this hypothesis, a well-defined polystyrene was made using reversible addition–fragmentation chain transfer (RAFT) polymerization. The trithiocarbonate end group was removed and the polymer was functionalized to contain 7.8 mol% bromodifluoromethyl groups (**2**, *M*_n_ = 13 kg mol^–1^, *Đ* = 1.19) (Fig. 5A).

The ATRP grafting of methyl acrylate (MA), *tert*-butyl acrylate (*t*BuA), and poly(ethylene glycol) acrylate (PEGA) from polymer **2** was studied. Using 0.5 equivalents of CuBr relative to the bromodifluoromethyl initiator and 1.0 equivalent of PMDETA as a ligand at a 1 : 1 v/v ratio of monomer to anisole at 90 °C, graft copolymers of poly(styrene-*graft-t*BuA), poly(styrene-*graft*-MA), and poly(styrene-*graft*-PEGA) (**3**) were produced (Fig. 5A). The kinetic data indicates well controlled polymerizations, demonstrating a linear increase in monomer conversion over time (Fig. 5C and S8–S10†). Additionally, the ^19^F NMR indicates full initiation of each CF_2_Br appended to polystyrene (Fig. 5B). TGA and DSC characterization further indicate graft copolymer products (Fig. S11†). Poly(styrene-*graft-t*BuA), for example, showed the diagnostic thermal expulsion of isobutylene from poly(*t*BuA) beginning at 188 °C by TGA, with polymer decomposition occurring at 406 °C. The DSC data reveals that the *T*_g_ decreases as the poly(*t*BuA) side-chains increase in molar mass, lowering from 79 °C with 3.6 repeat units per side chain to 50 °C with 24 repeat units per side chain (Fig. S11†). These *T*_g_s are between that of polystyrene and poly(*t*BuA) and further confirm the covalent connection between the two otherwise immiscible polymers. To further demonstrate the versatility of these graft copolymers, the *tert*-butyl groups were cleaved through acid treatment to produce the amphiphilic poly(styrene-*graft*-acrylic acid) copolymers.

**Fig. 6:**
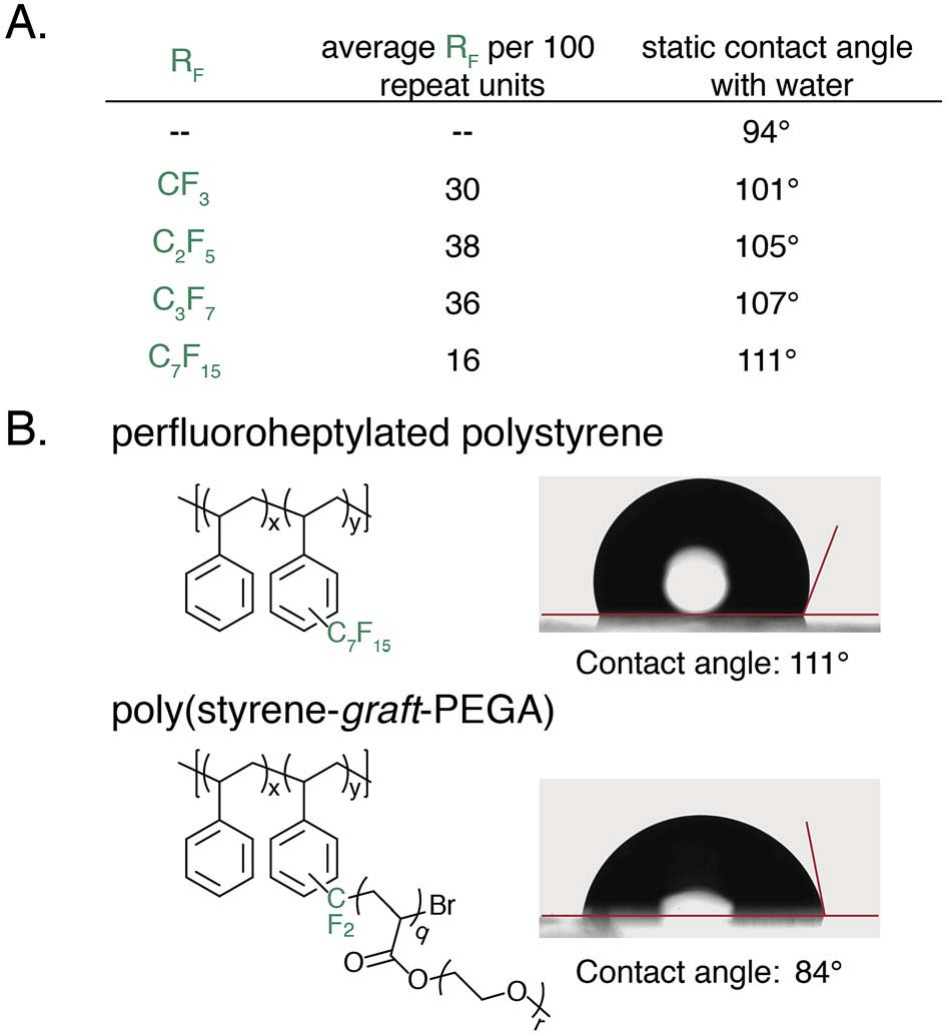
Contact angle measurements of synthesized polymer films. (A) Highest achieved static water contact angles of perfluoroalkylated **1**. (B) Contact angle images of perfluoroheptylated **1** and poly(styrene-*graft*-PEGA) copolymer comb.

An advantage of polyaromatic C–H fluoroalkylation is its potential to modify the surface and/or interfacial properties of commodity polymers.^33^,^56^–^62^ Fluorinated polymers are well known to demonstrate high surface energy, and covalent bonding of perfluoroalkyl chains to a material is an attractive approach to prevent the leaching of physically absorbed perfluoroalkyl coatings into the environment.^63^,^64^ The static contact angle of water and *n*-hexadecane was used to determine qualitative changes in surface energy upon polymer C–H fluoroalkylation. To measure contact angles a 20 wt% solution of functionalized polystyrene in toluene was spin coated onto glass slides, annealed at 140 °C for 30 min, and the contact angle was measured by goniometry. For each perfluoroalkyl group, the static water contact angle increased from that of the native polystyrene **1** (94°) (Fig. 6A). We found that approximately 30 mol% functionalization of the polymers was sufficient to impart properties significantly different from that of polystyrene, and further functionalization did not alter the surface properties. As the chain-length of the perfluoroalkyl group increased, the static water contact angle increased in a commensurate fashion up to 111° for perfluoroheptylated polystyrene (Fig. 6A). In addition to discovering water repellent polymers, fluoroalkylated polystyrene are also more omniphobic. While *n*-hexadecane fully wets a PS surface, the contact angle of *n*-hexadecane on a 64 mol% perfluoropropylated surface is 37°. Conversely, the ability to graft PEGA from bromodifluoromethyl-functionalized polystyrene enabled the realization of hydrophilic materials. For example, poly(styrene-*graft*-PEGA) copolymer becomes water soluble at high conversions of the PEG-containing graft polymer. At low conversion, the graft copolymer made films that demonstrated hydrophilic surface properties, with a static water contact angle of 84° (Fig. 6B).

## Conclusion

The work presented herein provides a platform C–H functionalization method that introduces fluoroalkyl groups onto commercial aromatic polymers. The applicability of this method to a wide variety of commercially available fluoroalkyl anhydrides and acyl chlorides, its broad substrate scope for both commodity and post-consumer aromatic polymers, and its utility for diversifying the properties of traditionally recalcitrant materials makes it attractive for applications in materials science and upcycling plastic waste.

## Conflicts of interest

There are no conflicts to declare.

## Supplementary Material

Infographic

Supplementary information
