# Supplementary material for: Upcycling aromatic polymers through C–H fluoroalkylation
Source: Chem Sci. 2019 May 29;10(25):6270–7. doi: 10.1039/c9sc01425j (PMC6601422; doi:10.1039/c9sc01425j)
Supplement: Supplementary file 1 [file SC-010-C9SC01425J-s001.pdf]

Electronic Supplementary Material (ESI) for Chemical Science.
This journal is © The Royal Society of Chemistry 2019

Waste management aced: Modifying non-recyclable plastics into upcycled products

Plastics (long-chain polymers) are omnipresent and recycling them is difficult, which results in their accumulation in the environment and pollution.

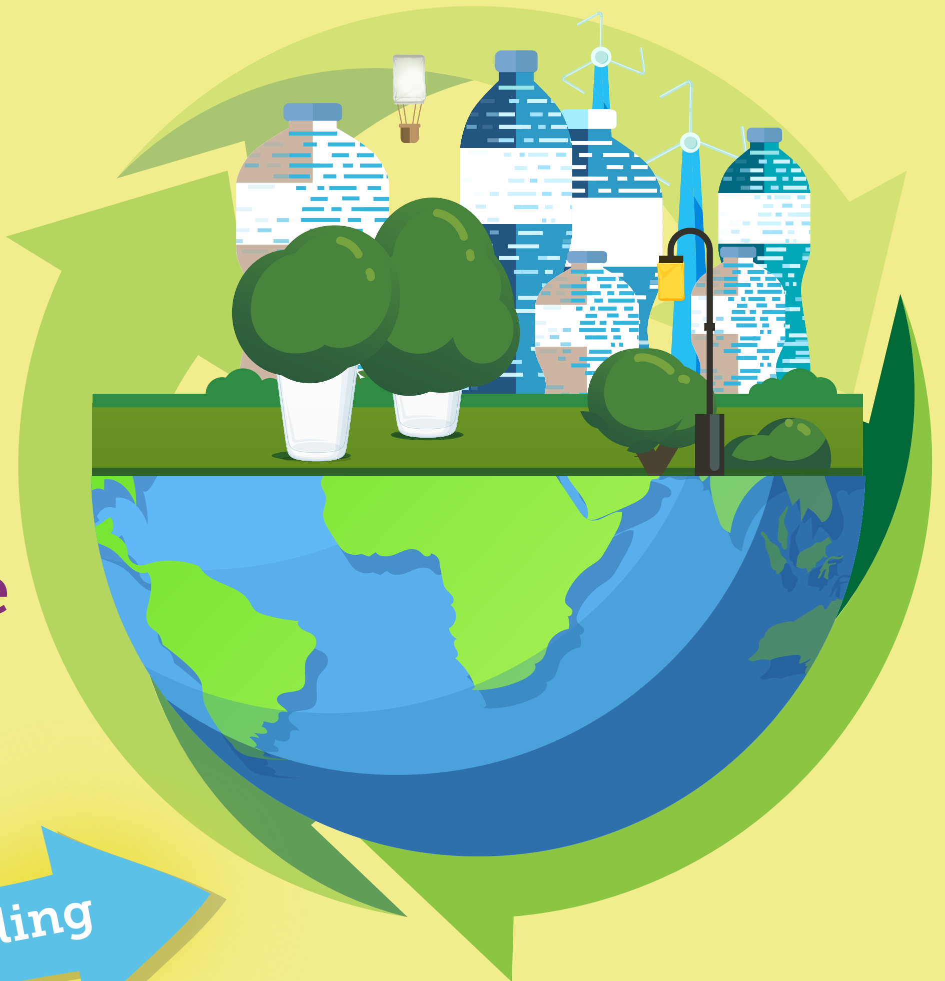

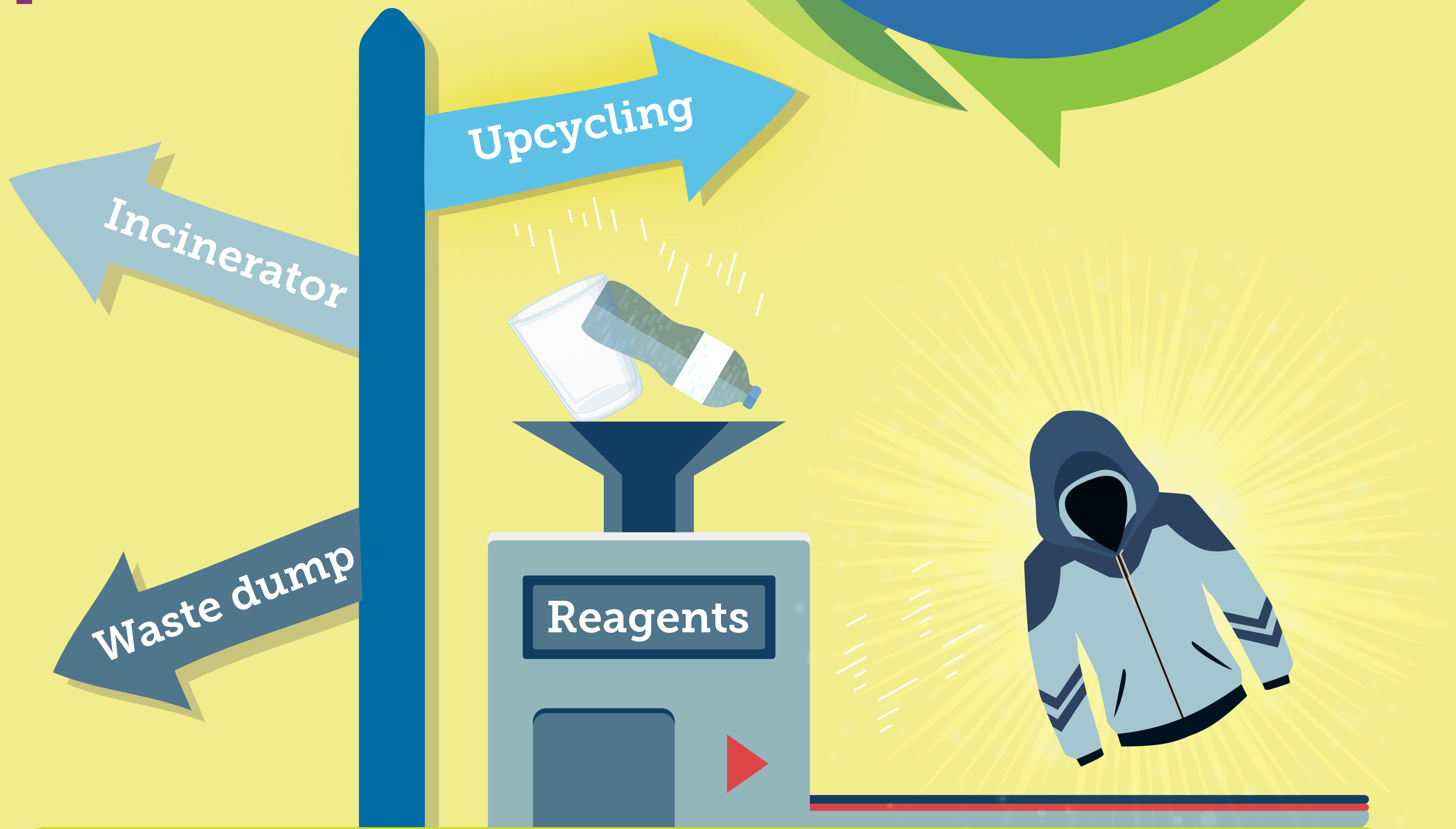

User friendly chemical methods to upcycle plastics to value added products is a potential solution.

Novel method to upcycle polymers: A new life for plastics

Radical-mediated functionalization of post-consumer plastics

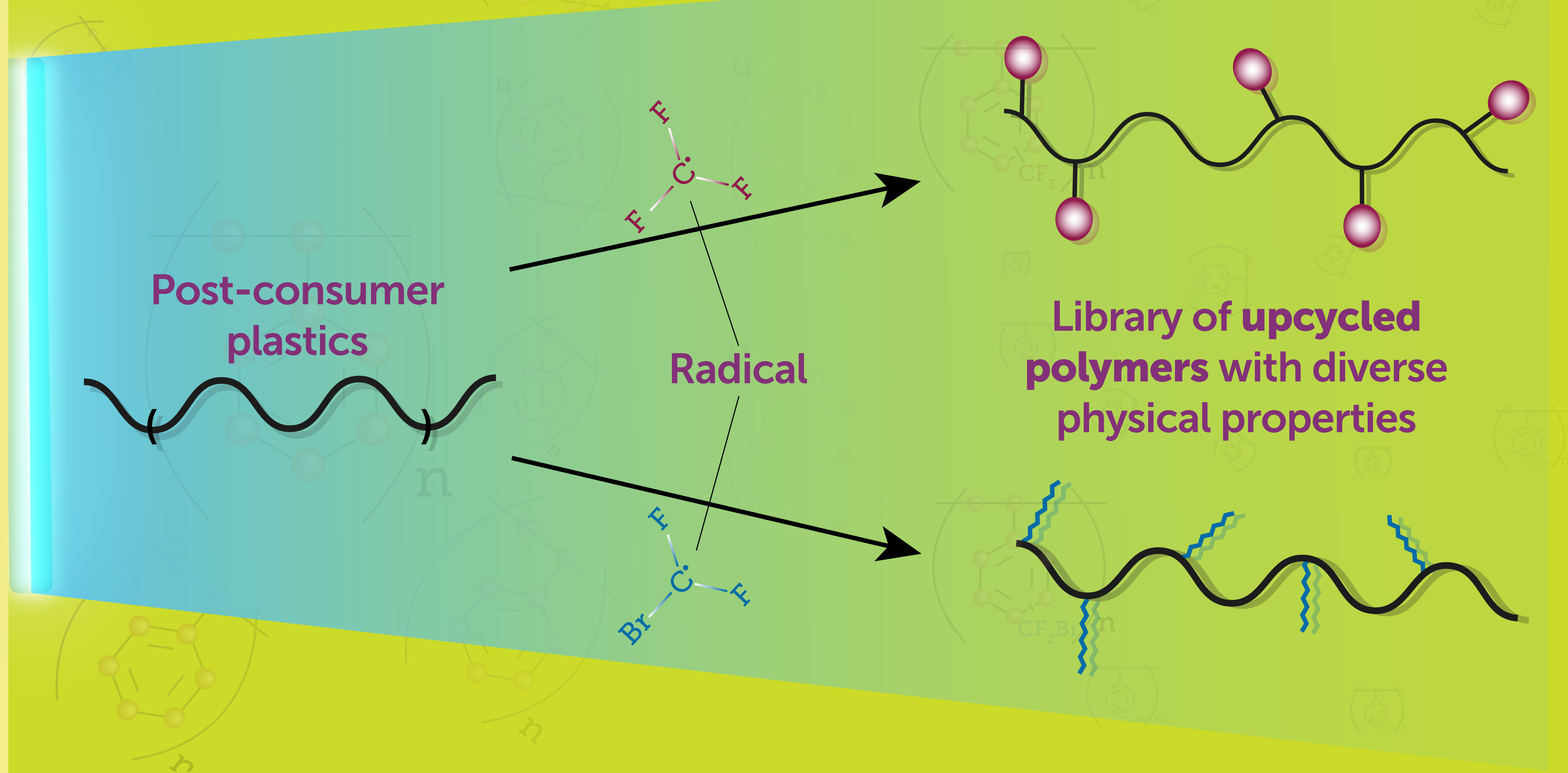

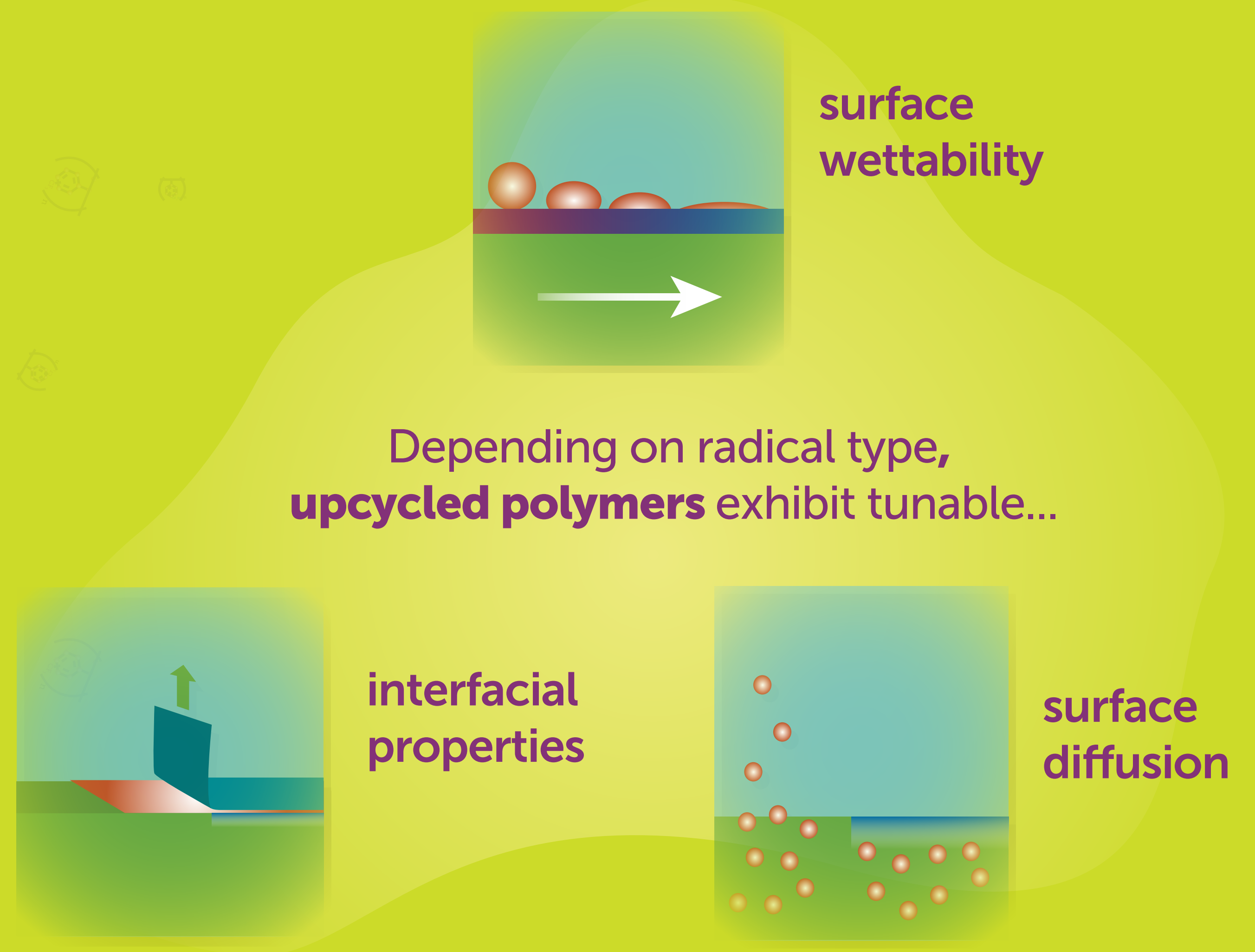

So, how does it add value?

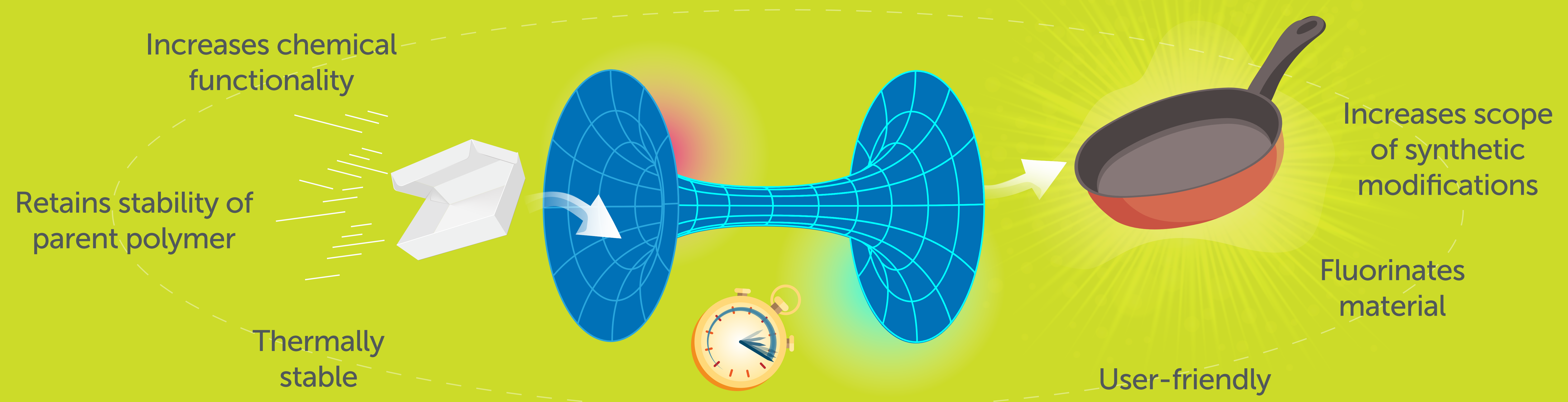

Modification of post-consumer plastics through radical-mediated functionalization results in value-added materials.

Chemical Science
PICK OF THE WEEK
Upcycling aromatic polymers through C–H fluoroalkylation
Leibfarth *et al.* (2019) DOI: 10.1039/C9SC01425J
ROYAL SOCIETY OF CHEMISTRY
