# Supplementary material for: Upcycling aromatic polymers through C–H fluoroalkylation
Source: Chem Sci. 2019 May 29;10(25):6270–7. doi: 10.1039/c9sc01425j (PMC6601422; doi:10.1039/c9sc01425j)
Supplement: Supplementary file 2 [file SC-010-C9SC01425J-s002.pdf]

Electronic Supplementary Material (ESI) for Chemical Science.
This journal is © The Royal Society of Chemistry 2019

Supporting Information

Upcycling Aromatic Polymers through C–H Fluoroalkylation

Sally E. Lewis,[a] Bradley Wilhelmy [a] and Frank A. Leibfarth*,[a]

[a] Department of Chemistry, University of North Carolina at Chapel Hill,125 South Rd, Chapel Hill, NC 27599 (USA)
*E-mail: Frankl@unc.edu

Table of Contents

Materials and Methods 2
Preparation of Polystyrene and Standards 3
Functionalization of Commodity Polymers 6
Further Manipulation of Functionalized Polymers 10
Optimization of trifluoromethylation of 1 13
Regiochemical Determination of Trifluoromethylation 14
Increasing reagent equivalents 15
Gordon-Taylor Theory 16
GPC Data 17
GPC traces of Graft copolymers from bromodifluoromethylated PS 19
Thermal Characterization of poly(styrene-graft-*t*BuA) 21
NMR Characterization 22
References 52

Materials and Methods

General Considerations

NMR spectra were recorded using a Bruker DRX 400 MHz or Bruker AVANCE III 600 MHz CryoProbe spectrometer. Chemical shifts δ (ppm) are referenced to tetramethylsilane (TMS) using the residual solvent as an internal standard (^{1}H and ^{13}C) or using the unified scale relative to the absolute frequency for ^{1}H of 0.1% TMS in $CDCl_3$ (^{19}F) unless otherwise stated. For ^{1}H NMR: $CDCl_3$, 7.26 ppm; DMSO-d6, 2.50 ppm. For ^{13}C NMR: $CDCl_3$, 77.16 ppm. ^{1}H NMR data are reported as follows: chemical shift, multiplicity (s = singlet, d = doublet, t = triplet, q = quartet, m = multiplet, dd = doublet of doublets, bs = broad singlet), coupling constants (Hz), and integration. Infrared (IR) spectra were obtained using PerkinElmer Frontier FT-IR spectrometer. High-resolution mass spectra (HRMS) were obtained with a ThermoScientific Q Exactive HF-X mass spectrometer at the University of North Carolina Mass Spectrometry Core Laboratory.

Macromolecular Characterization

Gel permeation chromatography (GPC) was performed on a Waters 2695 separations module liquid chromatograph equipped with either four Waters Styragel HR columns (WAT044225, WAT044231, WAT044237, and WAT054460) arranged in series or two Agilent Resipore columns (PL1113-6300) maintained at 35 °C, and a Waters 2414 refractive index detector at room temperature. Tetrahydrofuran was used as the mobile phase at a flow rate of 1.0 mL/min. Molecular weight and dispersity data are reported relative to polystyrene standards.

Melting-transition temperature (T_m) and glass-transition temperature (T_g) of precipitated and dried polymer samples were measured using differential scanning calorimetry (DSC) on a TA Instruments Discovery DSC. All T_m and T_g values were obtained from a second heating scan after the thermal history was removed. All heating and cooling rates were 10 °C/min. Decomposition onset temperatures (T_d) and maximum decomposition temperatures (T_{max}) of precipitated and dried polymer samples were measured by thermal gravimetric analysis (TGA) on a TA Instruments Q5000 Thermogravimetric Analyzer. Polymer samples were heated from ambient temperatures to 600 °C at a heating rate of 10 °C/min. Values of T_d (temperature at 10% weight loss) were obtained from wt % vs. temperature (°C) plots.

Static water contact angles were measured from 7 μL droplets of RO water using a Ramé Hart goniometer equipped with DropImageCA software. Reported values are the average of at least three droplets, placed in different regions of the surface.

Preparation of Polystyrene and Standards

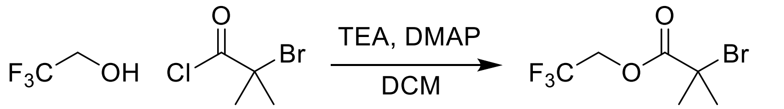

2-trifluoroethyl 2-bromoisobuyrate: 4-dimethylaminopyridine (DMAP) (320 mg, 2.6 mmol), triethylamine (TEA) (8 mL, 57 mmol), and dry dichloromethane (DCM) (60 mL) were added to a flame-dried round bottom under a N_2 atmosphere. The solution was cooled to 0 °C and 2,2,2-trifluoroethanol (19.7 mL, 270 mmol) was added, followed by the drop-wise addition of α-bromoisobutryl bromide (6.5 mL, 53 mmol). The reaction was stirred for 16 hr and purified by washing the DCM with 1 M HCl three times. The DCM layer was then dried with $MgSO_4$, and purified by column chromatography using 5% ether in hexanes as the eluent. The product was isolated as a colorless liquid (7.2 g, 55% yield).
^{1}H NMR (CDCl$_3$, 400 MHz) δ 4.54 (q, J = 8.31 Hz, 2H), 1.95 (s, 6H). **^{19}F NMR (CDCl$_3$, 376 MHz)** δ 73.86 (s). **^{13}C NMR (CDCl$_3$, 101 MHz)** δ 170.41, 122.87 (q, J = 277.3 Hz), 61.50 (q, J = 37.0 Hz), 54.14, 30.58. **IR (neat, ATR, cm^{-1})** 2982, 1754, 1465, 1413, 1375, 1286, 1257, 1144, 1107.

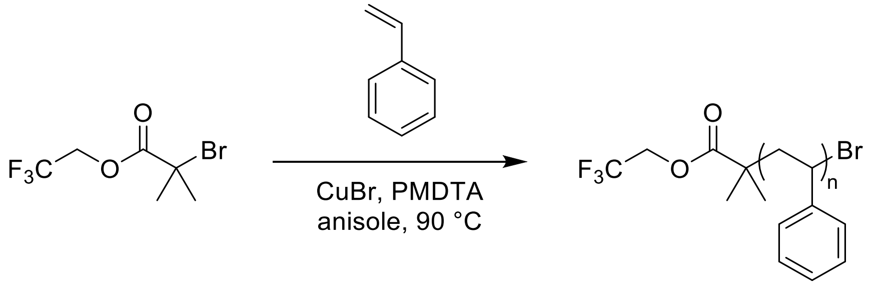

Polystyrene (1): Starting material (140 mg, 0.58 mmol), styrene (6.8 mL, 59 mmol), and *N*,*N*,*N*′,*N*″,*N*″-pentamethyldiethylenetriamine (PMDETA) (0.062 mL, 0.30 mmol) were dissolved in anisole (6.8 mL), freeze-pump-thawed three times and backfilled with N_2. Separately, Cu(I)Br (41 mg, 0.29 mmol) was weighed out under inert conditions in a glovebox. The solution was added to the Cu(I)Br and heated to 90 °C. After 2.5 hr, the reaction was terminated by removing from heat and adding acetone. The solvent was then removed under reduced pressure and precipitated three times into methanol (MeOH) from DCM.
^{1}H NMR (CDCl$_3$, 400 MHz) δ 7.15 (bs), 7.10 (bs), 6.64 (bs), 6.52 (bs), 4.47 (bs), 3.93 (bs), 3.45 (bs), 2.13 (bs), 1.91 (bs), 1.48 (bs), 1.01 (bs). **^{19}F NMR (CDCl$_3$, 376 MHz)** δ -73.82 (s). **^{13}C NMR (CDCl$_3$, 101 MHz)** δ 144.43, 128.09, 127.78, 125.78, 43.99, 40.50. **GPC (THF)** M_n = 6.2 kg/mol, $Đ$ = 1.04. **TGA (°C, 10 °C/min)** T_{deg} = 391. **DSC (°C, 10 °C/min)** T_g = 91.1.

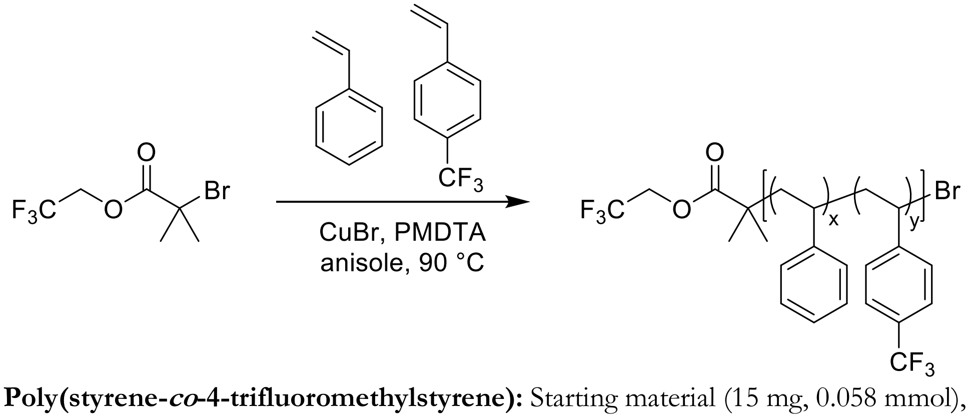

Poly(styrene-*co*-4-trifluoromethylstyrene): Starting material (15 mg, 0.058 mmol), styrene (0.60 mL, 5.2 mmol), 4-trifluoromethyl styrene, (0.086 mL, 0.58 mmol) PMDTA (6.1 µL, 0.030 mmol) were dissolved in anisole (0.68 mL) and freeze-pump-thawed three times and backfilled with N_2. Separately, Cu(I)Br (4.2 mg,

0.030 mmol) was weighed out under inert conditions in a glovebox and taken out in a sealed vial. The solution was added to the Cu(I)Br and heated to 90 °C. After 14.5 hr, the reaction was terminated by removing from heat and adding acetone. The solvent was then removed under reduced pressure and precipitated three times into MeOH from DCM.
^{1}H NMR ($CDCl_3$, 600 MHz) δ 7.14 (bs), 6.63 (bs), 4.57 (bs), 4.48 (bs), 3.94 (bs), 3.65 (bs), 3.56 (bs), 3.46 (bs), 1.91 (bs), 1.49 (bs), 1.02 (bs). **^{19}F NMR ($CDCl_3$, 376 MHz)** δ -62.33 (s), -73.82 (s). **^{13}C NMR ($CDCl_3$, 151 MHz)** δ 149.24, 145.19, 128.08, 127.76, 125.79, 124.95, 123.58, 43.85, 40.52. **GPC (THF)** M_n = 4.0 kg/mol, $Đ$ = 1.14. **TGA (°C, 10 °C/min)** T_{deg} = 384. **DSC (°C, 10 °C/min)** T_g = 80.8.

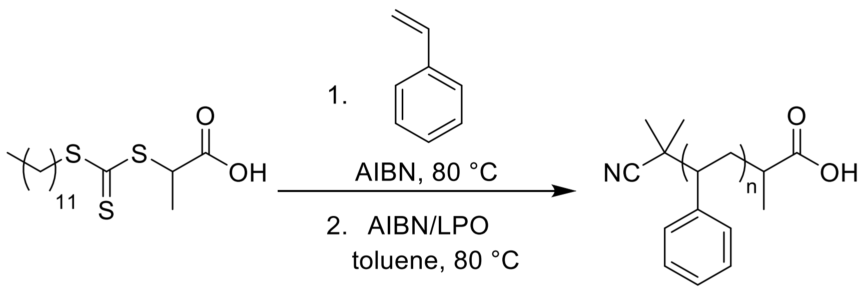

Polystyrene: 2-(Dodecylthiocarbonothioylthio)-2-methylpropanoic acid (77 mg, 0.22 mmol), 2,2'-Azobis(2-methylpropionitrile) (AIBN) (7.0 mg, 0.043 mmol), and styrene (20 mL, 180 mmol) were added to a 100 mL round bottom, equip with a stir bar. The reaction mixture was freeze-pump-thawed three times, backfilled with N_2, and heated to 80 °C. After 6 hr, the reaction was terminated by removing from heat and adding acetone. The solvent was then removed under reduced pressure and precipitated three times into MeOH from DCM. The purified intermediate (1100 mg, 0.12 mmol), AIBN (210 mg, 1.3 mmol) lauroyl peroxide (LPO) (50 mg, 0.12 mmol), and toluene (22 mL) were added to a round bottom, equip with a stir bar, freeze-pump-thawed three times, backfilled with N_2, and put in an 80 °C oil bath for 4 hours. The solvent was then removed under reduced pressure and precipitated three times into MeOH from DCM.
^{1}H NMR ($CDCl_3$, 600 MHz) δ 7.13 (bs), 7.08 (bs), 6.61 (bs), 6.43 (bs), 6.50 (bs), 2.10 (bs), 1.88 (bs), 1.47 (bs). **^{13}C NMR ($CDCl_3$, 151 MHz)** δ 145.40, 128.08, 127.76, 125.77, 125.61, 43.89, 40.44. **GPC (THF)** M_n = 11 kg/mol, $Đ$ = 1.22. **TGA (°C, 10 °C/min)** T_{deg} = 373. **DSC (°C, 10 °C/min)** T_g = 98.9.

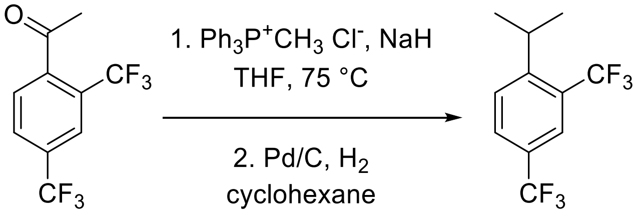

2,4-bis(trifluoromethyl)cumene: Methyl triphenylphosphine chloride (2.40 g, 7.68 mmol) and NaH (204 mg, 0.8.52 mmol) were dissolved in dry THF (21.3 mL) in the glovebox. The solution was taken out of the glovebox in a sealed vial and refluxed for 2 hours followed by the addition of 2,4 bistrifluoromethyl acetophenone (1 mL, 5.32 mmol). The reaction solution was refluxed overnight, poured into water, and extracted with DCM three times. The product was purified by column chromatography (with hexanes as the eluent), the solvent was removed under reduced pressure and all of it was submitted to the second reaction. In a vial the product from the first reaction, 10 wt % loading Pd/C (168 mg, 200 mmol), and dry cyclohexane (3.42 mL) were put under hydrogen at atmospheric pressure. After stirring for 16 hours, the solution was directly filtered through celite, washed 3 times with 5 mL of hexanes, and the solvent was removed under reduced pressure to afford a colorless liquid (190 mg, 52.4% yield).
^{1}H NMR ($CDCl_3$, 400 MHz) δ 7.87 (s, 1H), 7.77 (d, J = 8.32 Hz, 1H), 7.63 (d, J = 8.28 Hz, 1H), 3.43 (m, J = 6.68 Hz, 1H), 1.30 (d, J = 6.80 Hz, 6H). **^{19}F NMR ($CDCl_3$, 376 MHz)** δ -59.65, -62.86. **^{13}C NMR ($CDCl_3$, 151 MHz)** δ 152.63, 128.89, 128.39 (q, J = 31.22 Hz), 128.52 (q, J = 31.80 Hz), 128.33, 124.05 (q, J

= 273.98 Hz), 123.77 (q, *J* = 271.91 Hz), 123.02 (m), 29.65, 24.12. **IR (neat, ATR, cm^{-1})** 2964, 2879, 1630, 1588, 1468, 1345, 1305, 1273, 1266, 1191, 1179, 1118, 1048, 1049.

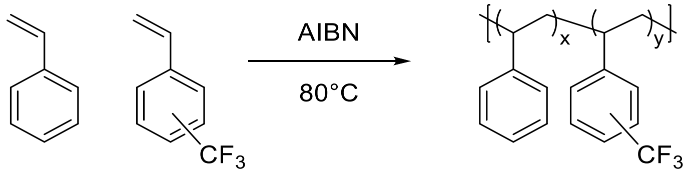

General Procedure 1 for the free radical polymerization of trifluoromethylstyrene derivatives: Styrene, trifluoromethylated styrene, and AIBN were dissolved in anisole. The solution was freeze-pump-thawed three times and back filled with N_2, and heated to 70 °C. After 12 hr, the solution was cooled, the solvent removed under reduced pressure, and the polymer purified by precipitation into MeOH from DCM.

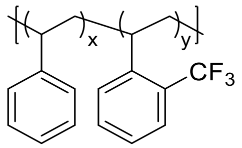

Poly(styrene-*co*-2-trifluoromethylstyrene): A 2-dram reaction vial was equipped with a stir bar and charged with styrene (0.60 mL, 5.2 mmol), 2-trifluoromethyl styrene (0.085 mL, 0.58 mmol), AIBN (10 mg, 0.061 mmol), and anisole (0.60 mL). The reaction was carried out according to general procedure 1.
^{1}H NMR ($CDCl_3$, 400 MHz) δ 7.49 (bs), 7.09 (bs),, 6.62 (bs), 6.55 (bs), 2.77 (bs), 1.89 (bs), 1.50 (bs), 0.99 (bs). **^{19}F NMR ($CDCl_3$, 376 MHz)** δ –58.41. **^{13}C NMR ($CDCl_3$, 101 MHz)** δ 145.31, 131.65, 128.08, 127.76, 125.77, 43.87, 40.50. **GPC (THF)** M_n = 23 kg/mol, $Đ$ = 1.68. **TGA (°C, 10 °C/min)** T_{deg} = 380. **DSC (°C, 10 °C/min)** T_g = 107.

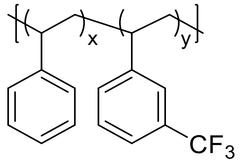

Poly(styrene-*co*-3-trifluoromethylstyrene): A 2-dram reaction vial was equipped with a stir bar and charged with styrene (0.60 mL, 5.2 mmol), 3-trifluoromethyl styrene (0.086 mL, 0.58 mmol), AIBN (10 mg, 0.061 mol), and anisole (0.60 mL). The reaction was carried out according to general procedure 1.
^{1}H NMR ($CDCl_3$, 400 MHz) δ 7.33 (bs), 7.08 (bs), 6.61 (bs), 6.50 (bs), 2.09 (bs), 1.86 (bs), 1.46 (bs), 1.16 (bs), 0.97 (bs). **^{19}F NMR ($CDCl_3$, 376 MHz)** δ –61.49 (bs). **^{13}C NMR ($CDCl_3$, 101 MHz)** δ 145.33, 128.17, 127.79, 125.79, 43.71, 40.51. **GPC (THF)** M_n = 30 kg/mol, $Đ$ = 1.72. **TGA (°C, 10 °C/min)** T_{deg} = 376. **DSC (°C, 10 °C/min)** T_g = 95.4.

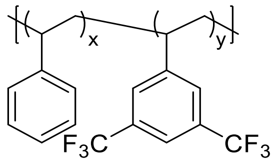

Poly(styrene-*co*-2,4-bistrifluoromethylstyrene): A 2-dram reaction vial was equipped with a stir bar and charged with styrene (0.60 mL, 5.2 mmol), 2,4-bistrifluoromethyl styrene (0.10 mL, 0.58 mmol), AIBN (10 mg, 0.061 mmol), and anisole (0.60 mL). The reaction was carried out according to general procedure 1.
^{1}H NMR ($CDCl_3$, 600 MHz) δ 7.57 (bs), 7.08 (bs), 6.62 (bs), 6.50 (bs), 1.88 (bs), 1.48 (bs), 1.18 (bs), 0.98 (bs). **^{19}F NMR ($CDCl_3$, 376 MHz)** δ –62.47 (bs). **^{13}C NMR ($CDCl_3$, 151 MHz)** δ 147.70, 145.42, 130.90, 128.08, 127.76, 127.54, 125.78, 124.31, 122.53, 119.85, 41.93, 40.45. **GPC (THF)** M_n = 31 kg/mol, $Đ$ = 1.69. **TGA (°C, 10 °C/min)** T_{deg} = 379. **DSC (°C, 10 °C/min)** T_g = 99.6.

Functionalization of Commodity Polymers

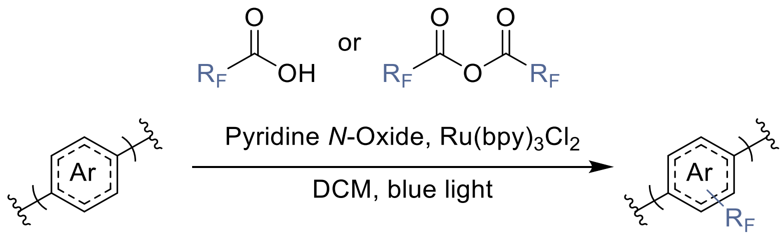

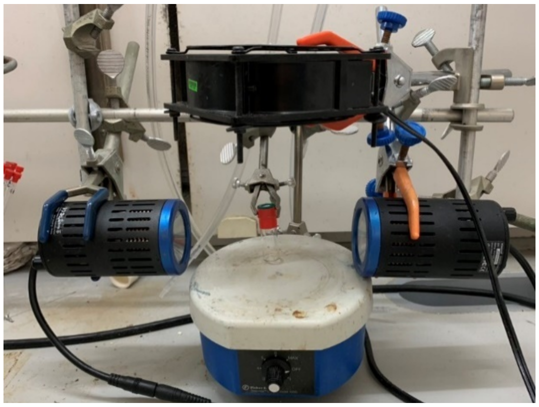

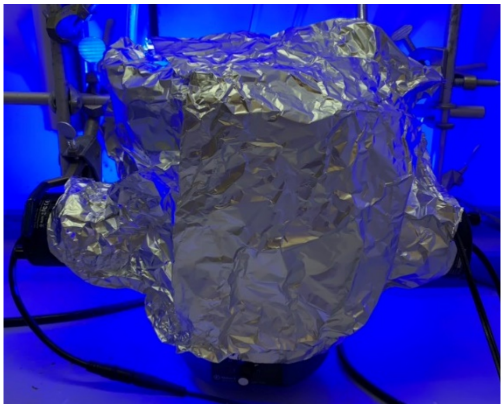

General procedure 2 for perfluoroalkylation of polyaromatics:
The polyaromatic starting material, pyridine *N*-oxide, $Ru(bpy)_3Cl_2$, and DCM were added to a 1 dram vial, equip with a stir bar. The vial cap was wrapped with electrical tape, exposed to two Kessil-brand "Tuna Blue" aquarium lights (420 nm) each 6.5 cm away from the vial, cooled with an AC Infinity Axial 1238 fan, and the whole system was wrapped in aluminum foil for 6-16hr. After the reaction was complete, the solvent was removed under reduced pressure and precipitated in MeOH from DCM to yield the functionalized product as a solid white product. ^{1}H NMR, ^{19}F NMR, ^{13}C NMR, GPC, TGA, and DSC were taken of all samples.

Determination of mol% functionalization of 1: Upon purification, the percent trifluoromethylation of the commodity polymers can be determined through integration of the ^{19}F NMR. The chain-end peak (-73.8 ppm) was used as an internal standard and was set to an integration of 1, the rest of the polymeric peaks were integrated to determine mol% functionalization.

Determination of mol% functionalization of commodity polymers: Upon purification, the percent trifluoromethylation of the commodity polymers can be determined through integration of the ^{19}F NMR. Fluorobenzene (-113.15 ppm) was used as an internal standard and was set to an integration of 1, the rest of the polymeric peaks were integrated to determine mol% functionalization.

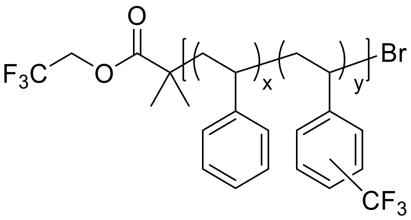

Trifluoromethylated polystyrene: 1 (83 mg, 0.80 mmol of repeat unit), pyridine *N*-oxide (76 mg, 0.80 mmol), TFAA (0.12 mL, 0.88 mmol), $Ru(bpy_3)Cl_2$ (6.0 mg, 0.0080 mmol) and DCM (2mL) were reacted according to general procedure 2. The reaction yielded 31.8 % trifluoromethylation. Similar characterization

data was obtained using other stoichiometric ratios of reagents to repeat unit. See accompanying tables and figures for more information.
^{1}H NMR ($CDCl_3$, 400 MHz) δ 7.07 (bs), 6.57 (bs), 4.42 (bs), 3.91 (bs), 3.54 (bs), 1.82 (bs), 1.43 (bs), 0.97 (bs). **^{19}F NMR ($CDCl_3$, 376 MHz)** δ -58.48 (bs), -59.43 (bs), -62.52 (bs), -73.82 (bs), -57.39 (bs). **^{13}C NMR ($CDCl_3$, 101 MHz)** δ 145.18, 130.99, 128.19, 127.65, 125.90, 125.02, 124.25, 122.90, 43.98, 40.62. **GPC (THF)** M_n = 7.5 kg/mol, $Đ$ = 1.04. **TGA (°C, 10 °C/min)** T_{deg} = 348. **DSC (°C, 10 °C/min)** T_g = 69.5.

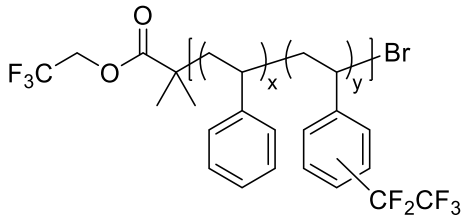

Perfluoroethylated polystyrene: 1 (83 mg, 0.80 mmol of repeat unit), pyridine *N*-oxide (76 mg, 0.80 mmol), pentafluorobutyric anhydride (0.17 mL, 0.88 mmol), Ru(bpy_3)Cl_2 (6.0 mg, 0.008 mmol) and DCM (2mL) were reacted according to general procedure 2. The reaction yielded 44.6 mol% functionalization.
^{1}H NMR ($CDCl_3$, 600 MHz) δ 7.05 (bs), 6.52 (bs), 4.41 (bs), 3.88 (bs), 3.50 (bs), 1.81 (bs), 1.42 (bs), 0.95 (bs). **^{19}F NMR ($CDCl_3$, 376 MHz)** δ -73.82 (bs), -82.81 (bs), -84.89 (bs), -114.88 (bs). **^{13}C NMR ($CDCl_3$, 151 MHz)** δ 149.12, 145.08, 131.03, 128.19, 127.54, 126.12, 124.13, 120.17 (m), 118.26 (m), 116.41 (m), 115.25 (m), 113.37 (m), 111.72 (m), 43.84, 40.58. **GPC (THF)** M_n = 6.8 kg/mol, $Đ$ = 1.05. **TGA (°C, 10 °C/min)** T_{deg} = 365. **DSC (°C, 10 °C/min)** T_g =72.7

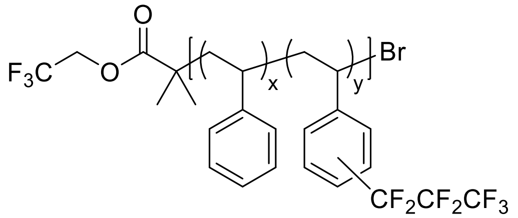

Perfluoropropylated polystyrene: 1 (83 mg, 0.80 mmol of repeat unit), pyridine *N*-oxide (76 mg, 0.80 mmol), heptafluoropropionic anhydride (0.12 mL, 0.88 mmol), Ru(bpy_3)Cl_2 (6.0 mg, 0.0080 mmol) and DCM (2mL) were reacted according to general procedure 2. The reaction yielded 37.2 mol% functionalization.
^{1}H NMR ($CDCl_3$, 400 MHz) δ 7.06, 6.55, 4.41, 3.90, 3.54, 1.81, 1.43, 1.03, 0.97. **^{19}F NMR ($CDCl_3$, 376 MHz)** δ -73.82, -80.14, -80.73, -84.87, -103.96, -111.77, -114.90, -119.28, -124.65, -126.62. **^{13}C NMR ($CDCl_3$, 151 MHz)** δ 149.44, 145.16, 131.28, 128.19, 127.59, 126.41, 125.95, 124.41, 121.01 (m), 119.12 (m), 117.21 (m), 115.30 (m), 113.62 (m), 110.44 (m), 108.69 (m), 106.91 (m), 45.77, 43.92, 41.68, 40.57. **GPC (THF)** M_n = 7.1 kg/mol, $Đ$ = 1.04. **TGA (°C, 10 °C/min)** T_{deg} = 297. **DSC (°C, 10 °C/min)** T_g = 71.2

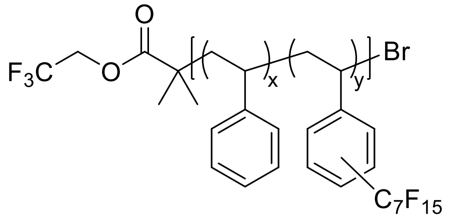

Perfluoroheptylated polystyrene: 1 (83 mg, 0.80 mmol of repeat unit), pyridine *N*-oxide (76 mg, 0.80 mmol), pentadecafluorooctonoyl chloride (0.22 mL, 0.88 mmol), Ru(bpy_3)Cl_2 (6.0 mg, 0.0080 mmol) and $CHCl_3$ (2 mL) and MeCN (2 mL) were reacted according to general procedure 2. The reaction yielded 16.1 mol% functionalization.
^{1}H NMR ($CDCl_3$, 600 MHz) δ 7.06 (bs), 6.57 (bs), 4.42 (bs), 3.89 (bs), 3.50 (bs), 1.84 (bs), 1.43 (bs), 0.97 (bs). **^{19}F NMR ($CDCl_3$, 376 MHz)** δ -71.90 (bs), -73.78 (bs), -80.88 (bs), -110.65 (bs), -120.42 (bs), -121.53

(bs), -122.09 (bs), -122.81 (bs), -126.22 (bs). **^{13}C NMR (CDCl$_3$, 151 MHz)** δ 145.25, 128.15, 127.66, 126.52, 124.47, 120.17 (m), 118.26 (m), 116.35 (m), 110.64 (m), 108.67 (m), 106.64 (m), 43.93, 40.37. **GPC (THF)** M_n = 9.0 kg/mol, $Đ$ = 1.04. **TGA (°C, 10 °C/min)** T_{deg} = 337. **DSC (°C, 10 °C/min)** T_g = 67.8

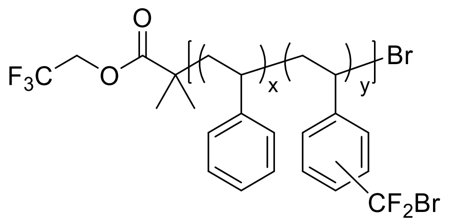

Bromodifluoromethylated polystyrene: 1 (83 mg, 0.80 mmol of repeat unit), pyridine *N*-oxide (76 mg, 0.8 mmol), bromodifluoroacetic anhydride (0.12 mL, 0.88 mmol), Ru(bpy$_3$)Cl$_2$ (6.0 mg, 0.0080 mmol) and DCM (2mL) were reacted according to general procedure 2. The reaction yielded 15 mol% functionalization.
^{1}H NMR (CDCl$_3$, 400 MHz) δ 7.11 (bs), 6.59 (bs), 4.42 (bs), 3.90 (bs), 3.55 (bs),1.84 (bs), 1.04 (bs), 0.97 (bs). **^{19}F NMR (CDCl$_3$, 376 MHz)** δ -42.26 (bs), -43.36 (bs), -61.60 (bs), -73.82 (bs). **^{13}C NMR (CDCl$_3$, 151 MHz)** δ 145.25, 137.85, 135.65, 130.62, 128.19, 127.68, 125.90, 124.17, 121.79, 118.64 (t, *J* = 303.5 Hz), 45.80, 42.80, 41.66, 40.57. **GPC (THF)** M_n = 4.6 kg/mol, $Đ$ = 1.11. **TGA (°C, 10 °C/min)** T_{deg} = 292. **DSC (°C, 10 °C/min)** T_g =80.5

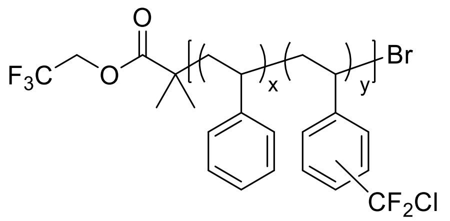

Chlorodifluoromethylated polystyrene: 1 (83 mg, 0.8 mmol of repeat unit), pyridine *N*-oxide (76 mg, 0.8 mmol), chlorodifluoroacetic anhydride (0.15 mL, 0.88 mmol), Ru(bpy$_3$)Cl$_2$ (6.0 mg, 0.0080 mmol) and DCM (2mL) were reacted according to general procedure 2. The reaction yielded 26 mol% functionalization.
^{1}H NMR (CDCl$_3$, 400 MHz) δ 7.11 (bs), 6.60 (bs), 4.43 (bs), 3.92 (bs), 3.55 (bs), 3.44 (bs), 1.86 (bs), 1.45 (bs), 1.06 (bs), 0.98 (bs). **^{19}F NMR (CDCl$_3$, 376 MHz)** δ -44.82 (bs), -47.66 (bs), -48.51 (bs), 63.98 (bs), -73.82 (bs). **^{13}C NMR (CDCl$_3$, 151 MHz)** δ 148.84, 145.17, 136.05, 133.85, 130.61, 128.20, 127.64, 125.93, 124.48, 122.20, 45.82, 43.95, 41.66, 40.59. **GPC (THF)** M_n = 3.9 kg/mol, $Đ$ = 1.05. **TGA (°C, 10 °C/min)** T_{deg} = 306. **DSC (°C, 10 °C/min)** T_g = 84.8

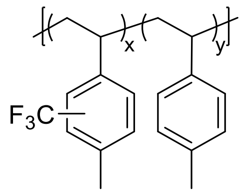

Trifluoromethylated poly(4-methylstyrene): Poly(4-methylstyrene) (95 mg, 0.80 mmol of repeat unit), pyridine *N*-oxide (150 mg, 1.6 mmol), TFAA (0.24 mL, 1.8 mmol), Ru(bpy)$_3$Cl$_2$ (12 mg, 0.016 mmol) and DCM (2mL) were reacted according to general procedure 2. The polymer was precipitated into hexanes from DCM for purification. The reaction yielded 62 mol% functionalization.
^{1}H NMR (CDCl$_3$, 400 MHz) δ 6.85 (bs), 6.45 (bs), 5.22 (bs), 4.58 (bs), 2.27 (bs), 1.39 (bs). **^{19}F NMR (CDCl$_3$, 376 MHz)** δ -58.48, -60.94, -61.75, -75.47. **^{13}C NMR (CDCl$_3$, 101 MHz)** δ142.23, 134.63, 131.75, 130.52, 128.73, 127.46, 125.13, 123.30, 43.68, 40.15, 25.52, 21.04, 18.87. **TGA (°C, 10 °C/min)** T_{deg} = 295. **DSC (°C, 10 °C/min)** T_g = 103. Note: due to low refractive index signal of trifluoromethylated poly(4-methylstyrene) GPC of the product was inconclusive.

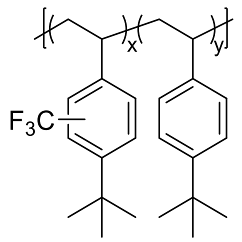

Trifluoromethylated poly(4-*tert*-butylstyrene): poly(4-*tert*-butylstyrene) (130 mg, 0.80 mmol of repeat unit), pyridine *N*-oxide (150 mg, 1.6 mmol), TFAA (0.24 mL, 1.8 mmol), $Ru(bpy)_3Cl_2$ (12 mg, 0.016 mmol) and DCM (2mL) were reacted according to general procedure 2. The reaction yielded 23 mol% functionalization.

^{1}H NMR ($CDCl_3$, 600 MHz) δ 7.08 (bs), 6.53 (bs), 6.36 (bs), 1.73 (bs), 1.26 (bs). **^{19}F NMR ($CDCl_3$, 376 MHz)** δ -52.68 (bs), -58.28 (bs), -74.98 (bs). **^{13}C NMR ($CDCl_3$, 151 MHz)** δ 148.22, 142.71, 127.36, 124.76, 43.37, 39.88, 36.22, 34.36, 32.24, 31.61. **GPC (THF)** M_n = 20 kg/mol, $Đ$ = 1.88. **TGA (°C, 10 °C/min)** T_{deg} = 356. **DSC (°C, 10 °C/min)** T_g =146.

Trifluoromethylated Tritan copolyester®: Tritan copolyester® (180 mg, 0.80 mmol of aryl unit), pyridine *N*-oxide (150 mg, 1.6 mmol), TFAA (0.24 mL, 1.8 mmol), $Ru(bpy)_3Cl_2$ (12 mg, 0.016 mmol), DCM (2mL) and TFA (0.80 mL) were reacted according to general procedure 2. The reaction yielded 20 mol% functionalization. GPC was not taken due to solubility restraints.

^{1}H NMR ($CDCl_3$, 600 MHz) δ 9.13 (bs), 8.71 (bs), 8.37 (bs), 8.32 (bs), 8.26 (bs), 8.11 (bs), 8.09 (bs), 7.81 (bs), 7.33 (bs), 7.08 (bs), 4.77 (bs), 4.62 (bs), 4.28 (bs), 4.18 (bs), 3.99 (bs), 3.64 (bs), 3.33 (bs), 2.06 (bs), 1.93 (bs), 1.89 (bs), 1.80 (bs), 1.65 (bs), 1.56 (bs), 1.42 (bs), 1.29 (bs), 1.26 (bs), 1.15 (bs), 1.11 (bs). **^{19}F NMR ($CDCl_3$, 376 MHz)** δ -59.49, -59.62, -59.73, -75.01, -75.31, -75.41. **^{13}C NMR ($CDCl_3$, 151 MHz)** δ 166.29, 165.81, 165.43, 164.52, 164.15, 163.15, 163.48, 162.45, 160.66, 160.43, 146.20, 141.91, 139.47, 135.14, 134.34, 134.12, 133.68, 132.71, 130.53, 130.37, 129.53, 127.83, 126.86, 126.26, 125.64, 123.83, 122.01, 81.58, 81.43, 80.62, 71.36, 70.58, 70.17, 68.00, 41.88, 40.53, 37.15, 34.55, 28.91, 25.37, 22.83, 16.99. **TGA (°C, 10 °C/min)** T_{deg} = 355. **DSC (°C, 10 °C/min)** T_g = 98.8.

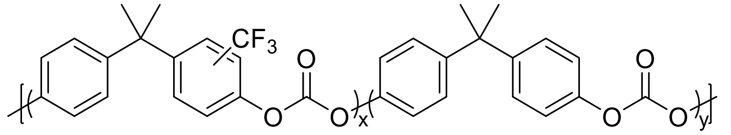

Trifluoromethylated poly(bisphenol A carbonate): Poly(bisphenol A carbonate) (100 mg, 0.80 mmol of aryl unit), pyridine *N*-oxide (150 mg, 1.6 mmol), TFAA (0.24 mL, 1.8 mmol), $Ru(bpy)_3Cl_2$ (12 mg, 0.016 mmol) and DCM (2mL) were reacted according to general procedure 2. The reaction yielded 20 mol% functionalization.

^{1}H NMR ($CDCl_3$, 600 MHz) δ 7.60 (bs), 7.44 (bs), 7.43 (bs), 7.29 (bs), 7.27 (bs),7.20 (bs),7.19 (bs), 1.73 (bs), 1.70 (bs). **^{19}F NMR ($CDCl_3$, 376 MHz)** δ -54.55 (bs), -60.78 (bs), -61.14 (bs), -61.42 (bs), -62.45 (bs). **^{13}C NMR ($CDCl_3$, 151 MHz)** δ 152.18, 152.13, 152.63, 121.57, 149.19, 149.17, 148.93, 148.52, 148.30, 147.21, 146.08, 132.09, 127.95, 124.89, 123.79, 123.72, 123.40, 123.37, 123.24, 122.52, 122.10, 121.98, 121.89, 120.64, 120.52, 120.35, 120.23, 43.00, 42.80, 42.59, 30.95, 30.84. **GPC (THF)** M_n = 17 kg/mol, $Đ$ = 1.47. **TGA (°C, 10 °C/min)** T_{deg} = 306. **DSC (°C, 10 °C/min)** T_g = 84.8.

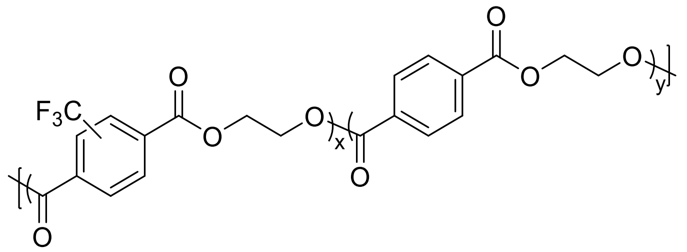

Trifluoromethylated poly(ethylene terephthalate): Poly(ethylene terephthalate) (150 mg, 0.80 mmol of repeat unit), pyridine *N*-oxide (150 mg, 1.6 mmol), TFAA (0.24 mL, 1.8 mmol), $Ru(bpy)_3Cl_2$ (12 mg, 0.016 mmol) and DCM (2mL) and TFA (0.60 mL) were reacted according to general procedure 2. The reaction yielded 14 mol% functionalization. GPC was not taken due to solubility restraints.
^{1}H NMR ($CDCl_3$, 600 MHz) δ 9.18 (bs), 8.72 (bs), 8.37 (bs), 8.24 (bs), 8.08 (bs), 7.82 (bs), 4.68 (bs), 4.42 (bs), 2.65 (bs), 1.96 (bs), 1.25 (bs). **^{19}F NMR ($CDCl_3$, 376 MHz)** δ -59.50, -59.57, -59.78. **^{13}C NMR ($CDCl_3$, 151 MHz)** δ 165.85, 134.12, 132.86, 130.54, 129.67, 127.97, 66.06, 65.01, 63.17, 29.86, 25.59. T_{deg} =365. **DSC (°C, 10 °C/min)** T_g = 64.0

Further Manipulation of Functionalized Polymers

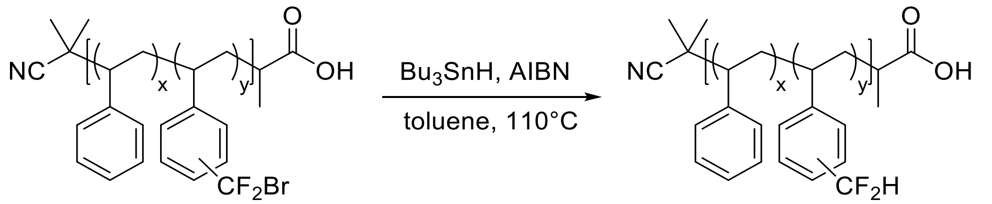

Difluoromethylated polystyrene: Starting material (50 mg, 0.097 mmol of CF_2Br), Bu_3SnH (0.26 mL, 0.97 mmol), AIBN (16 mg, 0.097 mmol), and dry toluene (2 mL) were freeze-pump-thawed three times, backfilled with N_2 and heated at 110 °C overnight. The product was purified by precipitating from toluene into MeOH. 98% conversion from CF_2Br to CF_2H was seen by NMR.
^{1}H NMR ($CDCl_3$, 400 MHz) δ 7.08 (bs), 6.58 (bs), 1.82 (bs), 1.42 (bs), 0.94 (bs). **^{19}F NMR ($CDCl_3$, 376 MHz)** δ -95.40, -109.69, -110.14. **^{13}C NMR ($CDCl_3$, 151 MHz)** δ 145.24, 134.06, 131.88, 130.07, 128.19, 128.06, 127.67, 125.85, 12538, 123.05, 114.98 (t, J = 238.5 Hz), 43.95, 40.57. **GPC (THF)** M_n = 19 kg/mol, $Đ$ = 1.31. **TGA (°C, 10 °C/min)** T_{deg} = 322. **DSC (°C, 10 °C/min)** T_g = 96.6

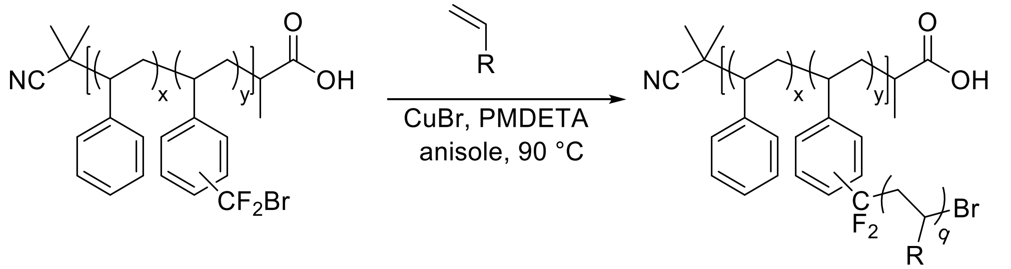

General procedure 3 for graft copolymer synthesis: Starting material, monomer, CuBr, PMDETA, and anisole were freeze-pump-thawed three times, backfilled with N_2 and heated to 90 °C. The polymerization was monitored by ^{1}H NMR and GPC and went for 0.5-30 hr. After the reaction was completed, the polymer was purified by precipitation into cold *iso*-propanol from DCM. See accompanying figures for GPC and thermal data plots.

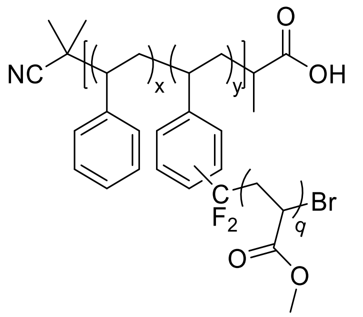

Poly(styrene-*graft*-methyl acrylate): Starting material (100 mg of polymer, 0.0749 mmol of CF_2Br), methyl acrylate (0.32 mL, 3.74 mmol), CuBr (5.37 mg, 0.0374 mmol), PMDETA (0.0156 mL, 0.0749 mmol), and anisole (3 mL) were reacted according to the general procedure 3.
^{1}H NMR ($CDCl_3$, 600 MHz) δ 7.05 (bs), 6.57 (bs), 4.26 (bs), 3.78 (bs), 3.67 (bs), 2.69 (bs), 2.33 (bs), 1.96 (bs), 1.71 (bs), 1.43 (bs). **^{13}C NMR ($CDCl_3$, 151 MHz)** δ 174.91, 174.30, 145.18, 128.01, 127.62, 125.67, 124.39, 121.86, 53.15, 53.13, 52.15, 52.10, 51.83, 51.80, 51.79, 43.81, 43.50, 42.14, 41.85, 41.28, 40.37, 37.67, 37.09, 34.97. **GPC (THF)** M_n = 16 kg/mol, $Đ$ = 1.35. **TGA (°C, 10 °C/min)** T_{deg} = 337. **DSC (°C, 10 °C/min)** T_g = 35.2

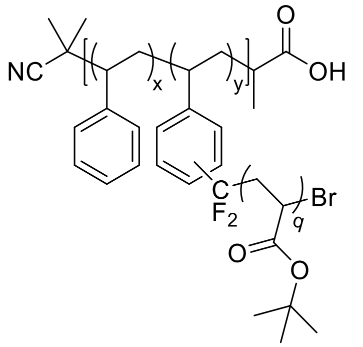

Poly(styrene-*graft*-*tert*-butyl acrylate): Starting material (100 mg of polymer, 0.0749 mmol of CF_2Br), *tert*-butyl acrylate (0.55 mL, 3.74 mmol), CuBr (5.37 mg, 0.0374 mmol), PMDETA (0.0156 mL, 0.0749 mmol), and anisole (3 mL) were reacted according to the general procedure 3.
^{1}H NMR ($CDCl_3$, 400 MHz) δ 7.04 (bs), 6.57 (bs), 4.12 (bs), 2.60 (bs), 2.26 (bs), 1.84 (bs), 1.45 (bs). **^{13}C NMR ($CDCl_3$, 151 MHz)** δ 174.08, 145.30, 128.12, 125.78, 82.53, 81.26, 64.61, 46.15, 43.95, 42.45, 31.12, 28.24, 28.15, 27.84, 25.52. **GPC (THF)** M_n = 16 kg/mol, $Đ$ = 1.21. **TGA (°C, 10 °C/min)** T_{deg} = 188. **DSC (°C, 10 °C/min)** T_g = 75.2

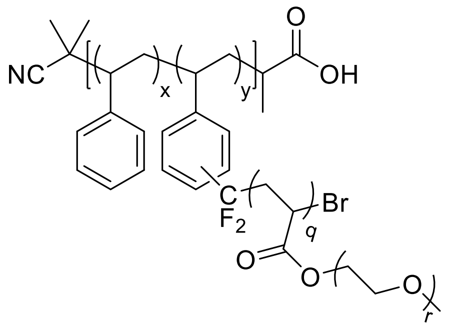

Poly(styrene-*graft*-poly(ethylene glycol) acrylate): Starting material (100 mg of polymer, 0.0749 mmol of CF_2Br), poly(ethylene gycol) acrylate (1.6 mL, 3.74 mmol), CuBr (5.37 mg, 0.0374 mmol), PMDETA (0.0156 mL, 0.0749 mmol), and anisole (3 mL) were reacted according to the general procedure 3.

^{1}H NMR (CDCl$_3$, 600 MHz)** δ 7.06 (bs), 6.56 (bs), 6.44 (bs), 4.16 (bs), 3.66 (bs), 3.63 (bs), 3.55 (bs), 3.37 (bs), 2.30 (bs), 1.81 (bs), 1.40 (bs). **^{13}C NMR (CDCl$_3$, 151 MHz)** δ 174.30, 145.38, 128.05, 127.73, 125.74, 125.58, 72.03, 70.66, 70.46, 68.89, 65.12, 63.47, 59.17, 41.31, 40.41. **GPC (THF)** M_n = 19 kg/mol, Đ = 1.18. **TGA (°C, 10 °C/min)** T_{deg} = 321. **DSC (°C, 10 °C/min)** T_g = -129, -91.5.

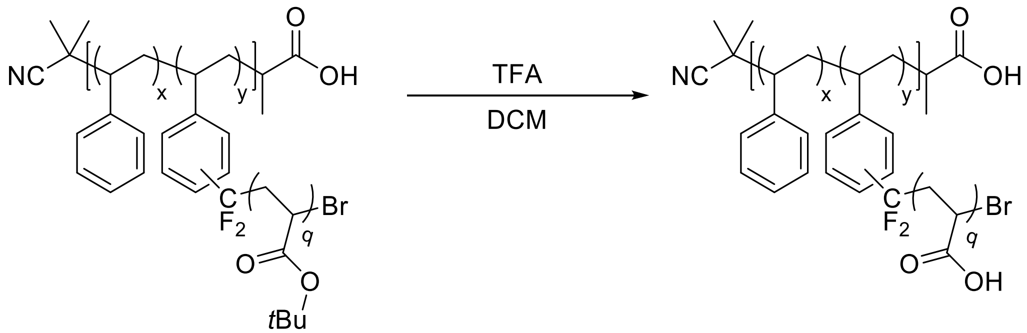

poly(styrene-*graft*-acrylic acid): Starting material (100 mg, 0.22 mmol of *tert*-butyl acrylate), was dissolved in DCM (5.4 mL) and trifluoroacetic acid (TFA) (5.4 mL, 71 mmol) was added to the solution and was stirred at room temperature for 16 hr. The solvent was removed and the polymer was purified by precipitation from THF into hexanes.

^{1}H NMR (DMSO, 400 MHz) δ 12.37 (bs), 7.61 (bs), 7.04 (bs), 6.54 (bs), 4.96 (bs), 4.26 (bs), 3.53 (bs), 2.24 (bs), 1.80 (bs), 1.52 (bs), 0.86 (bs). **^{13}C NMR (DMSO, 151 MHz)** δ 175.85, 127.96, 127.20, 125.61, 62.06, 35.84, 34.39, 30.77, 25.55. **GPC (THF)** M_n = 12 kg/mol, Đ = 1.57. **TGA (°C, 10 °C/min)** T_{deg} = 235. **DSC (°C, 10 °C/min)** T_g = 20.8, 97.1

Optimization of Trifluoromethylation of 1

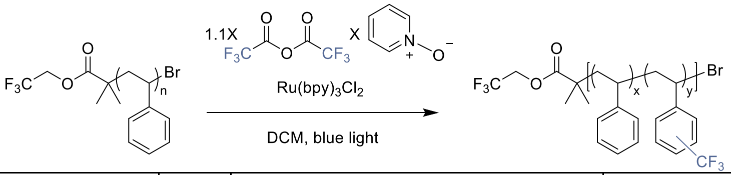

catalyst (mol%)	Time (hr)	Concentration of repeat unit	X	N-oxide	solvent	Average CF_3 per 100 repeat units
**0**	16	0.40	1	Pyridine *N*-oxide	DCM	7
**1**	16	0.40	1	Pyridine *N*-oxide	DCM	37
**0.17**	16	0.40	1	Pyridine *N*-oxide	DCM	33
**0.25**	16	0.40	1	Pyridine *N*-oxide	DCM	29
**0.5**	16	0.40	1	Pyridine *N*-oxide	DCM	34
**5**	16	0.40	1	Pyridine *N*-oxide	DCM	28
**10**	16	0.40	1	Pyridine *N*-oxide	DCM	19
1	**1**	0.40	1	Pyridine *N*-oxide	DCM	22
1	**2**	0.40	1	Pyridine *N*-oxide	DCM	21
1	**4**	0.40	1	Pyridine *N*-oxide	DCM	36
1	**6**	0.40	1	Pyridine *N*-oxide	DCM	33
1	6+	**1.6**	1	Pyridine *N*-oxide	DCM	29
1	6+	**0.40**	1	Pyridine *N*-oxide	DCM	37
1	6+	**0.80**	1	Pyridine *N*-oxide	DCM	41
1	6+	**0.53**	1	Pyridine *N*-oxide	DCM	26
1	6+	**0.20**	1	Pyridine *N*-oxide	DCM	30
1	6+	**0.10**	1	Pyridine *N*-oxide	DCM	16
1	6+	**0.80**	**3**	Pyridine *N*-oxide	DCM	40
1	6+	**0.80**	**5**	Pyridine *N*-oxide	DCM	13
1	6+	**0.80**	**7**	Pyridine *N*-oxide	DCM	2
1	6+	0.40	**0.5**	Pyridine *N*-oxide	DCM	19
1	6+	0.40	**1**	Pyridine *N*-oxide	DCM	32
1	6+	0.40	**2**	Pyridine *N*-oxide	DCM	45
1	6+	0.40	**3**	Pyridine *N*-oxide	DCM	58
1	6+	0.40	**4**	Pyridine *N*-oxide	DCM	63
1	6+	0.40	**5**	Pyridine *N*-oxide	DCM	71
1	6+	0.40	**6**	Pyridine *N*-oxide	DCM	70
1	6+	0.40	**7**	Pyridine *N*-oxide	DCM	58
1	6+	0.40	**8**	Pyridine *N*-oxide	DCM	64
1	6+	0.40	**10**	Pyridine *N*-oxide	DCM	59
1	6+	0.40	**5***	Pyridine *N*-oxide	DCM	110
1	6+	0.40	1	**4 phenyl pyridine *N*-oxide**	DCM	35
1	6+	0.40	1	Pyridine *N*-oxide	**$CHCl_3$**	25
1	6+	0.40	1	Pyridine *N*-Oxide	DCM	0*

Table S1. Optimization of trifluoromethylation of **1** *The reaction was performed in the dark, covered by aluminum foil.

Regiochemical Determination of Trifluoromethylation

A series of copolymers and small molecules were synthesized to elucidate the ^{19}F NMR shifts of the trifluoromethyl groups at different locations on the ring, shown below.

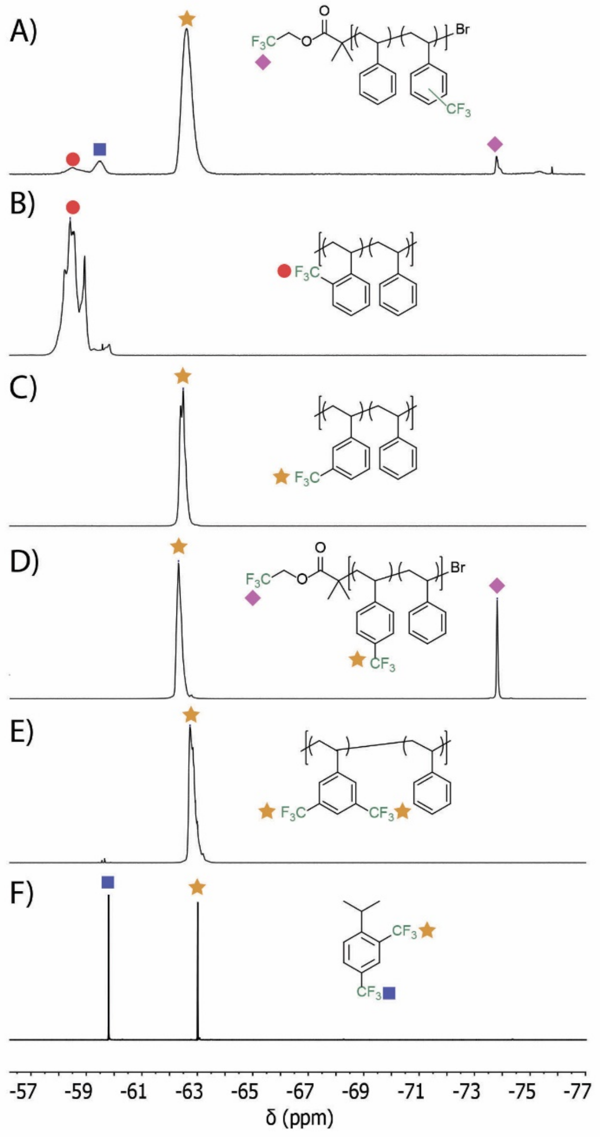

Figure S1. ^{19}FNMR spectra of trifluoromethyl standards, all spectra use fluorobenzene as an internal standard at -113.15 ppm. (A) trifluoromethylated **1**, this copolymer contains different regioisomers of trifluoromethyl styrene resulting in multiple ^{19}F NMR peaks. The peak at -74 ppm corresponds to the polymer chain end and is used as an internal standard. (B) poly(styrene-*co*-2-trifluoromethylstyrene), the peak at -58 ppm corresponds to *ortho*-site functionalization. (C) poly(styrene-*co*-3-trifluoromethylstyrene), the peak at -63 ppm corresponds to *meta*-site functionalization. (D) poly(styrene-*co*-4-trifluoromethylstyrene), the peak at -63 ppm corresponds to *para*-site functionalization. The peak at -74 ppm is the polymer chain end. (E) poly(styrene-*co*-3,5-bis(trifluoromethyl)styrene), the peak at -63 ppm corresponds to di-functionalized styrene at the 3,5-positions. (F) 2'4,'-bis(trifluoromethyl)cumene, this small molecule serves as model for 2,4-bis(trifluoromethyl)styrene repeat units. The peak at -63 ppm corresponds to the *para*-site on this di-functionalized, the peak at -60 ppm corresponds to the *ortho*-site.

Increasing Reagent Equivalents (X)

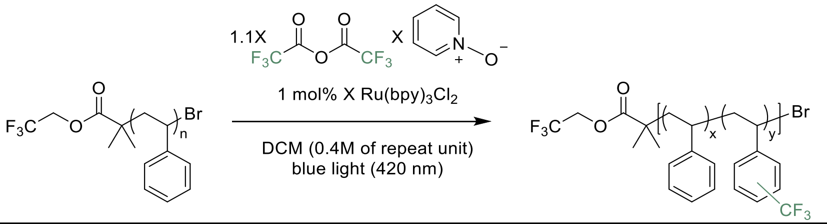

X	Average CF_3 per 100 repeat units	Standard deviation ǂ	Percent *ortho* site functionalization*	Percent *para/meta* site functionalization*	Percent benzylic site functionalization*
0.5	19	1.5	8.3%	88%	3.7%
1	32	2.4	9.3%	88%	2.5%
2	45	4.2	10%	87%	2.3%
3	58	3.4	13%	85%	2.2%
4	63	3.9	14%	84%	2.9%
5	71	8.3	15%	84%	1.1%

Table S2. Reagent equivalent effect on mol% functionalization ǂ Each experiment was run in triplicate, resulting in a standard deviation. *These values are a percentage of the functionalized repeat units, not of all repeat units.

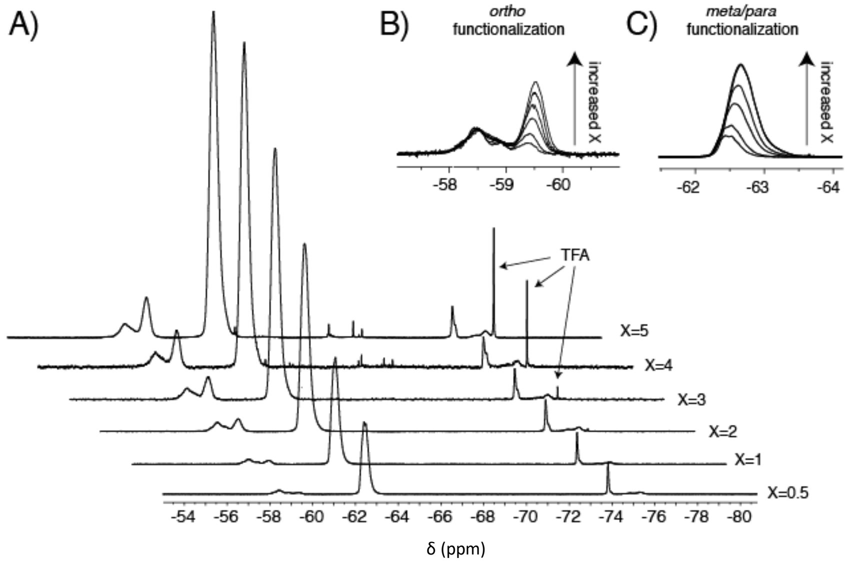

Figure S2. ^{19}F NMR of trifluoromethylated **1** with varying equivalents (X) of reagents. (A) Overlay of ^{19}F NMR spectra of trifluoromethylated **1** with increasing mol% functionalization. TFA is trifluoroacetic acid impurity. (B) Inset of ^{19}F NMR representing *ortho*-trifluoromethylation, the spectra are normalized to the peak at -58.5 ppm to demonstrate the relative increase of the peak at -59.5 as mol% functionalization increases. We hypothesize that the peak at -59.5 ppm represents repeat units with two trifluoromethyl group at the 2,4 positions and increases relative to the monofunctionalized *ortho*-trifluoromethylated repeat units as overall functionalization increases. (C) Inset of ^{19}F NMR representing *para/meta* trifluoromethylation, the spectra are normalized to the peak at -73.8 (the chain end) to demonstrate the increase of *para/meta* trifluoromethylation as higher equivalents of reagents are added.

Gordon-Taylor Theory

The glass transition temperature of the various regioisomers of poly(trifluoromethyl styrene) have been reported in literature and are listed below.[1] Additionally, the T_g values for the trifluoromethylated random copolymers synthesized in this work are also listed.

	T_g (°C)
Poly(2-trifluoromethylstyrene)	175
Poly(3-trifluoromethylstyrene)	63
Poly(4-trifluoromethylstyrene)	101
**1**	84
45 mol% trifluoromethylated **1**	75
60 mol% trifluoromethylated **1**	80

Table S3. Glass transition temperature values for known poly(trifluoromethylstyrene) regioisomers.

We hypothesize that the glass transition temperature of the 45 mol% trifluoromethylated **1** decreases due to high amounts of *para-* and *meta-* site functionalization, the least sterically hindered sites. From the literature, *para-* trifluoromethyl groups have little effect on the T_g value of a copolymer while *meta*-functionalization would drastically lower the value, in accordance with Gordon Taylor theory. This is why an initial decrease in T_g is observed. As trifluoromethylation increases, however more *ortho*-functionalization is observed (Fig. S2). Since the homopolymer of poly(*ortho*-trifluoromethyl styrene) has a drastically higher T_g, we speculate that the increase in the T_g seen in trifluoromethylated **1** at higher functionalization (63 mol%) is due to the increased amount of *ortho*-functionalization.

GPC data

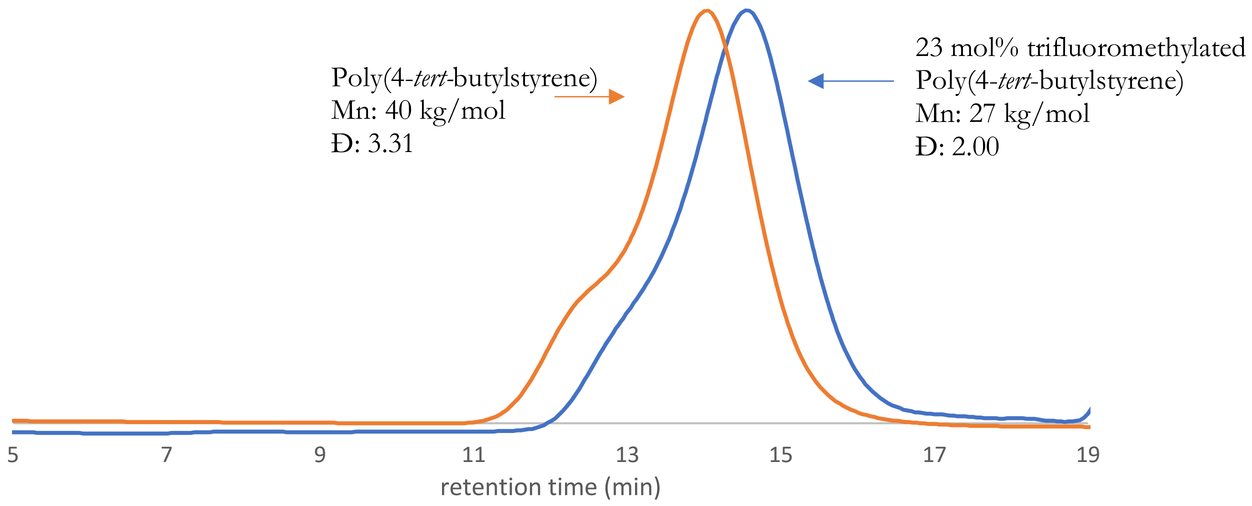

Figure S4. GPC overlay of poly(4-*t*BuStyrene) and functionalized poly(4-*t*BuStyrene). The decrease in M_n is attributed to a decrease in the hydrodynamic radius of the polymer upon trifluoromethylation.

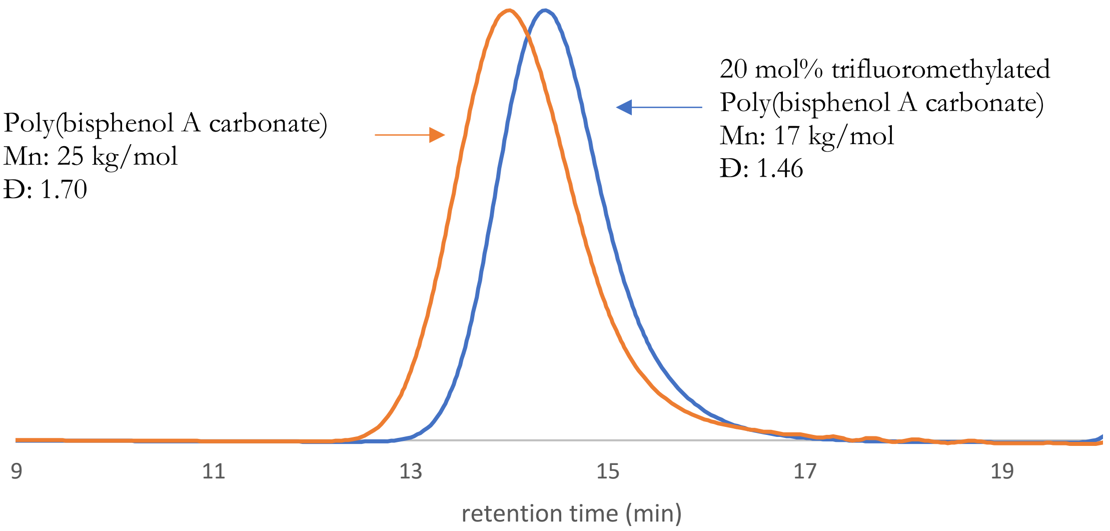

Figure S5. GPC overlay of poly(Bisphenol A carbonate) and functionalized poly(bisphenol A carbonate). The decrease in M_n is attributed to a decrease in the hydrodynamic radius of the polymer upon trifluoromethylation.

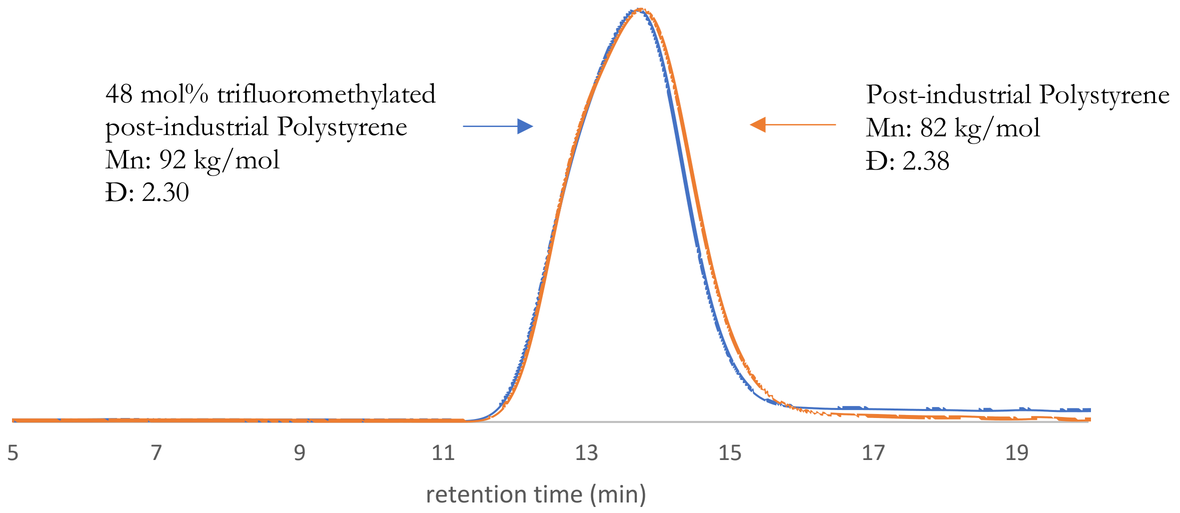

Figure S6. GPC overlay of post-industrial waste polystyrene and functionalized post-industrial waste polystyrene. The increase in M_n is attributed to the addition of mass upon trifluoromethylation.

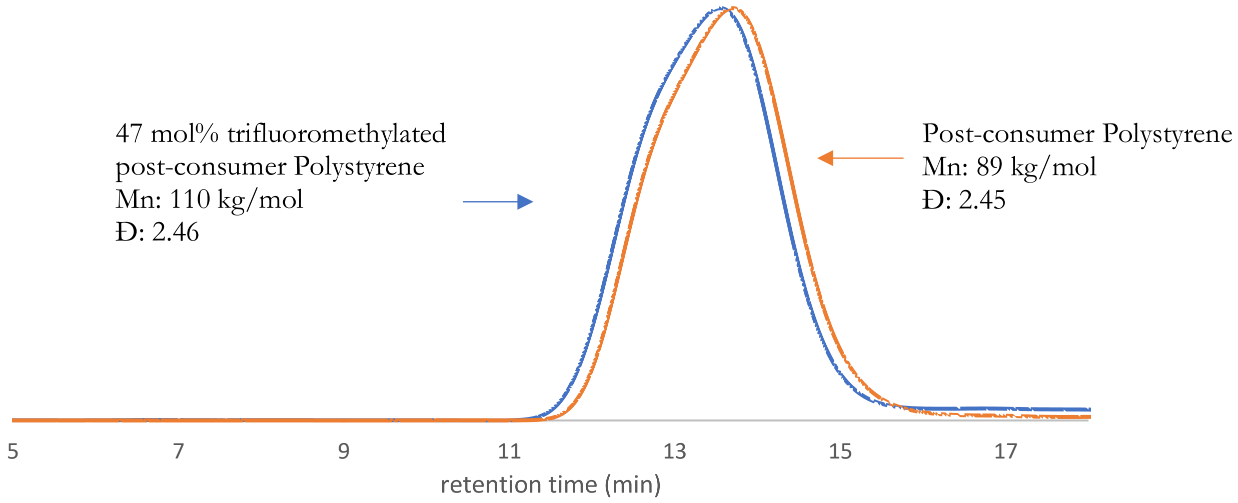

Figure S7. GPC overlay of post-consumer waste polystyrene and functionalized post-consumer waste polystyrene. The increase in M_n is attributed to the addition of mass upon trifluoromethylation.

GPC Traces of Graft Copolymers from Bromodifluoromethylated Polystyrene

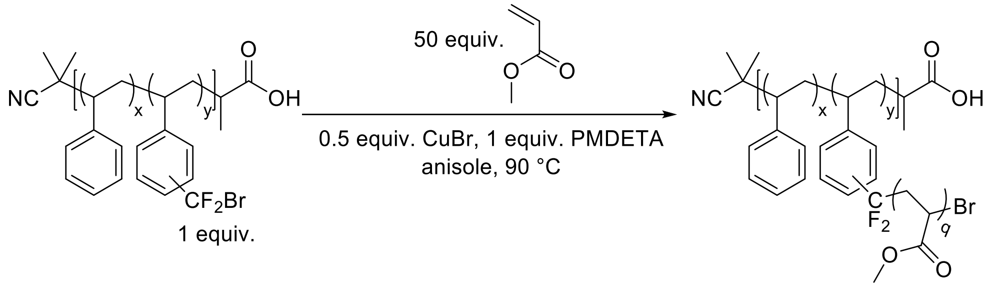

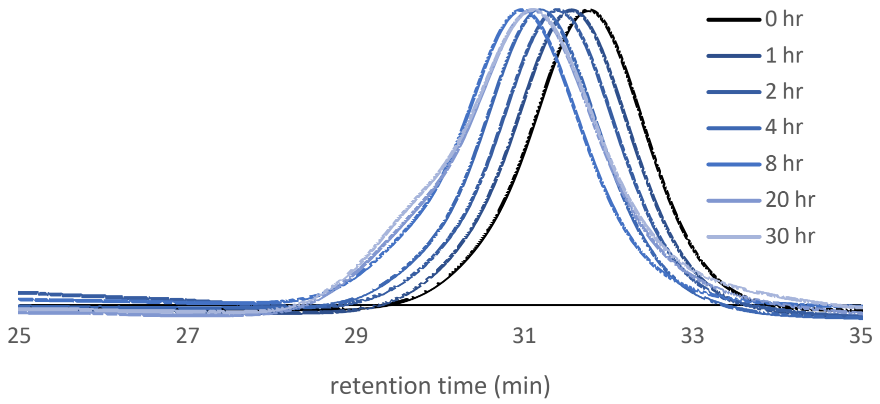

Figure S8. GPC traces over time of the formation of poly(styrene-*graft*-methyl acrylate)

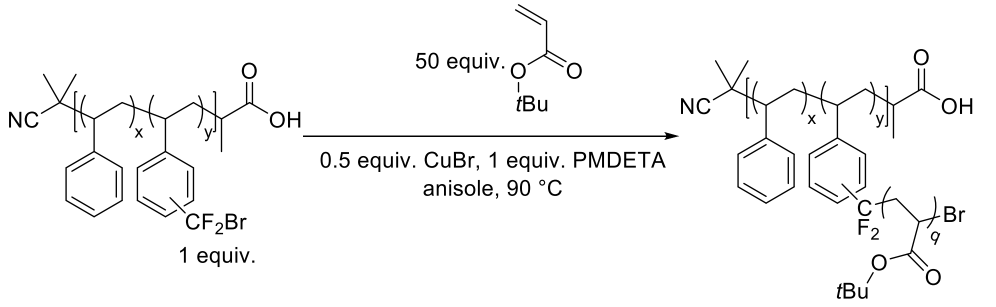

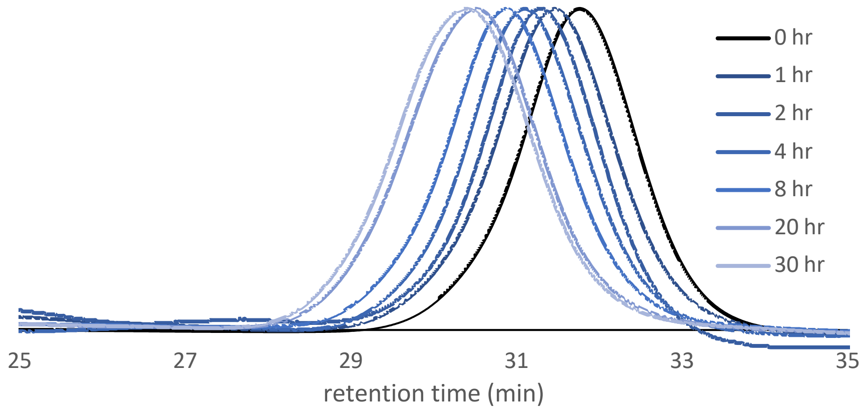

Figure S9. GPC traces over time of the formation of poly(styrene-*graft*-*tert*-butyl acrylate)

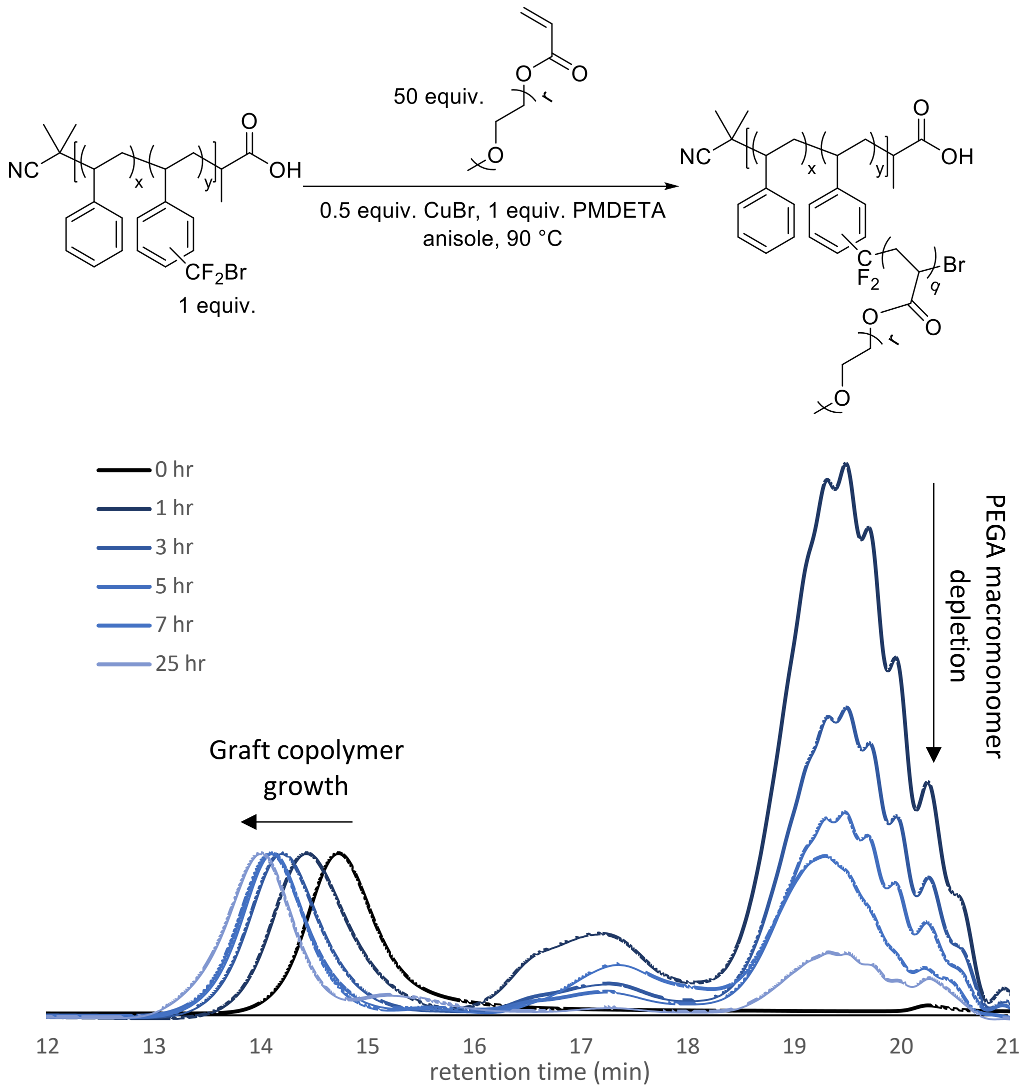

Figure S10. GPC traces over time of the formation of poly(styrene-*graft*-poly(ethylene glycol) acrylate)

Thermal Characterization of Poly(styrene-*graft*-*t*BuA)

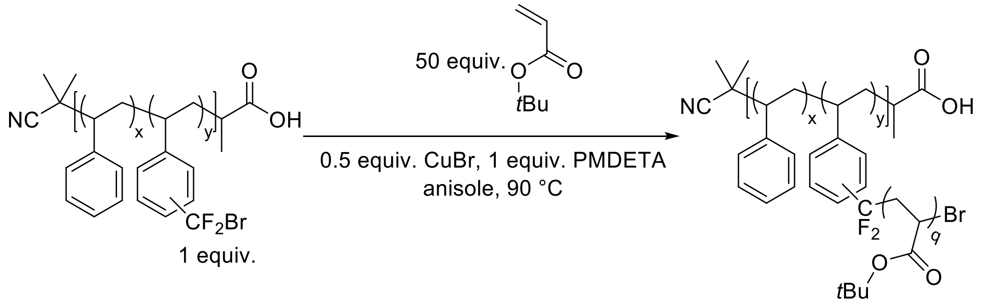

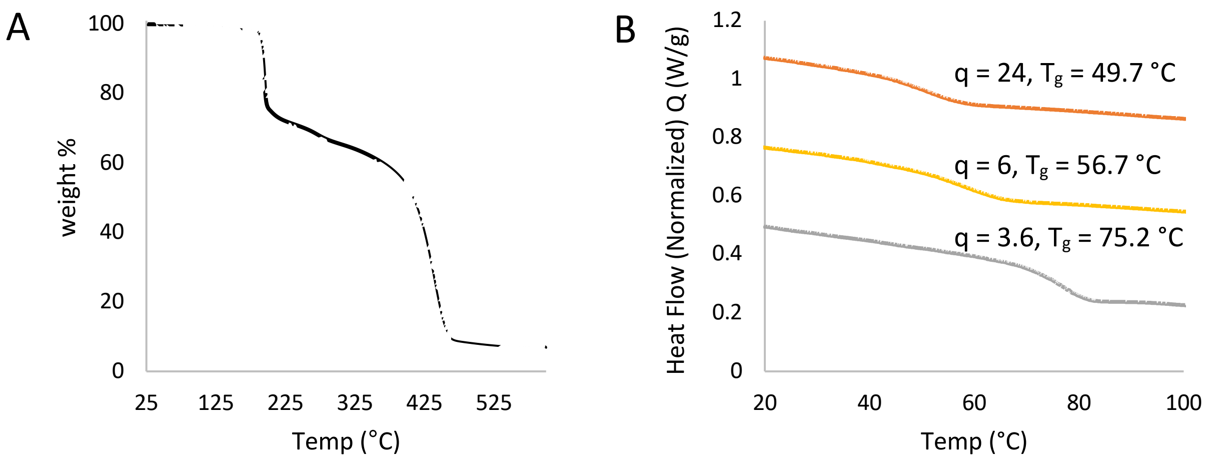

Figure S11. (A) Representative TGA of poly(styrene-*graft*-*tert*-butyl acrylate), with a degree of polymerization (q) of 6 for *tert*-butyl. Decrease in mass at 200 °C is from thermal expulsion of isobutylene. (B) DSC of poly(styrene-*graft*-*tert*-butyl acrylate) with different degrees of polymerization of *tert*-butyl acrylate. These T_gs are between that of polystyrene and poly(*tert*-butyl acrylate) and further confirm the covalent connection between the two otherwise immiscible polymers.

NMR Characterization

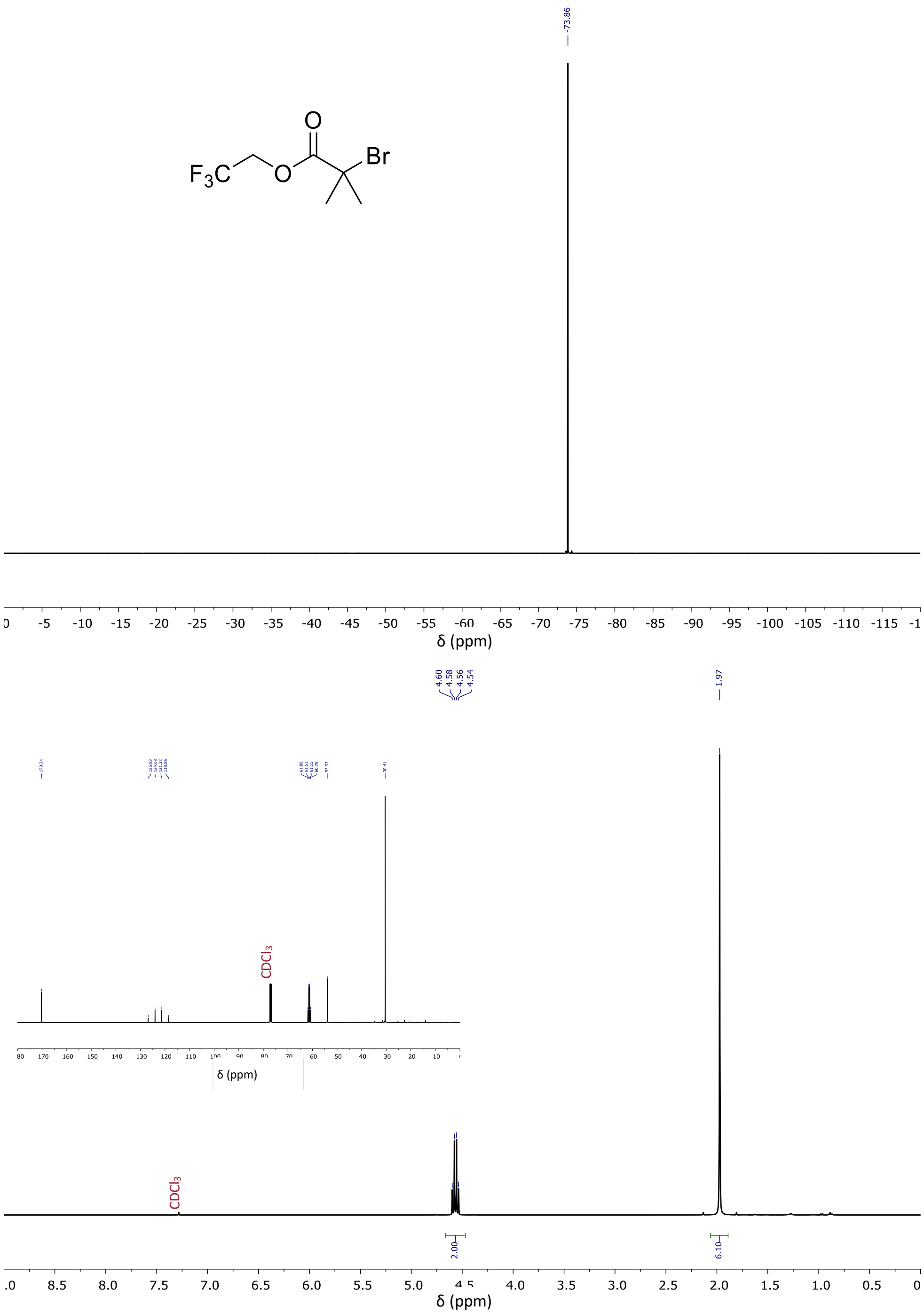

Figure S12: ^{19}F NMR (top), ^{1}H NMR (bottom), and ^{13}C NMR (inset) of 2-trifluoroethyl 2-bromoisobuyrate.

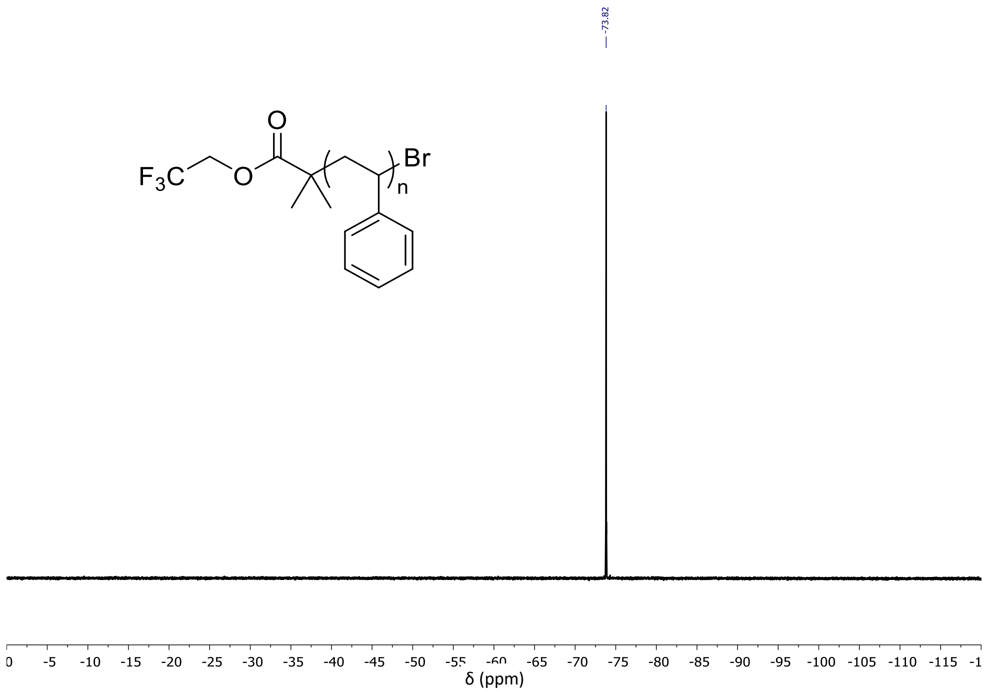

Figure S13: ^{19}F NMR of **1**.

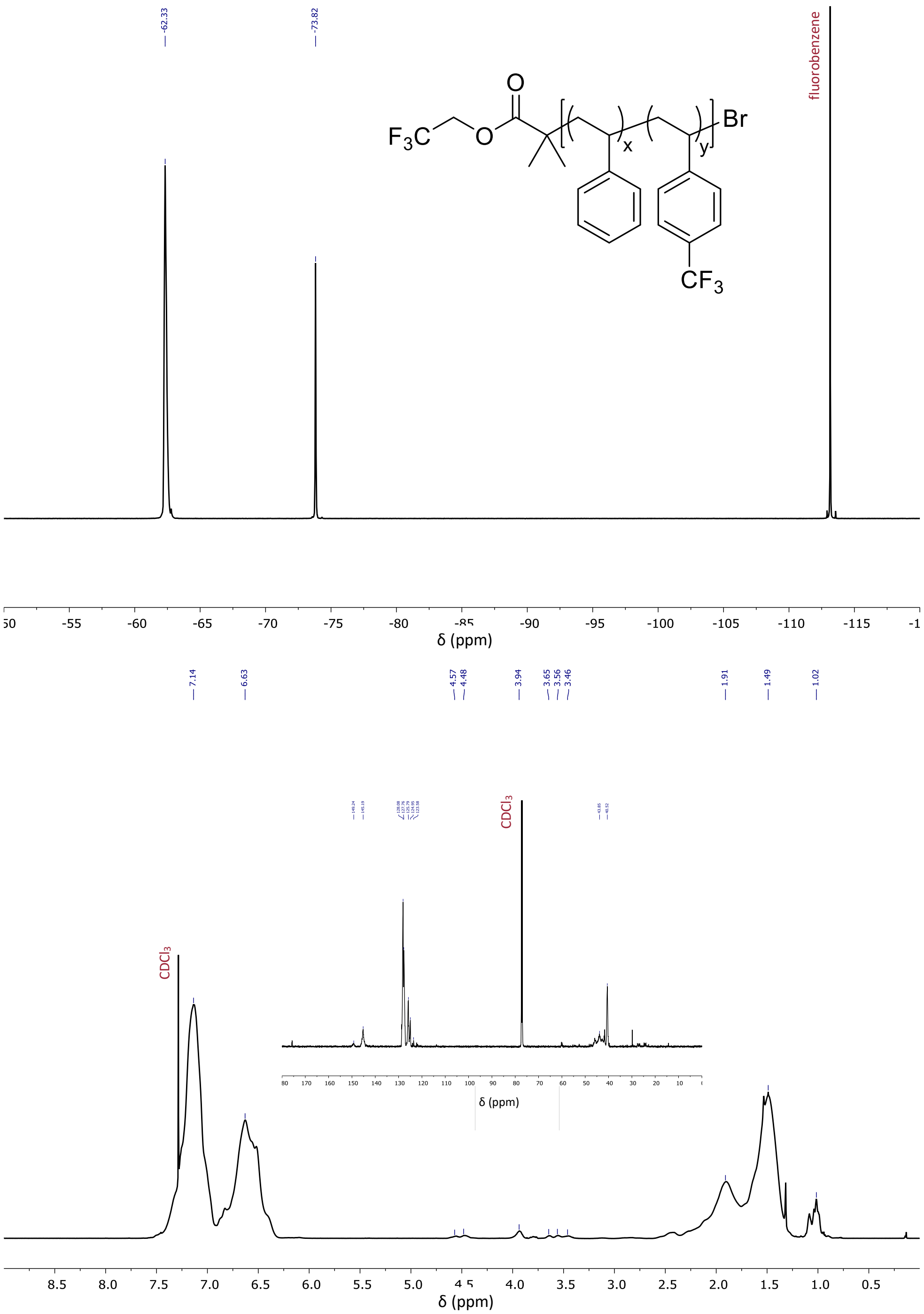

Figure S14: ^{19}F NMR (top), ^{1}H NMR (bottom), and ^{13}C NMR (inset) of poly(styrene-*co-para*-trifluoromethylstyrene). ^{19}F NMR included fluorobenzene as an internal standard (-113.15 ppm).

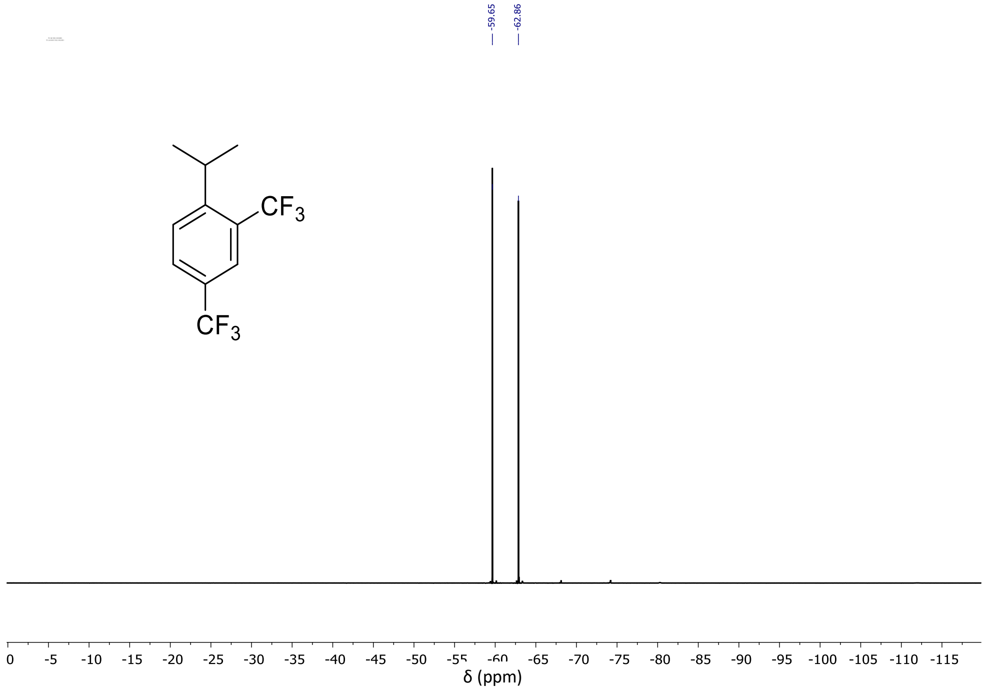

-59.65
-62.86
δ (ppm)

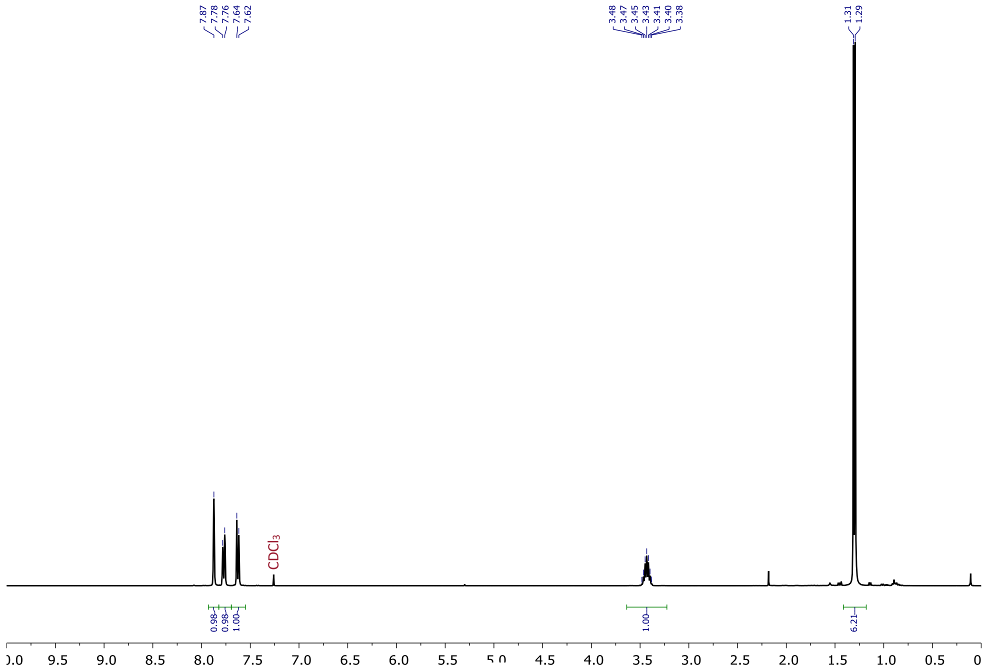

7.87
7.78
7.76
7.64
7.62
3.48
3.47
3.45
3.43
3.41
3.40
3.38
1.31
1.29
CDCl3
0.98
0.98
1.00
1.00
6.21
δ (ppm)

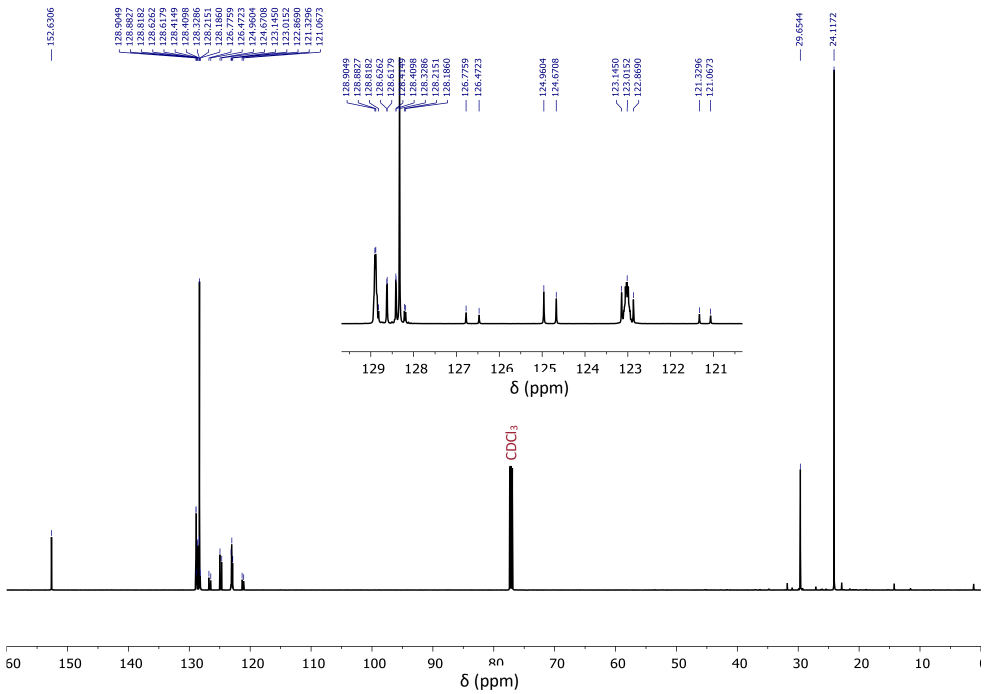

Figure S15: ^{19}F NMR (first), ^{1}H NMR (second), and ^{13}C NMR (third) of 2,4-bis(trifluoromethyl)cumene. ^{13}C NMR inset of the splitting is from ^{19}F nuclei.

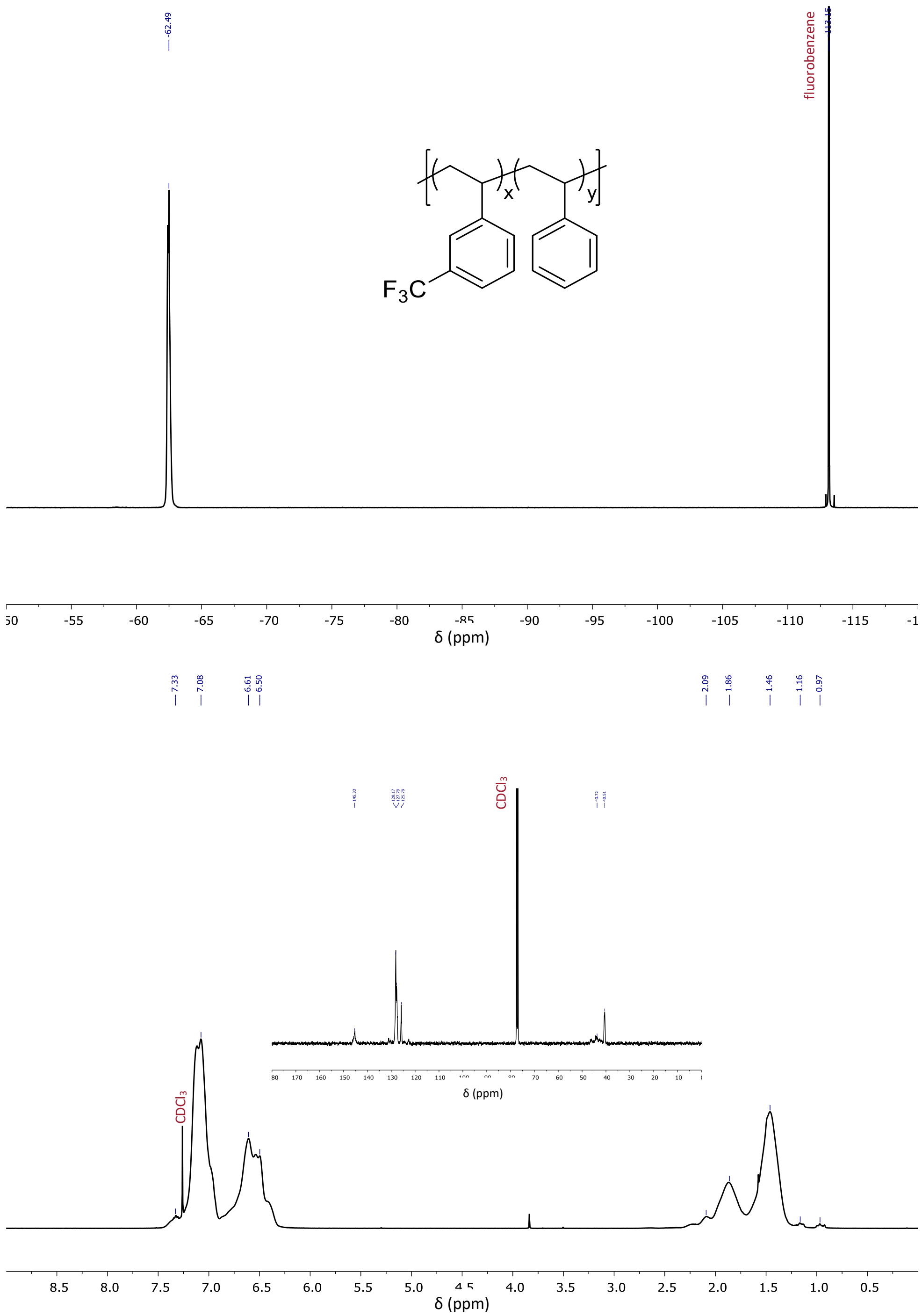

Figure S16: ^{19}F NMR (top), ^{1}H NMR (bottom), and ^{13}C NMR (inset) of the poly(styrene-*co-meta*-trifluoromethylstyrene). ^{19}F NMR included fluorobenzene as an internal standard (-113.15 ppm).

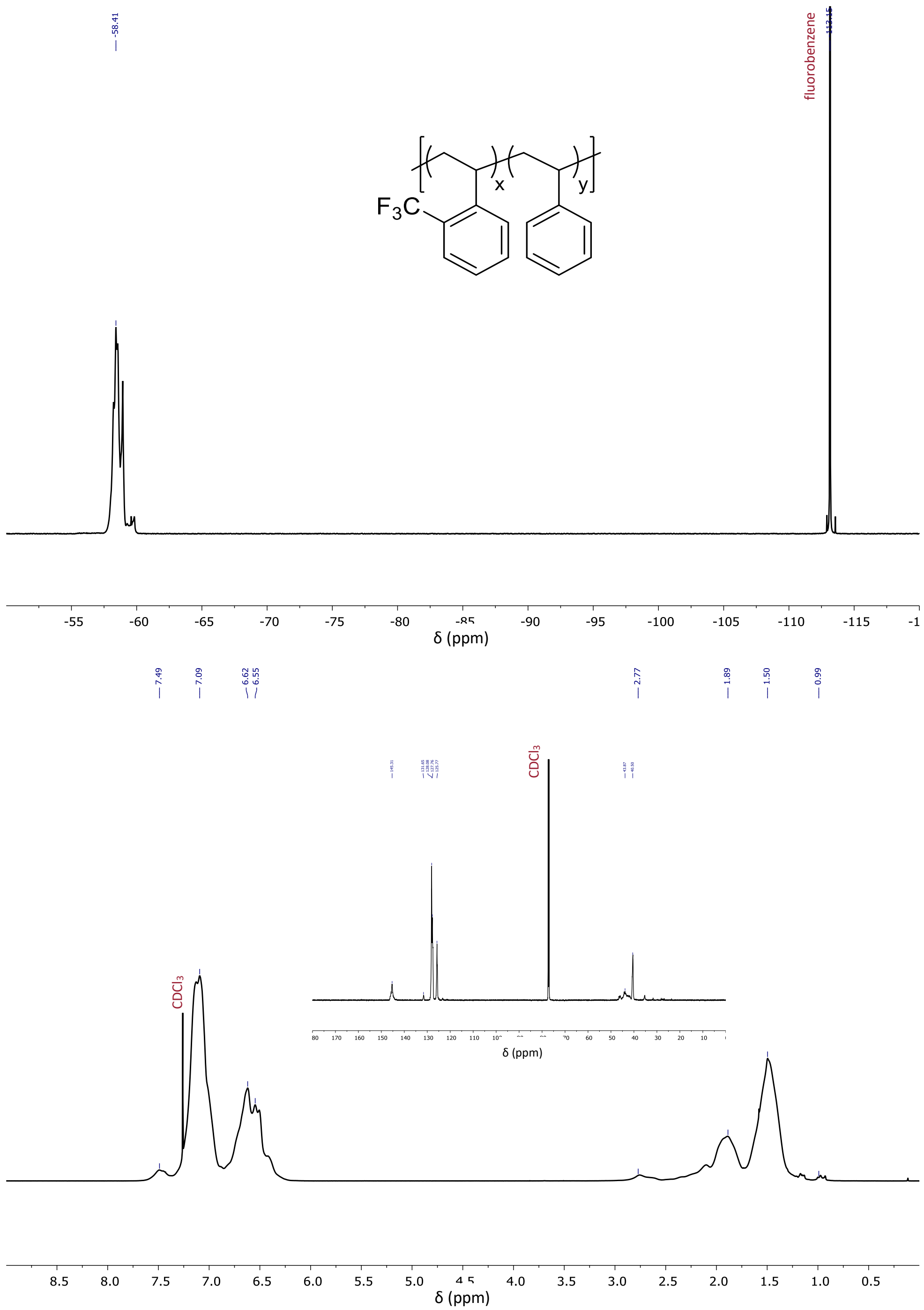

Figure S17: ^{19}F NMR (top), ^{1}H NMR (bottom), and ^{13}C NMR (inset) of poly(styrene-*co*-*ortho*-trifluoromethylstyrene). ^{19}F NMR included fluorobenzene as an internal standard (-113.15 ppm).

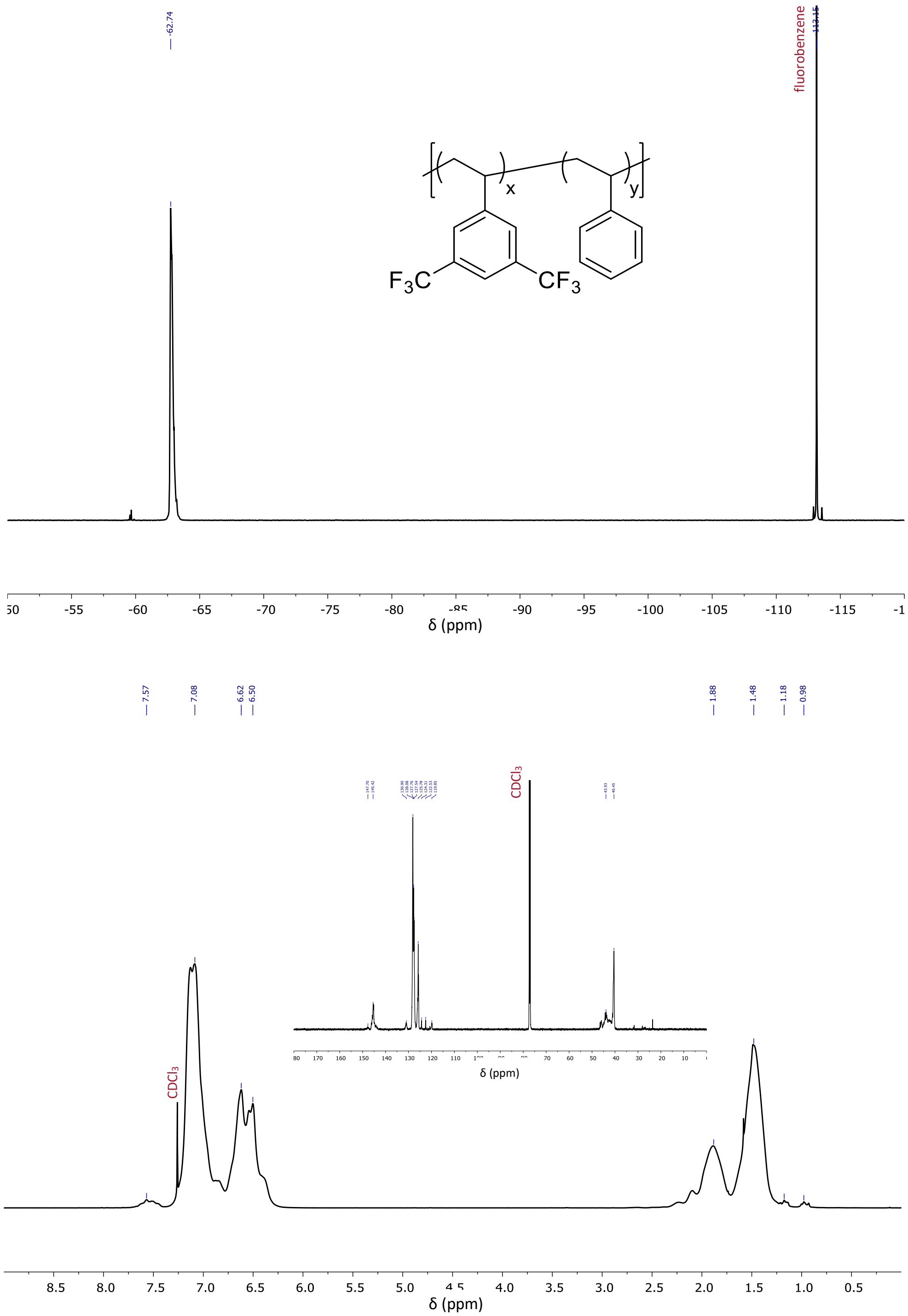

Figure S18: ^{19}F NMR (top), ^{1}H NMR (bottom), and ^{13}C NMR (inset) of poly(styrene-*co*-2,4-bis(trifluoromethyl)styrene). ^{19}F NMR included fluorobenzene as an internal standard (-113.15 ppm).

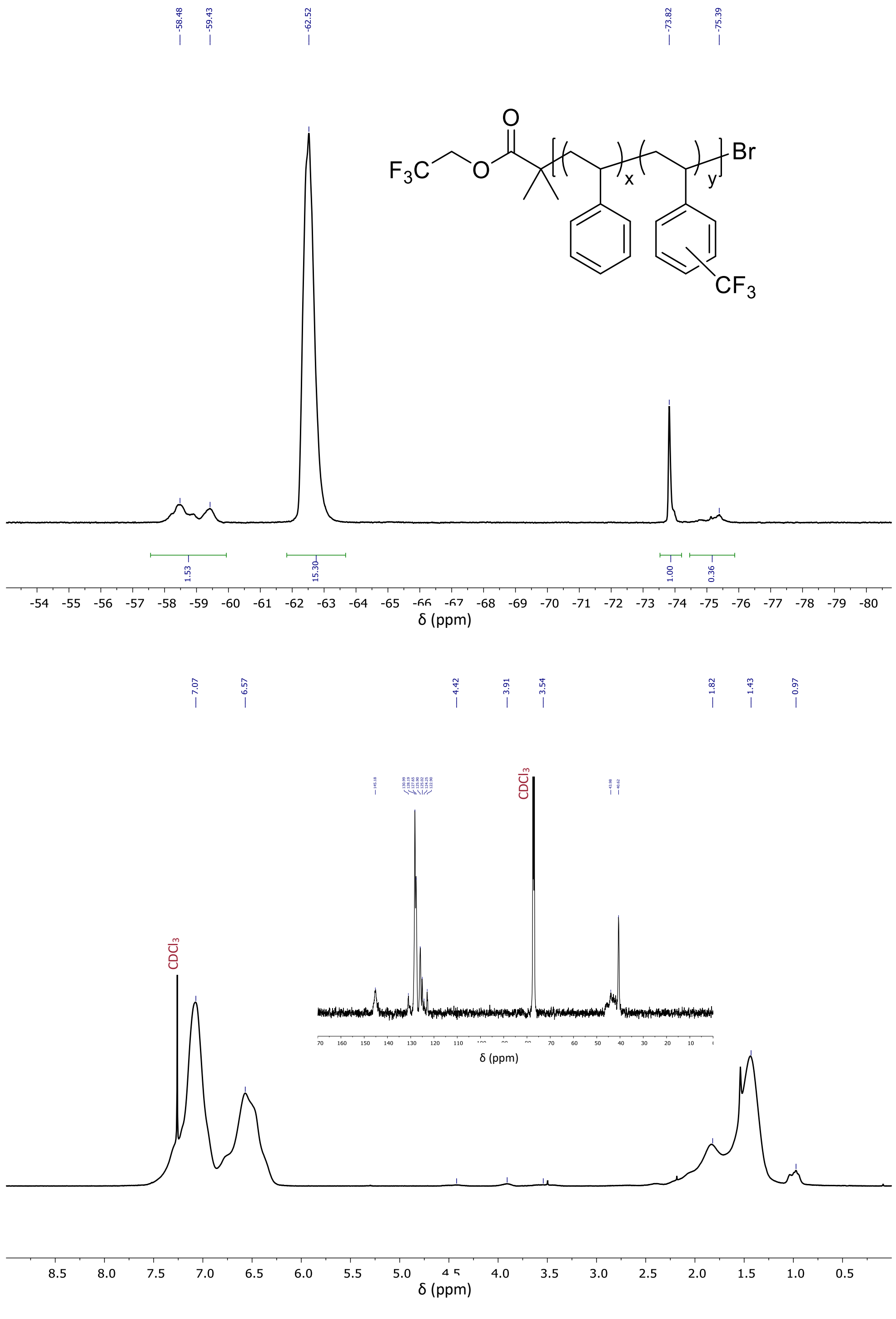

Figure S19: ^{19}F NMR (top), ^{1}H NMR (bottom), and ^{13}C NMR (inset) of trifluoromethylated **1**.

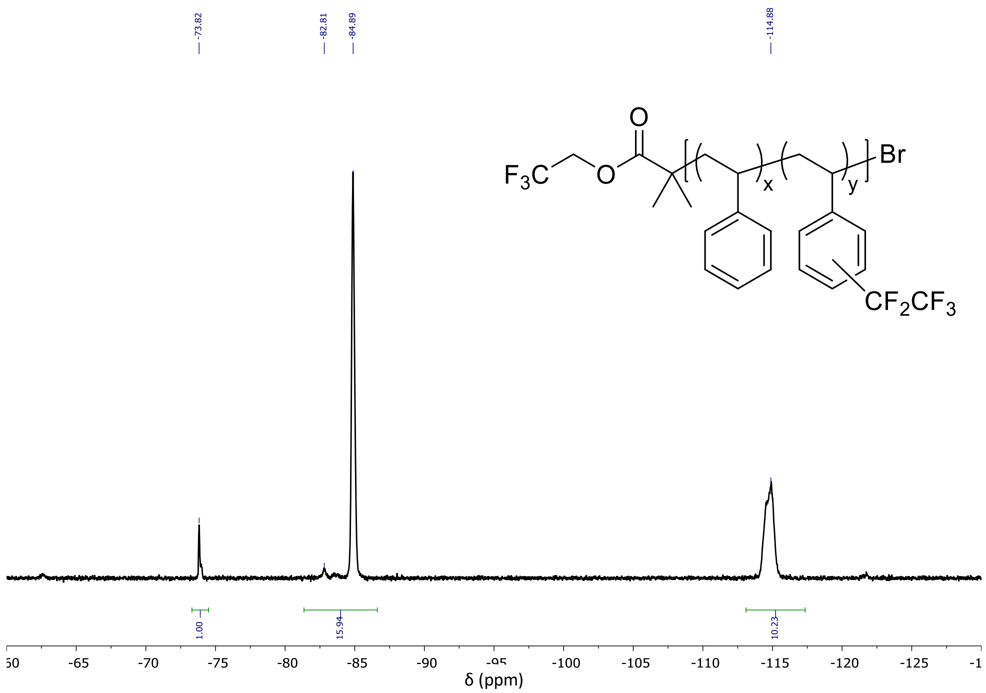
-73.82
-82.81
-84.89
-114.88
F3C
O
O
Br
x
y
CF2CF3
1.00
15.94
10.23
δ (ppm)

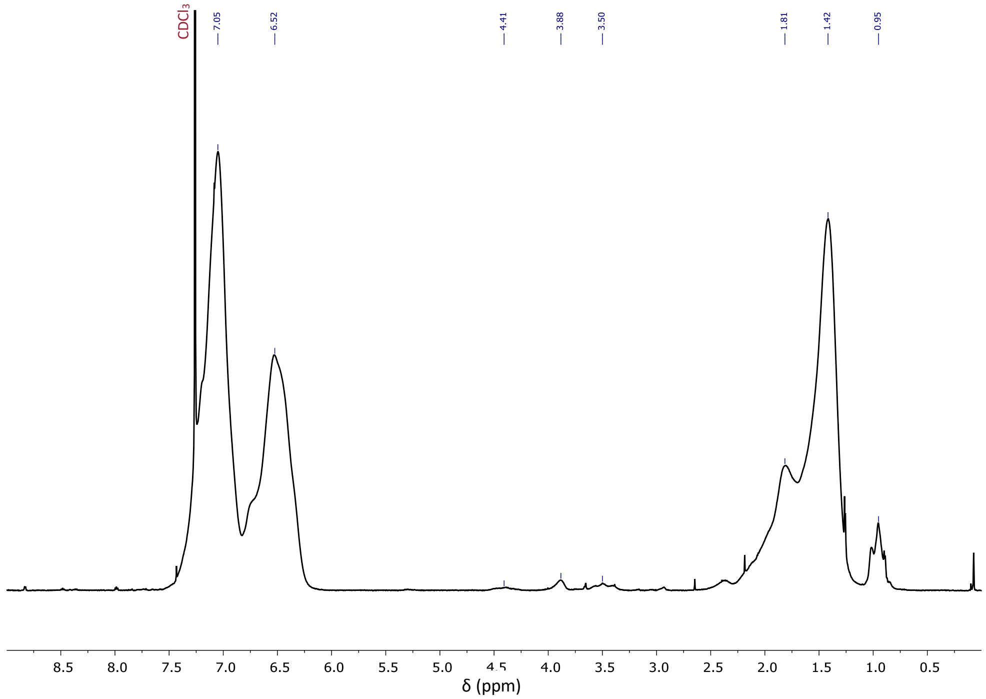
CDCl3
7.05
6.52
4.41
3.88
3.50
1.81
1.42
0.95
δ (ppm)

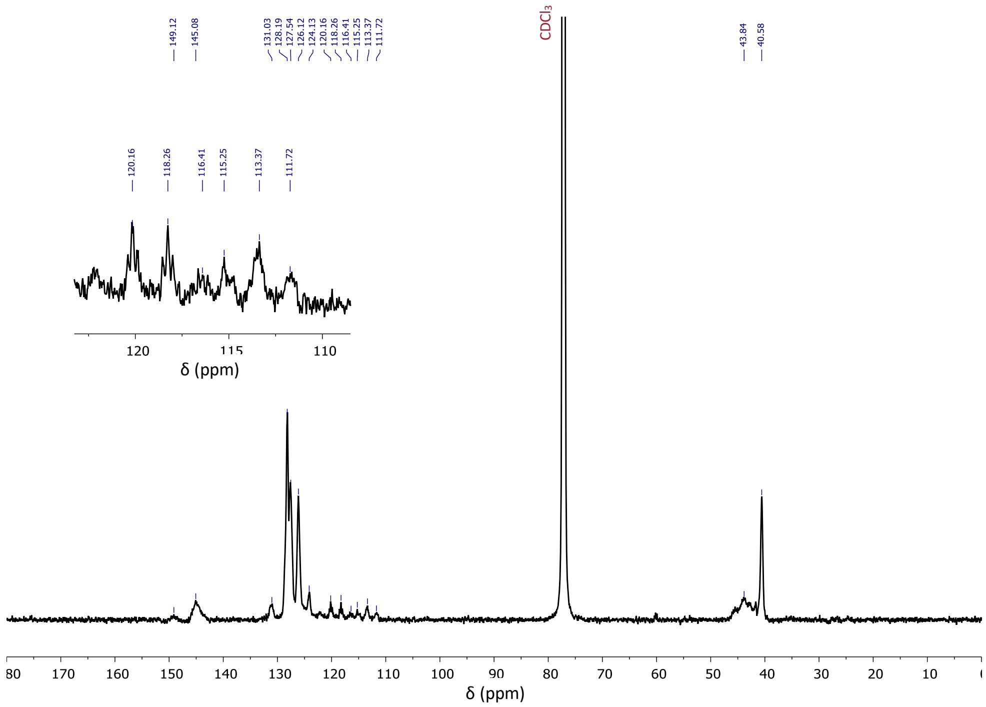

Figure S20: ^{19}F NMR (first), ^{1}H NMR (second), and ^{13}C NMR (third) of perfluoroethylated **1**. The ^{13}C NMR inset shows the complex splitting pattern from ^{19}F nuclei, further proving functionalization of the polymer.

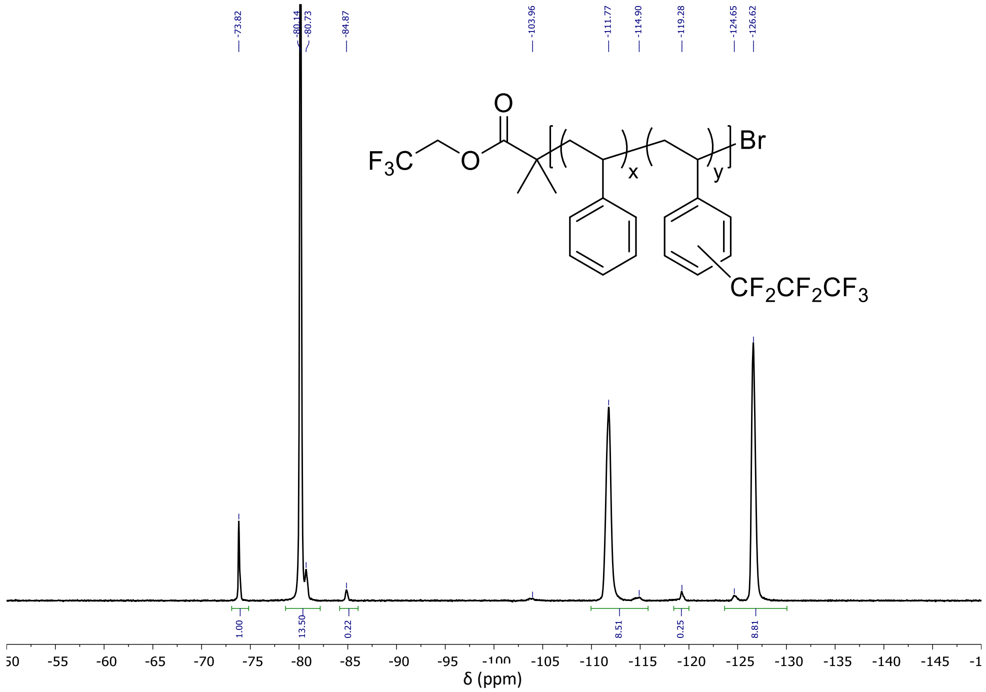
-73.82
-80.14
-80.73
-84.87
-103.96
-111.77
-114.90
-119.28
-124.65
-126.62
F3C
O
O
Br
x
y
CF2CF2CF3
1.00
13.50
0.22
8.51
0.25
8.81
δ (ppm)

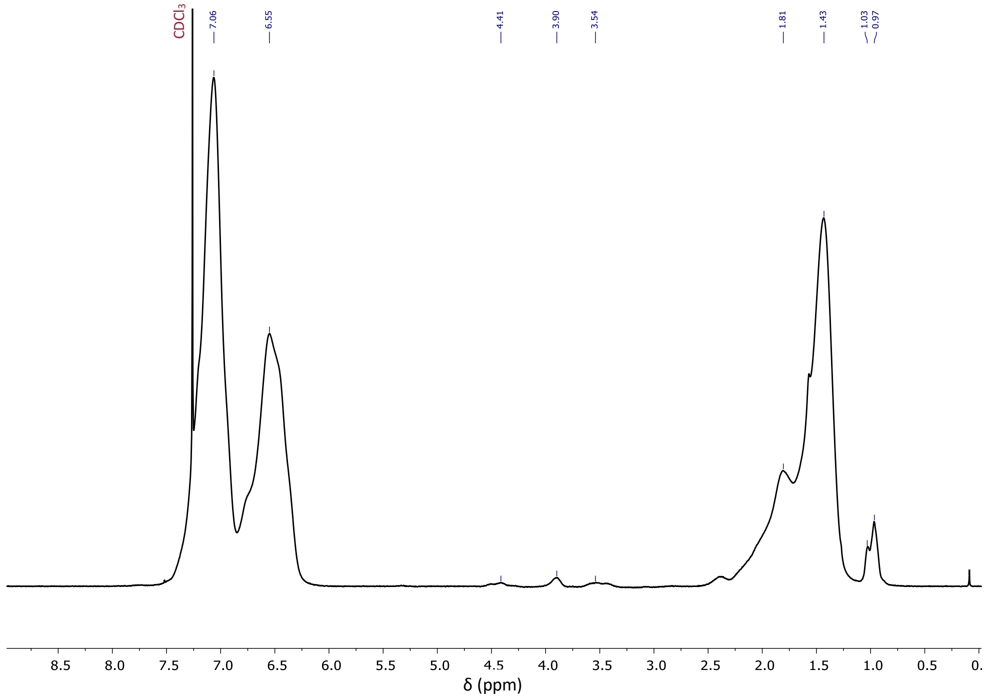
CDCl3
7.06
6.55
4.41
3.90
3.54
1.81
1.43
1.03
0.97
δ (ppm)

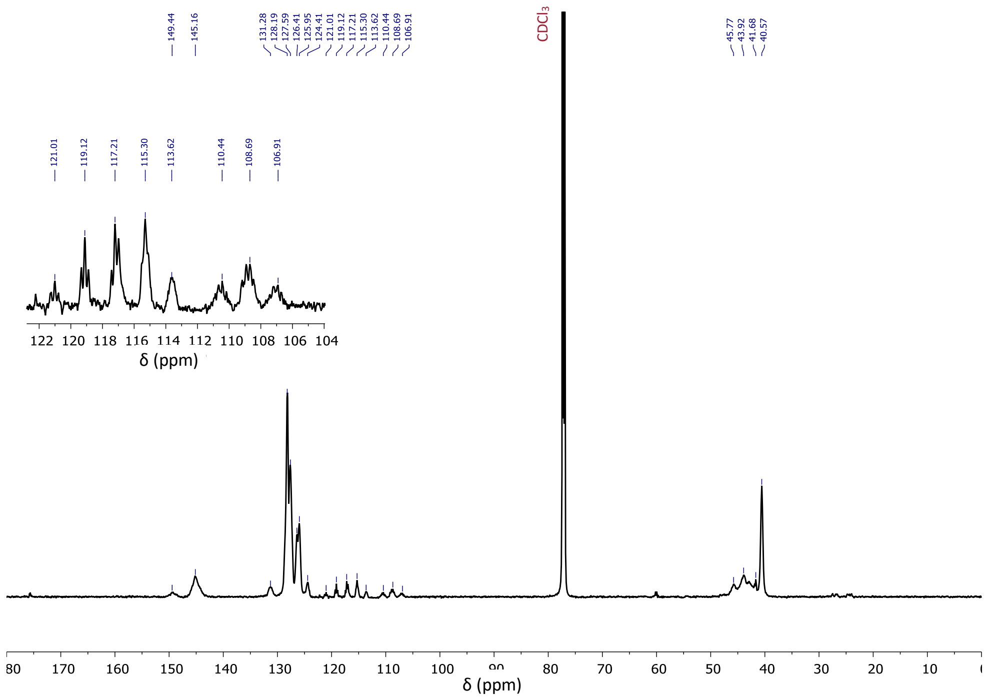

Figure S21: ^{19}F NMR (first), ^{1}H NMR (second), and ^{13}C NMR (third) of perfluoropropylated **1**. The ^{13}C NMR inset shows the complex splitting pattern from ^{19}F nuclei, further proving functionalization of the polymer.

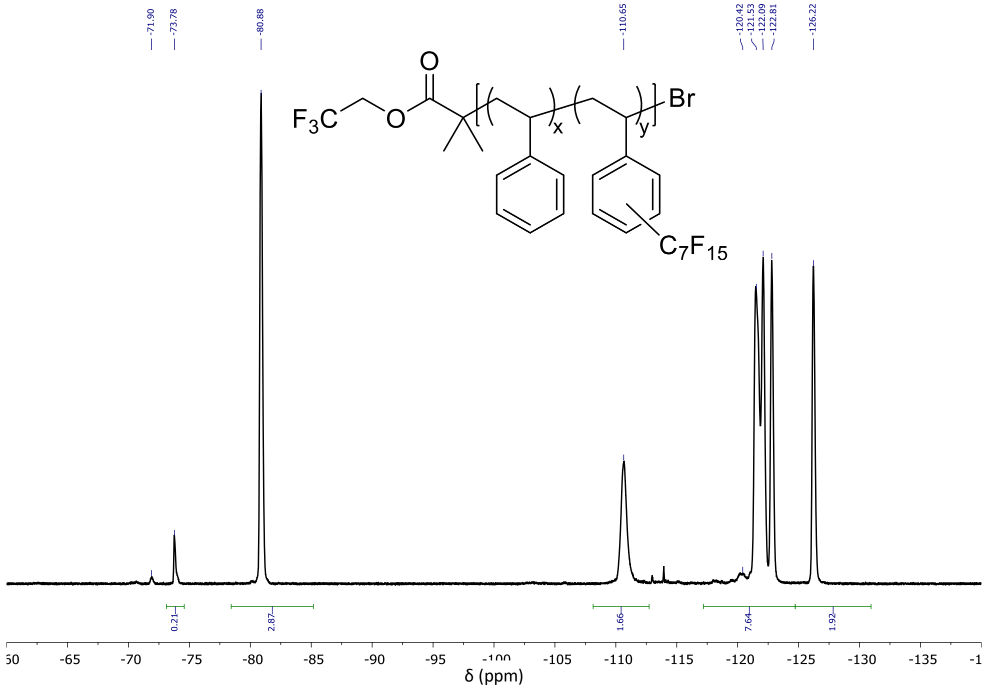
-71.90
-73.78
-80.88
-110.65
-120.42
-121.53
-122.09
-122.81
-126.22
O
F3C
O
x
y
Br
C7F15
0.21
2.87
1.66
7.64
1.92
δ (ppm)

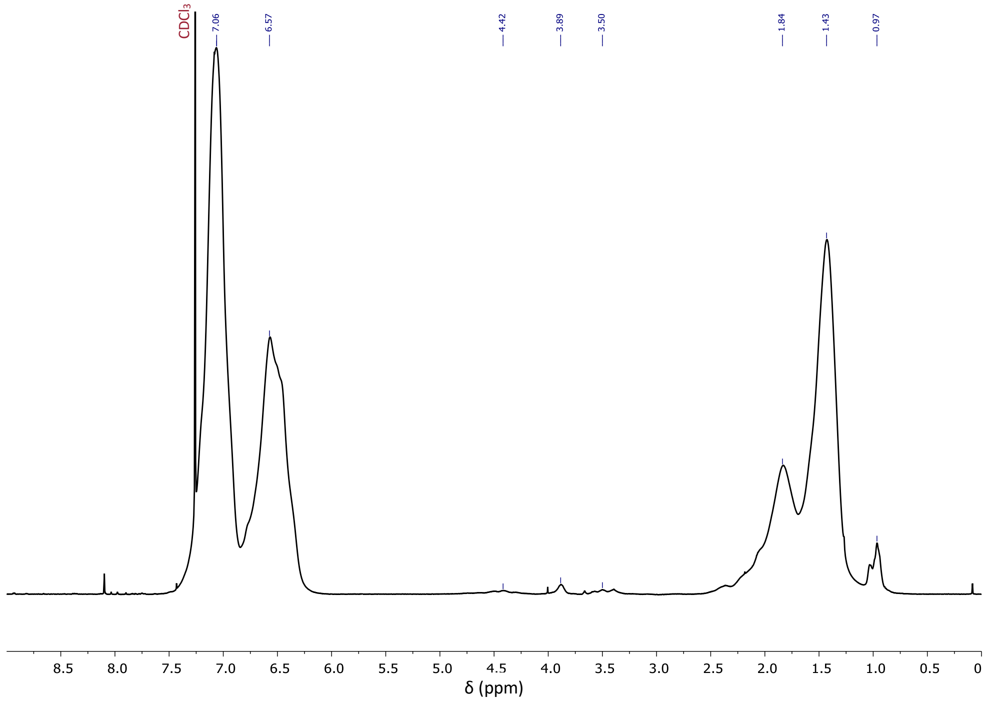
CDCl3
7.06
6.57
4.42
3.89
3.50
1.84
1.43
0.97
δ (ppm)

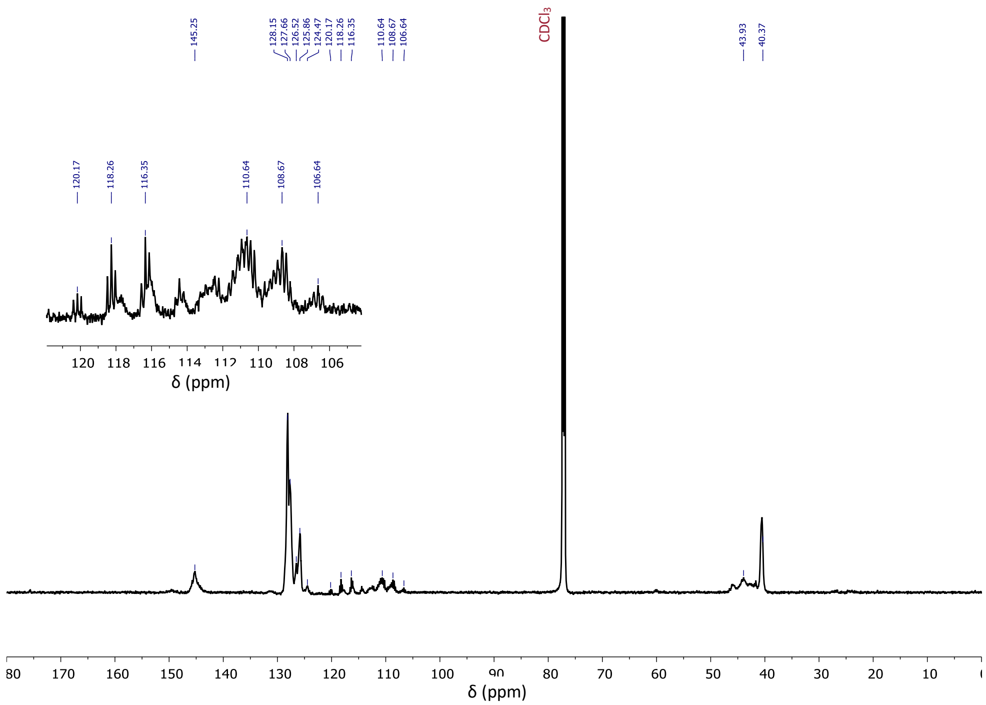

Figure S22: ^{19}F NMR (first), ^{1}H NMR (second), and ^{13}C NMR (third) of perfluoroheptylated **1**. The ^{13}C NMR inset shows the complex splitting pattern from ^{19}F nuclei, further proving functionalization of the polymer.

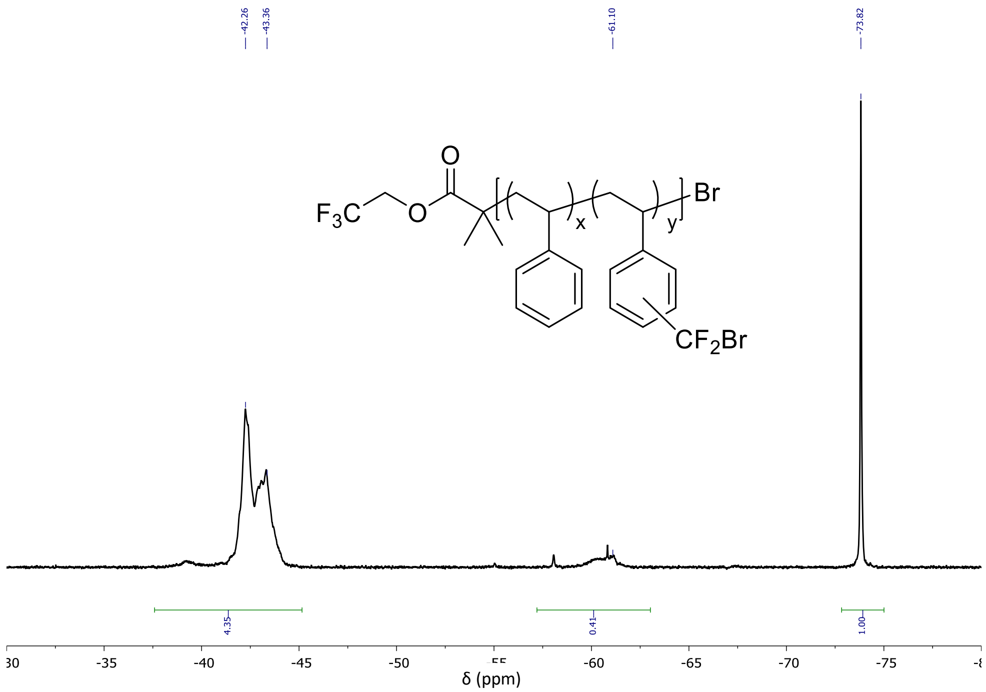
-42.26
-43.36
-61.10
-73.82
O
F3C
O
x
y
Br
CF2Br
4.35
0.41
1.00
-35
-40
-45
-50
-60
-65
-70
-75
δ (ppm)

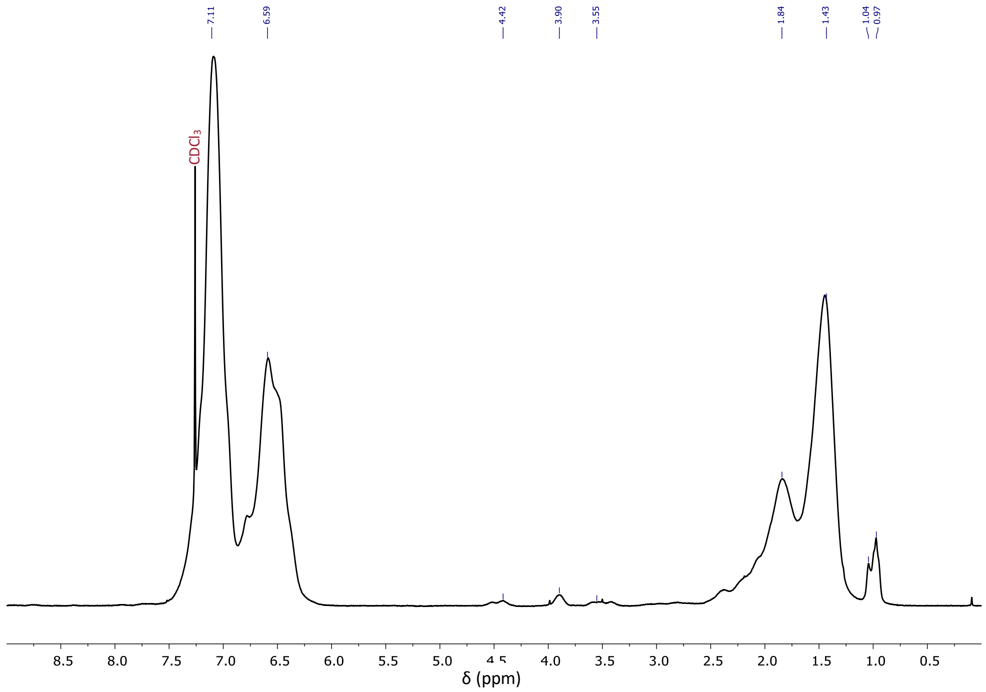
7.11
6.59
4.42
3.90
3.55
1.84
1.43
1.04
0.97
CDCl3
8.5
8.0
7.5
7.0
6.5
6.0
5.5
5.0
4.0
3.5
3.0
2.5
2.0
1.5
1.0
0.5
δ (ppm)

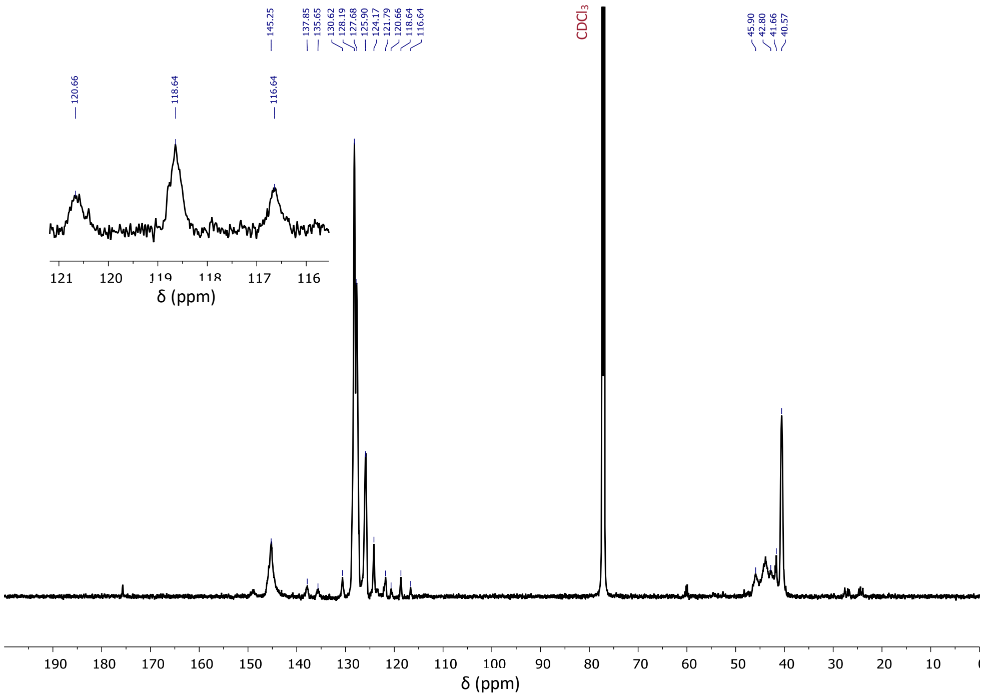

Figure S23: ^{19}F NMR (first), ^{1}H NMR (second), and ^{13}C NMR (third) of bromodifluoroemthylated **1**. The ^{13}C NMR inset shows the triplet pattern from ^{19}F nuclei of the CF_2Br group, further proving functionalization of the polymer.

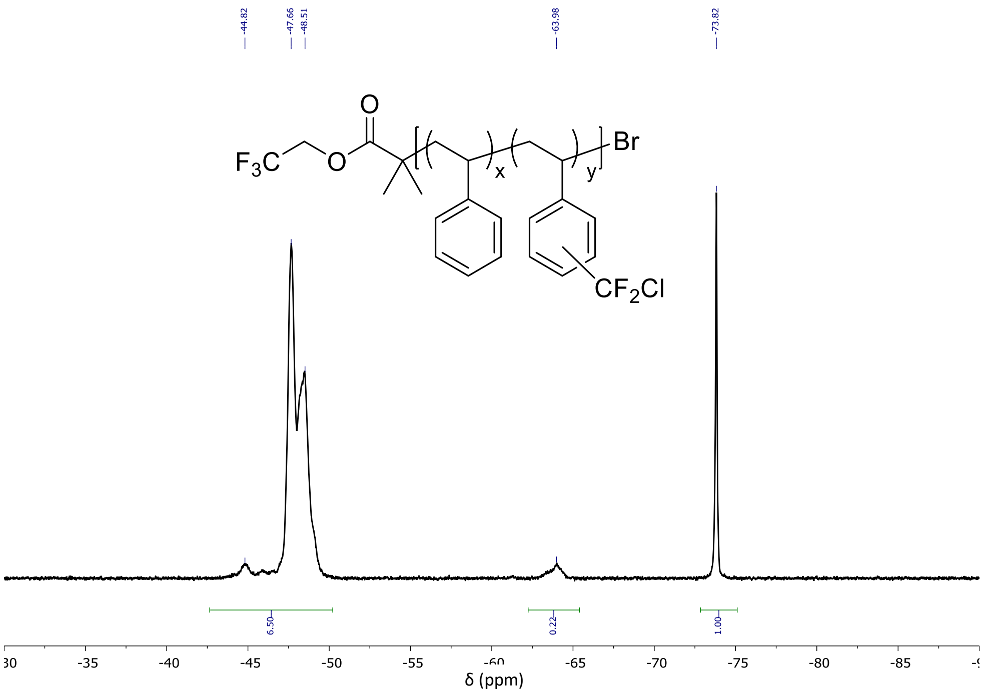

-44.82
-47.66
-48.51
-63.98
-73.82
O
F3C
O
x
y
Br
CF2Cl
6.50
0.22
1.00
30
-35
-40
-45
-50
-55
-65
-70
-75
-80
-85
δ (ppm)

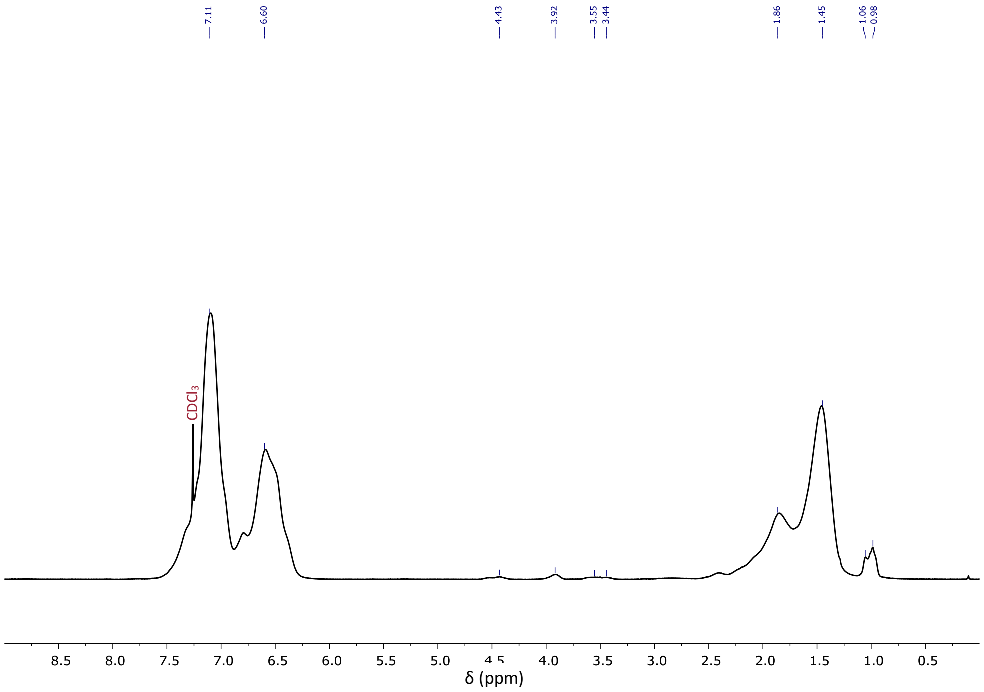

7.11
6.60
4.43
3.92
3.55
3.44
1.86
1.45
1.06
0.98
CDCl3
8.5
8.0
7.5
7.0
6.5
6.0
5.5
5.0
4.0
3.5
3.0
2.5
2.0
1.5
1.0
0.5
δ (ppm)

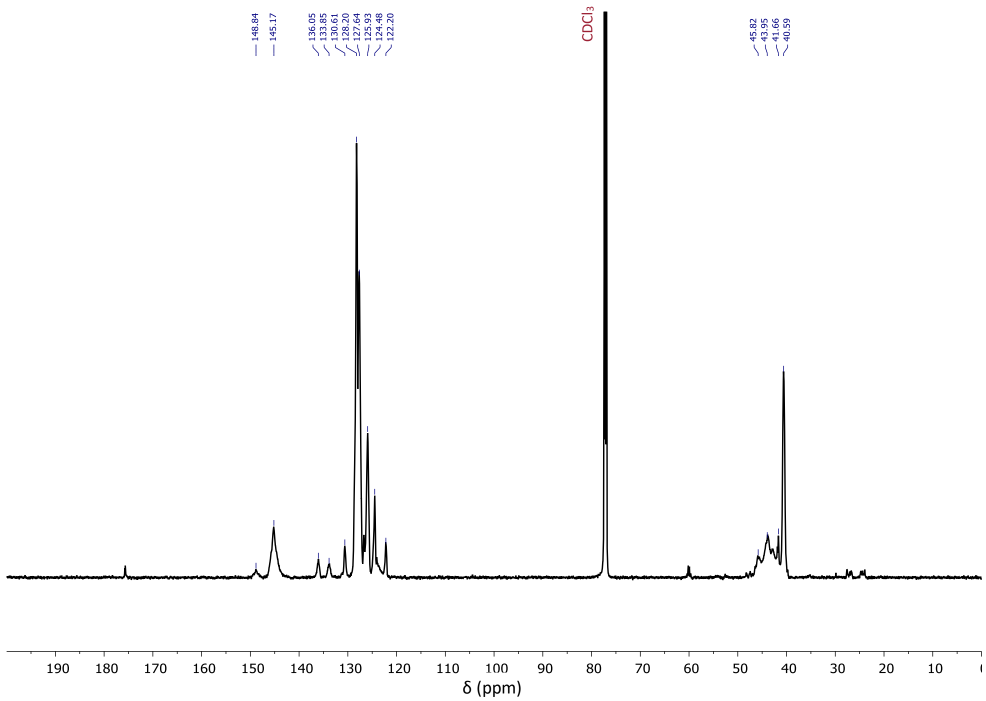

Figure S24: ^{19}F NMR (first), ^{1}H NMR (second), and ^{13}C NMR (third) of chlorodifluoromethylated **1**.

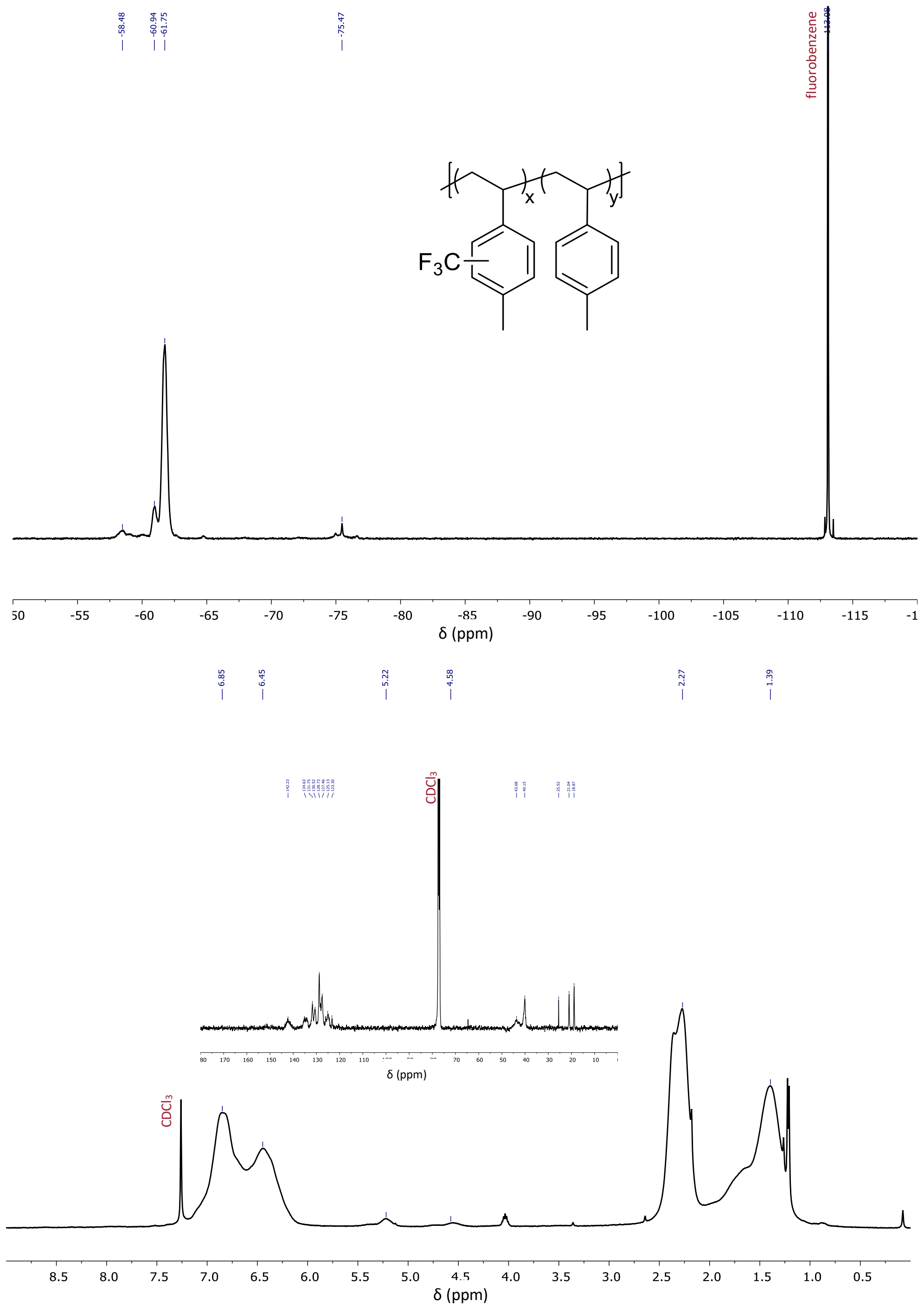

Figure S25: ^{19}F NMR (top), ^{1}H NMR (bottom), and ^{13}C NMR (inset) of trifluoromethylated poly(4-MeStyrene). ^{19}F NMR included fluorobenzene as an internal standard (-113.15 ppm).

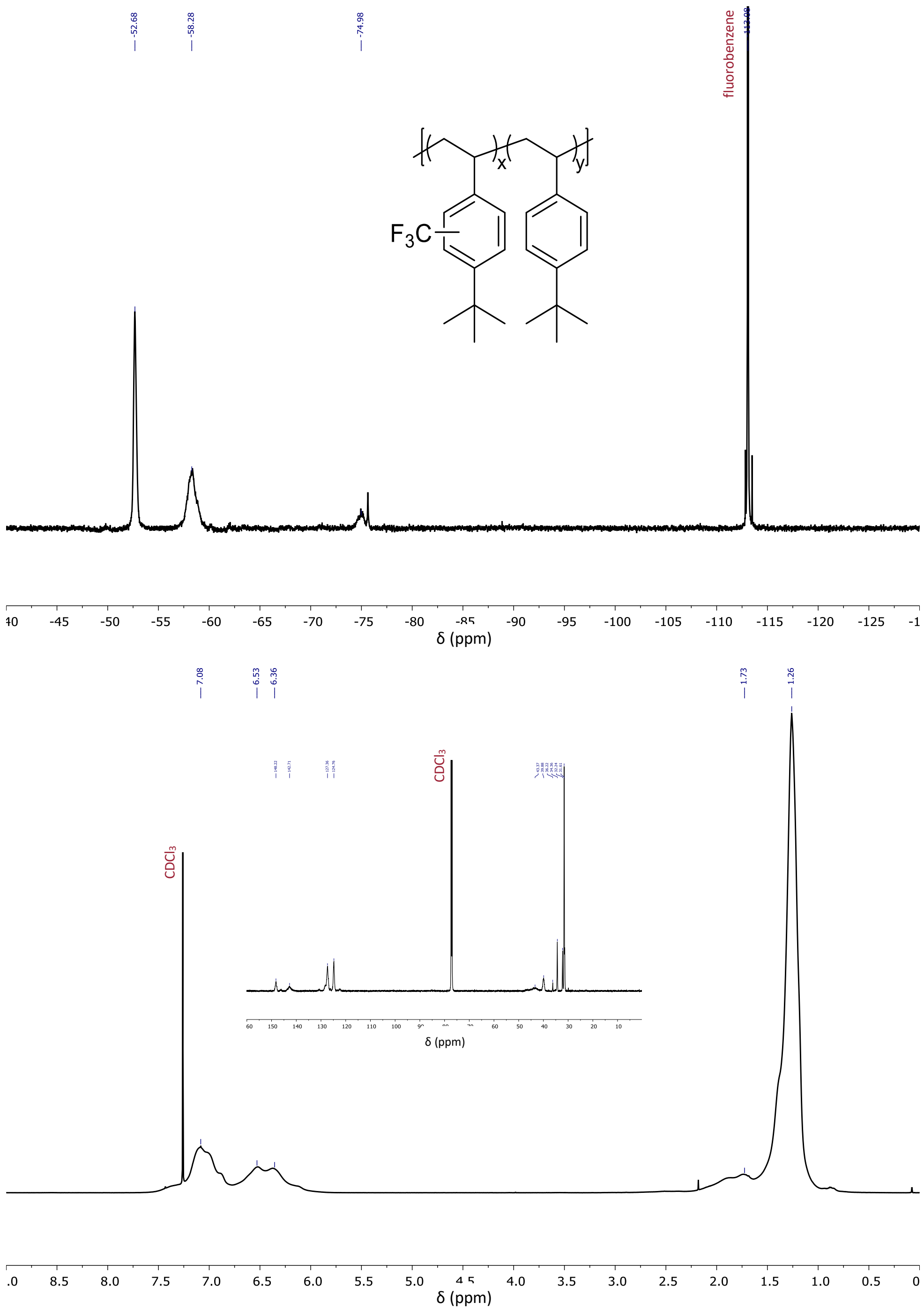

Figure S26: ^{19}F NMR (top), ^{1}H NMR (bottom), and ^{13}C NMR (inset) of trifluoromethylated poly(5-*t*BuStyrene). ^{19}F NMR included fluorobenzene as an internal standard (-113.15 ppm).

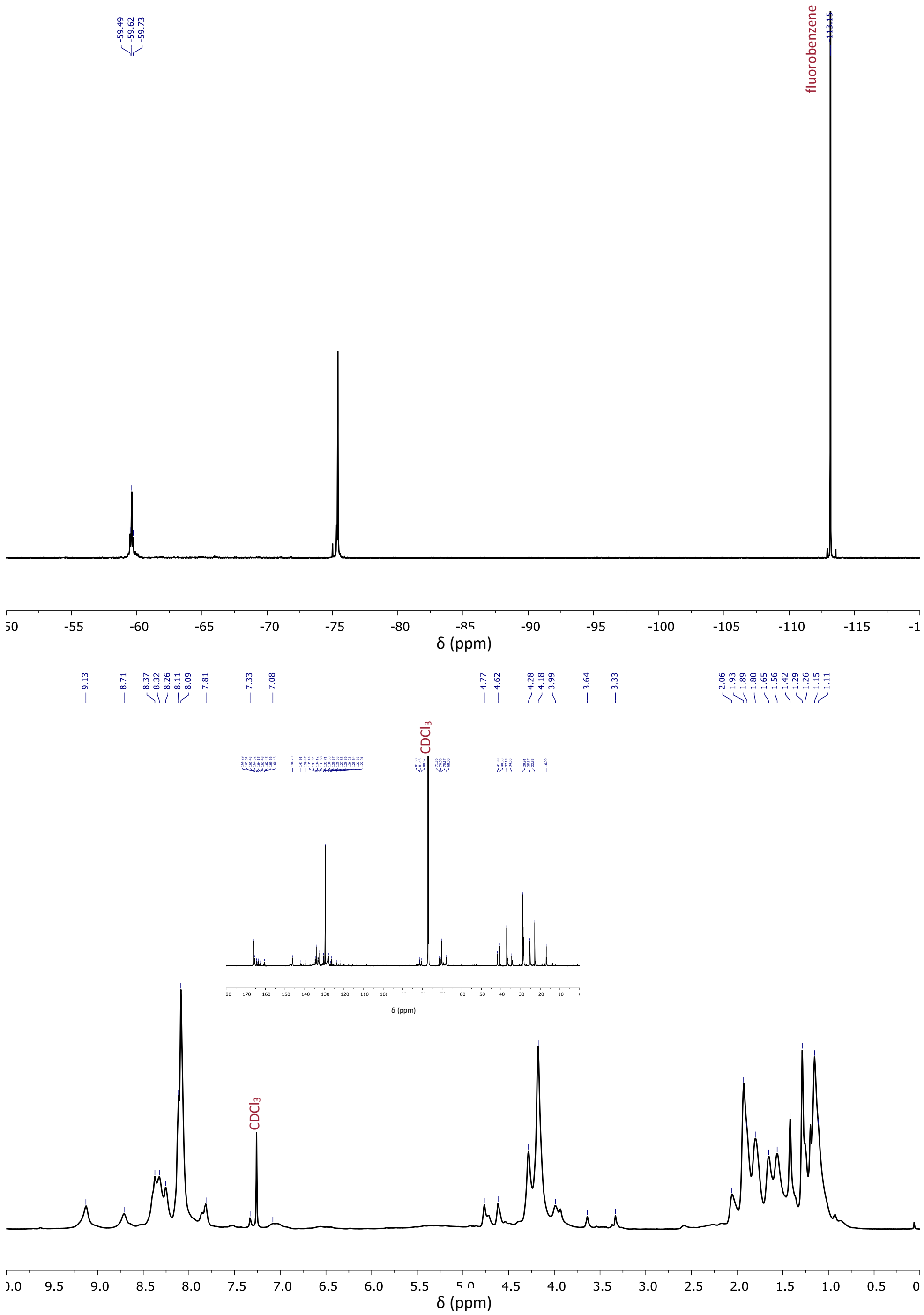

Figure S27: ^{19}F NMR (top), ^{1}H NMR (bottom), and ^{13}C NMR (inset) of trifluoromethylated Tritan®. ^{19}F NMR included fluorobenzene as an internal standard (-113.15 ppm).

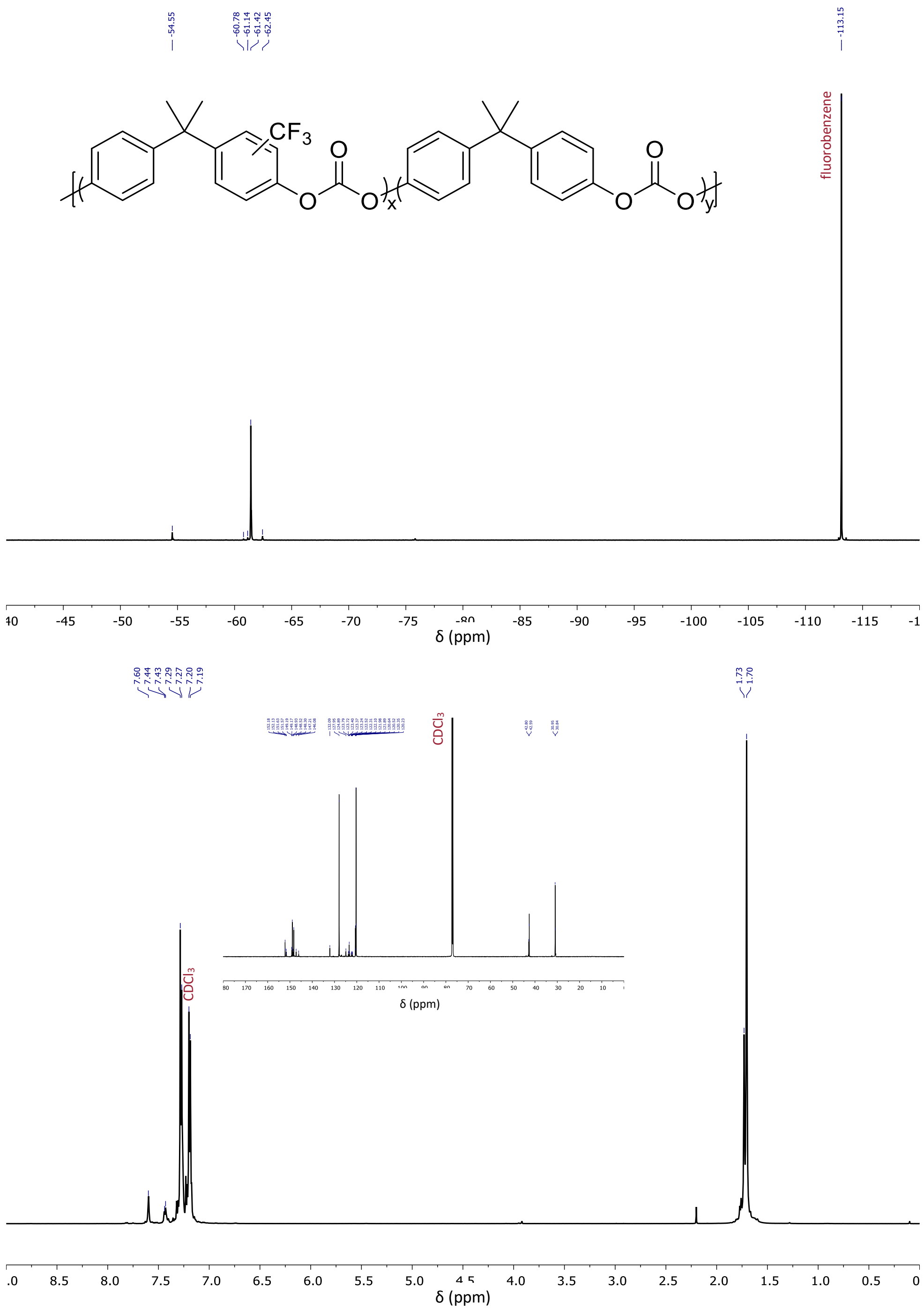

Figure S28: ^{19}F NMR (top), ^{1}H NMR (bottom), and ^{13}C NMR (inset) of trifluoromethylated poly(bisphenol A carbonate). ^{19}F NMR included fluorobenzene as an internal standard (-113.15 ppm).

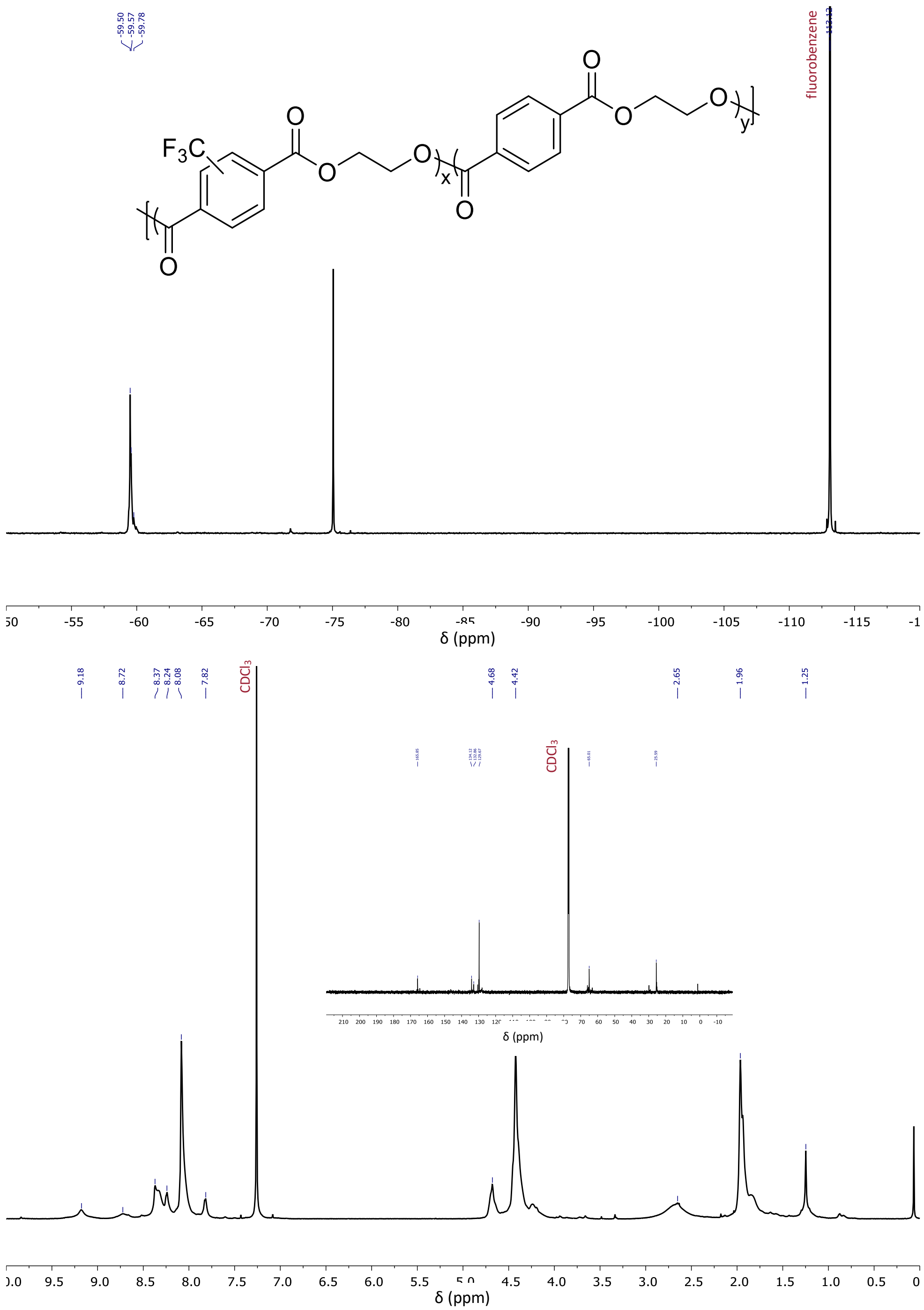

Figure S29: ^{19}F NMR (top), ^{1}H NMR (bottom), and ^{13}C NMR (inset) of trifluoromethylated poly(ethylene terephthalate). ^{19}F NMR included fluorobenzene as an internal standard (-113.15 ppm).

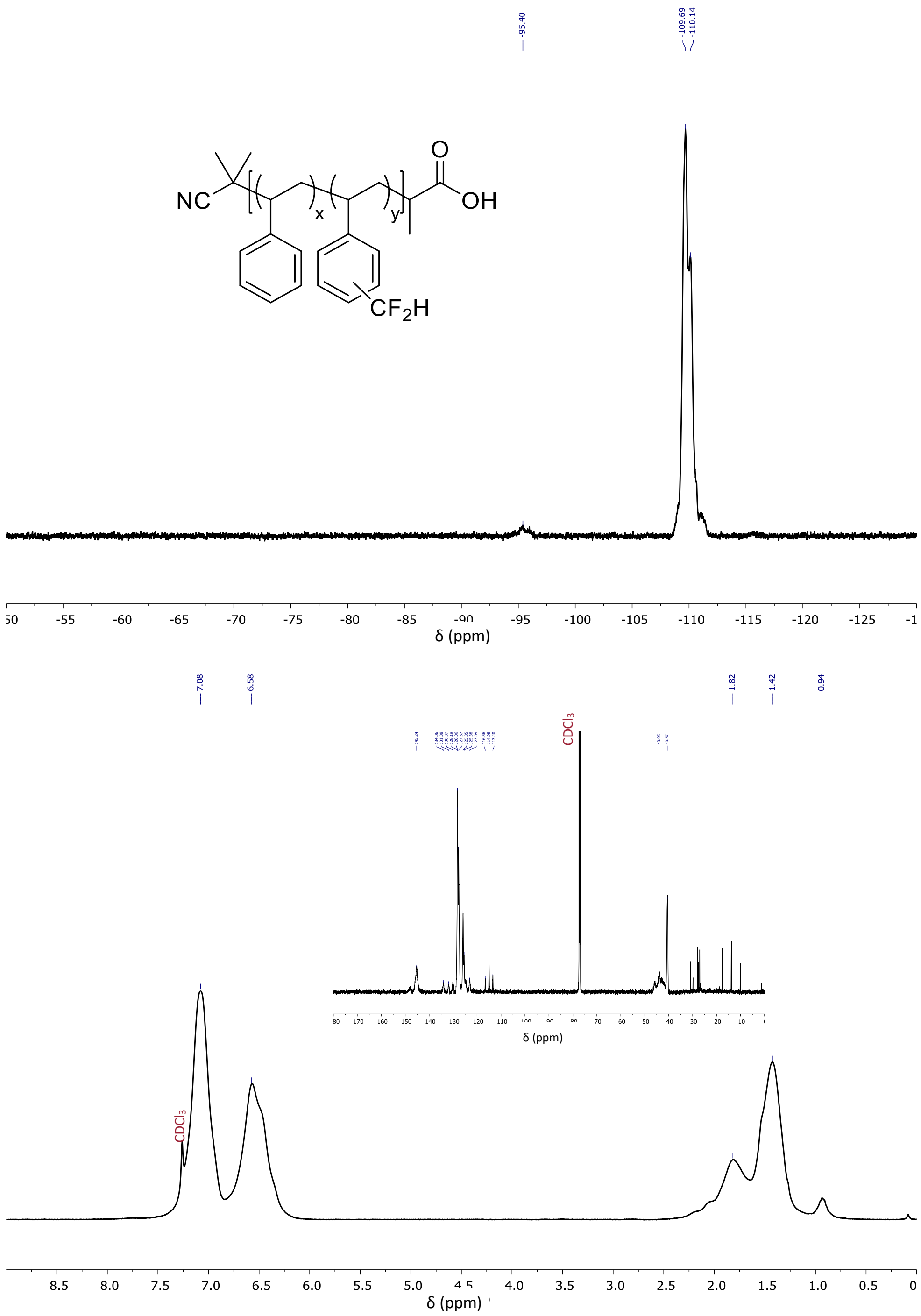

Figure S30: ${}^{19}F$ NMR (top), ${}^{1}H$ NMR (bottom), and ${}^{13}C$ NMR (inset) of difluoromethylated polystyrene.

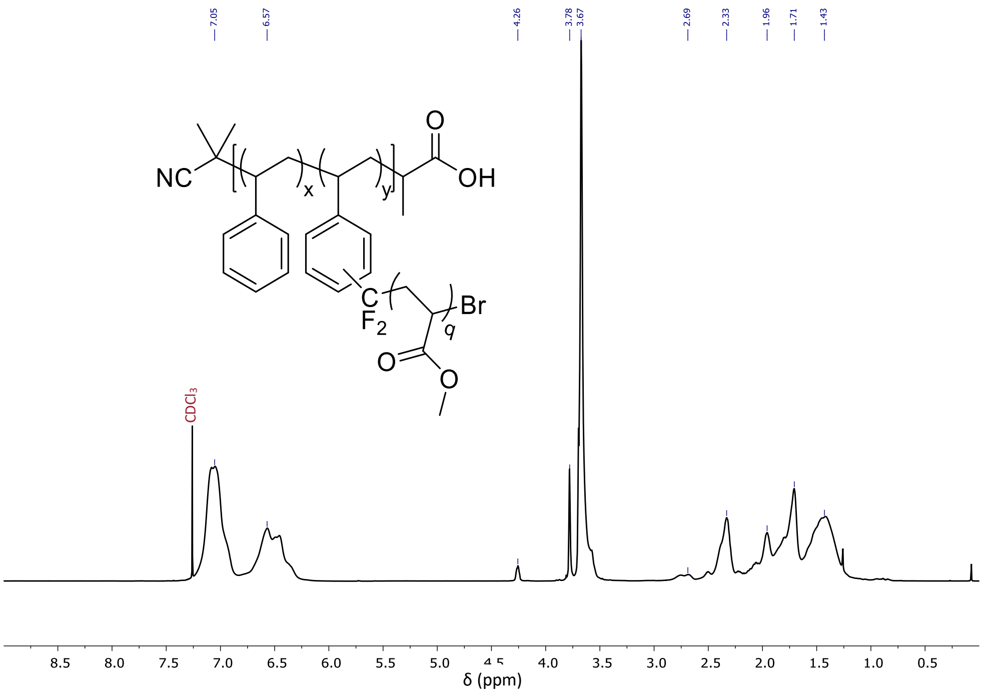
7.05
6.57
4.26
3.78
3.67
2.69
2.33
1.96
1.71
1.43
NC
x
y
O
OH
C
F2
q
Br
O
O
CDCl3
8.5 8.0 7.5 7.0 6.5 6.0 5.5 5.0 4.0 3.5 3.0 2.5 2.0 1.5 1.0 0.5
δ (ppm)

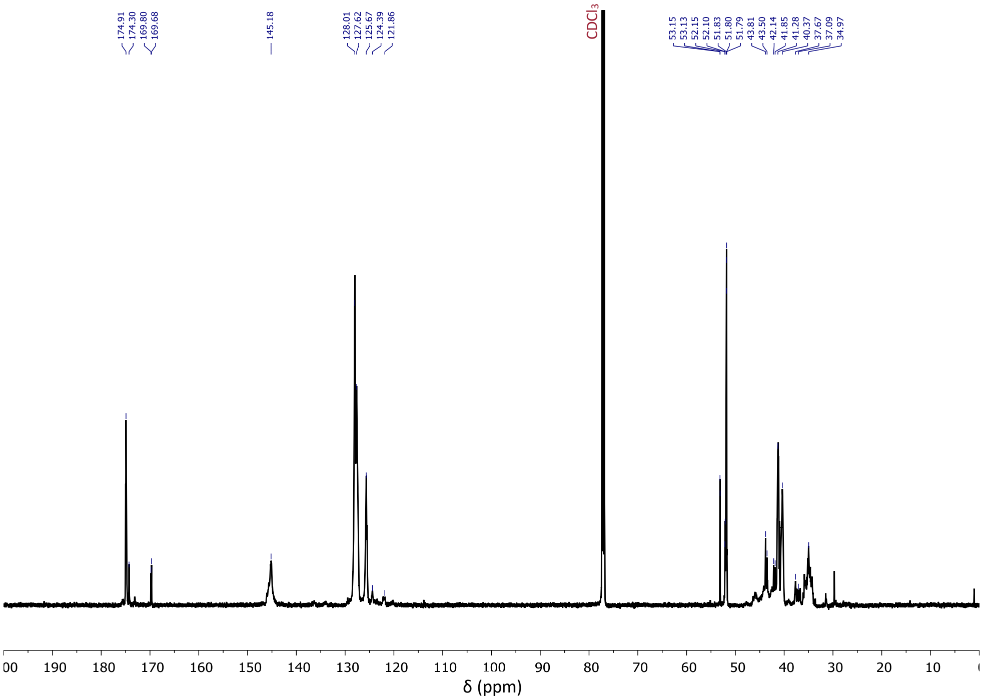
174.91
174.30
169.80
169.68
145.18
128.01
127.62
125.67
124.39
121.86
CDCl3
53.15
53.13
52.15
52.10
51.83
51.80
51.79
43.81
43.50
42.14
41.85
41.28
40.37
37.67
37.09
34.97
190 180 170 160 150 140 130 120 110 100 90 80 70 60 50 40 30 20 10
δ (ppm)

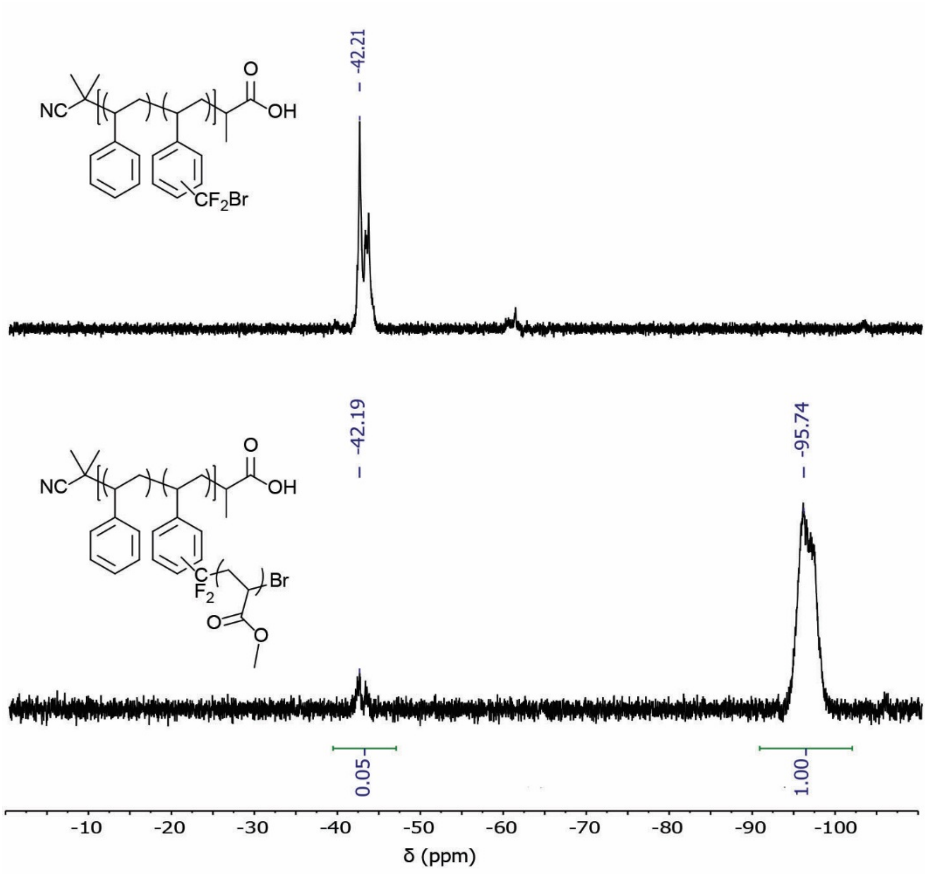

Figure S31: ^{1}H NMR (top), ^{13}C NMR (middle), and ^{19}F NMR (bottom) of poly(styrene-*graft*-methyl acrylate). ^{19}F NMR included fluorobenzene as an internal standard (-113.15 ppm). The ^{19}F NMR is compared to the starting material, the shift from -42.2 to -95.74 ppm indicates initiation of the grafting-from poly(methyl acrylate chain), from the integrations this polymer has had a 95% initiation efficiency.

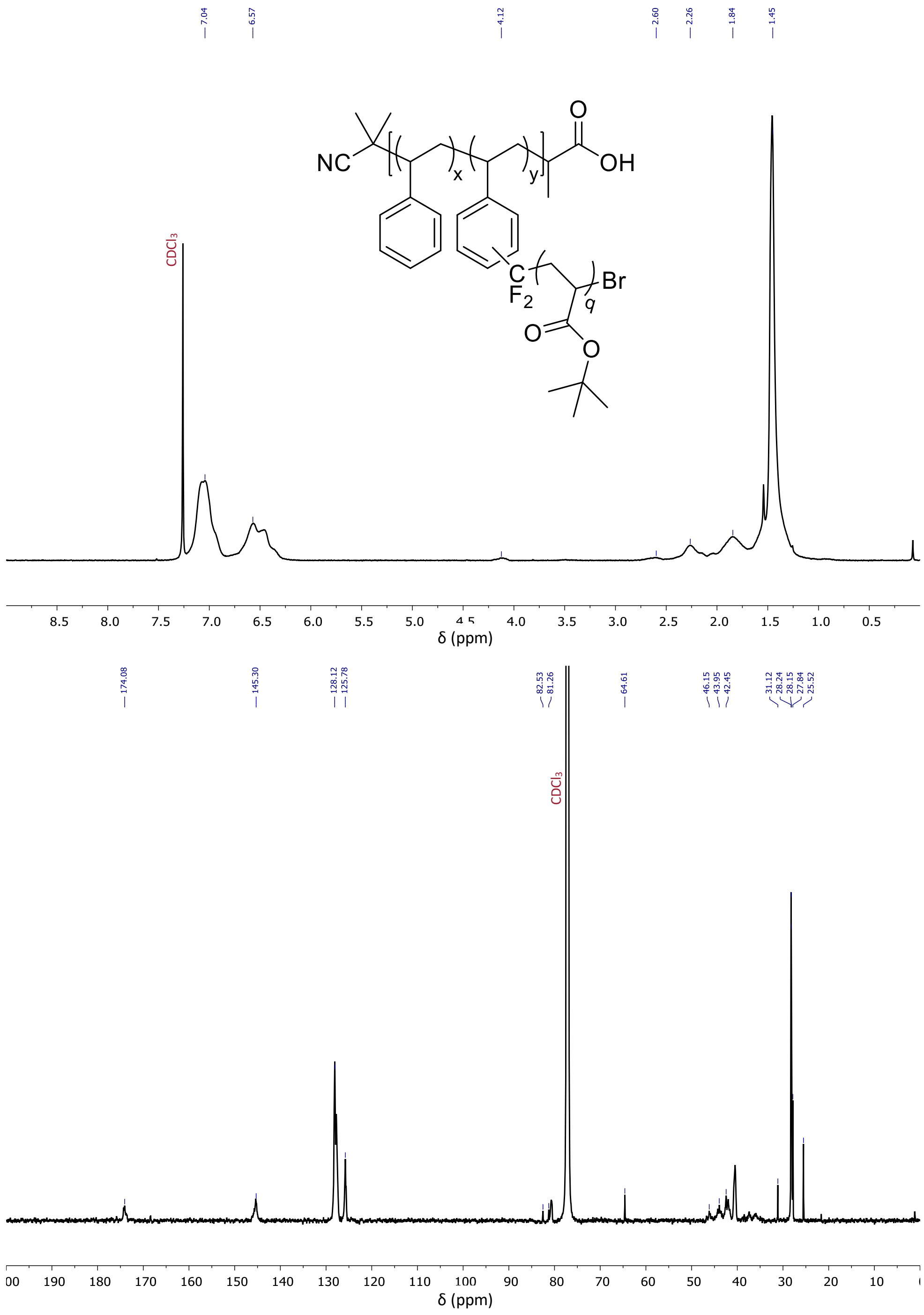

Figure S32: ^{1}H NMR (top), and ^{13}C NMR (bottom) of poly(styrene-*graft*-*tert*-butyl acrylate). ^{19}F NMR included fluorobenzene as an internal standard (-113.15 ppm).

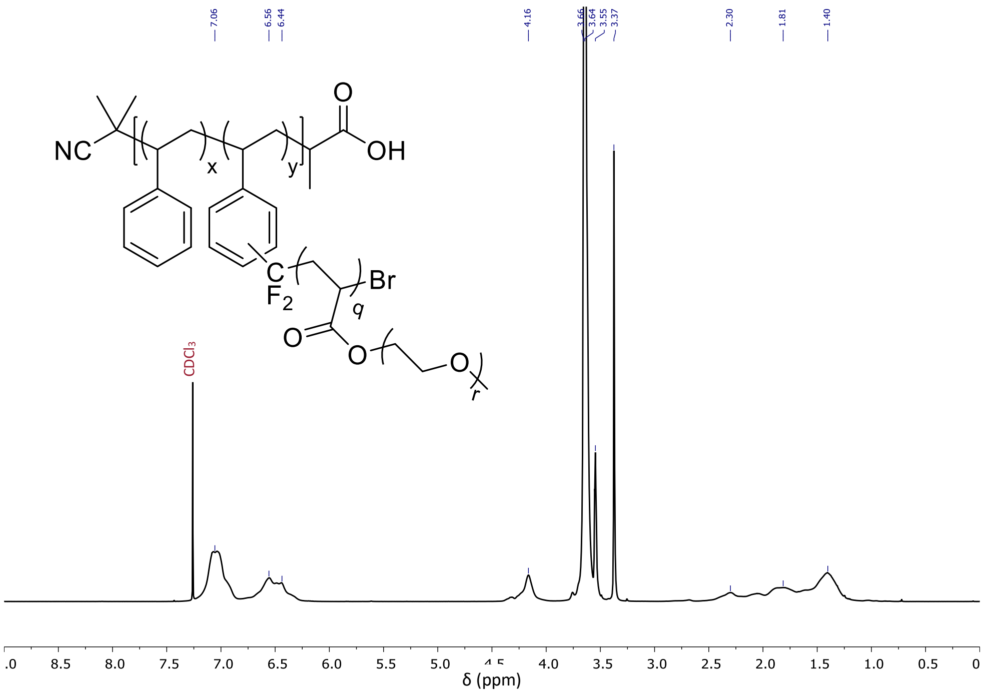

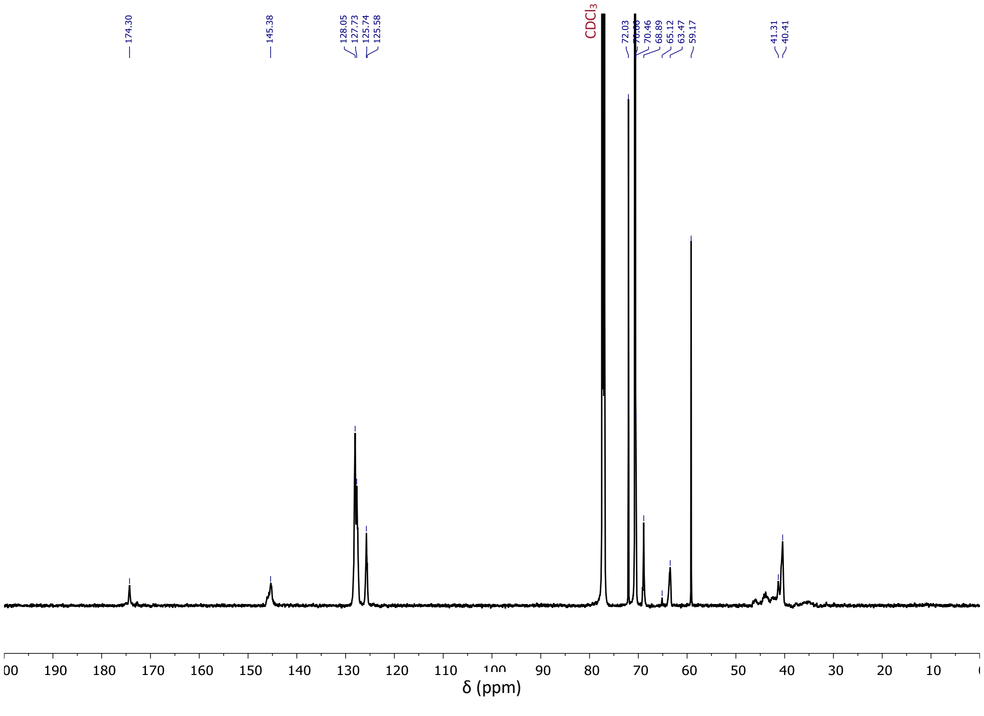

Figure S33: ^{1}H NMR (top), and ^{13}C NMR (bottom) of poly(styrene-*graft*-poly(ethylene glycol) acrylate). ^{19}F NMR included fluorobenzene as an internal standard (-113.15 ppm).

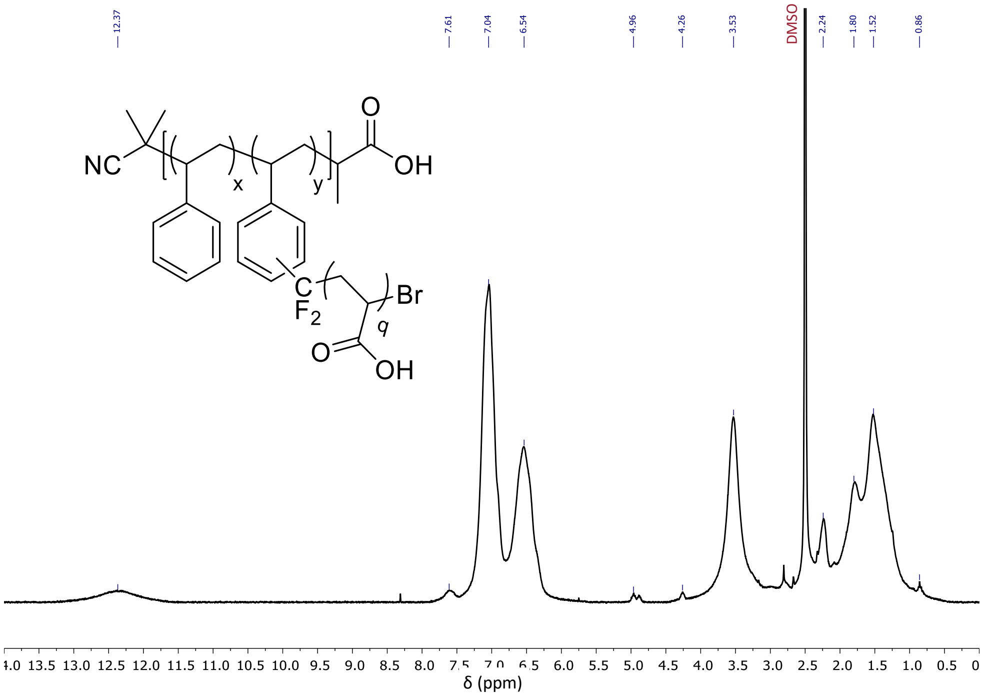

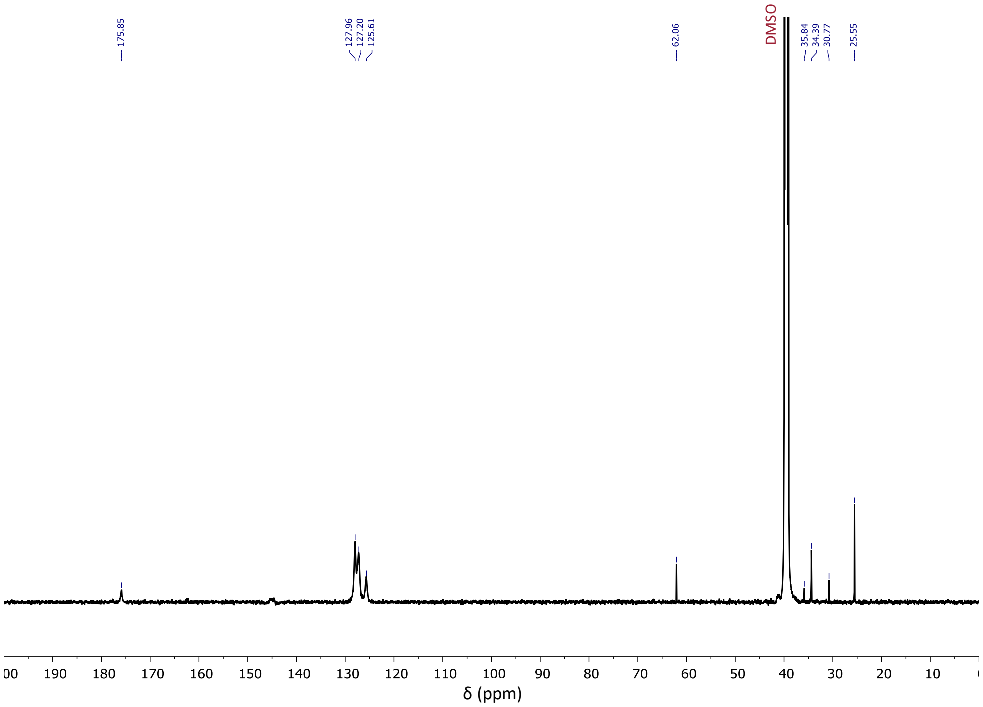

Figure S34: ^{1}H NMR (top), and ^{13}C NMR (bottom) of poly(styrene-*graft*-acrylic acid). ^{19}F NMR included fluorobenzene as an internal standard (-113.15 ppm).

References

1 B. Bömer and H. Hagemann, *Angew. Makromol. Chemie*, 1982, **109**, 285–293.
